# Identification of a seven-miRNA signature as prognostic biomarker for lung squamous cell carcinoma

**DOI:** 10.18632/oncotarget.13164

**Published:** 2016-11-07

**Authors:** Xujie Gao, Yupeng Wu, Wenwen Yu, Hui Li

**Affiliations:** ^1^ Department of Immunology, Tianjin Medical University Cancer Institute and Hospital, Tianjin, China; ^2^ Department of Gastrointestinal Cancer Biology, Tianjin Medical University Cancer Institute and Hospital, Tianjin, China; ^3^ National Clinical Research Center of Cancer, Tianjin, China; ^4^ Key Laboratory of Cancer Immunology and Biotherapy, Tianjin, China

**Keywords:** lung squamous cell carcinoma, microRNA, prognostic marker

## Abstract

**Background:**

Specific biomarkers for outcome prediction of lung squamous cell carcinoma (LUSC) are still lacking. This study assessed the prognostic value of differentially expressed miRNAs of LUSC patients.

**Results:**

Twelve of the 133 most significantly altered miRNAs were associated with overall survival (OS) across different clinical subclasses of the Cancer Genome Atlas (TCGA) LUSC cohort. A linear prognostic model of seven miRNAs was developed to divide patients into high- and low-risk groups. Patients assigned to the high-risk group exhibited poor OS compared with patients in the low-risk group, which was further validated in the validation cohort and entire LUSC cohort.

**Methods:**

MiRNA expression profiles with clinical information of 447 LUSC patients were obtained from TCGA. Most significantly altered miRNAs were identified between tumor and normal samples. Using survival analysis and supervised principal components method, a seven-miRNA signature for prediction of OS of LUSC patients was established. Survival receiver operating characteristic (ROC) analysis was used to assess the performance of survival prediction. The biological relevance of predicted miRNA targets was also analyzed using bioinformatics method.

**Conclusions:**

The current study suggests that seven-miRNA signature may have clinical implications in the outcome prediction of LUSC.

## INTRODUCTION

Lung cancer is the most common and fatal cancer in the world [1]. Non-small cell lung cancer (NSCLC) is the most frequent type of lung cancer and can be further subtyped mainly as adenocarcinomas (LAD) and squamous cell carcinomas (LUSC). Despite decades-long improvements in early detection and treatment, survival of advanced stage patients remains poor [2]. Effective biomarkers to identify patients with high recurrence and death risk are still lacking. Moreover, since the genetic and epigenetic alterations between LUSC and lung LAD are quite different, the targeted agents for LAD cannot be applied to LUSC. Thus, there is an urgent need to identify effective biomarkers for the prognosis of LUSC.

MicroRNAs (miRNAs) are small, non-coding RNAs which regulate gene expression at the posttranscriptional level [3]. By binding to the 3’ [4] or 5’ untranslated region (UTR) [5] of the target transcripts, miRNAs can modulate genes expression through translational repression or cleavage of mRNA. MiRNAs can function as either tumor suppressors or oncogenes by regulating genes involved in tumorigenesis. Moreover, miRNAs have been found in the blood samples and have been demonstrated to be remarkable stable even under harsh conditions such as treatment with RNase and DNase or multiple freeze-thaw cycles [6]. This suggests that miRNAs can be potential noninvasive biomarkers for cancer.

Although a number of miRNAs have been identified for predicting outcome for NSCLC, there is significant inconsistences among previous studies. This may have resulted from the small sample sizes as well as the heterogeneous histological subtypes and tumor stages. Thus, our study aims to identify and assess the prognostic value of candidate miRNA biomarkers for LUSC patients with large cohort.

## RESULTS

### TCGA dataset and patients characteristics

In this study, a total of 447 LUSC patients from The Cancer Genome Atlas (TCGA) were enrolled. 45 normal tissues (n = 45) were also included for differential expression analysis of miRNAs. The patients were separated into the training set (n=224) and testing set (n=223) randomly. No significant difference in clinical covariates was observed between the two sets (Table 1).

**Table 1:** Characteristics of the study population

Variable	Total (n=447)	Training Set (n=224)	Testing Set (n=223)	*P*
Age(yeras)				0.104^*^
< 65	154 (34.5%)	69 (30.8%)	85 (38.1%)	
≥ 65	293 (65.5%)	155 (69.2%)	138 (61.9%)	
Sex				0.569
Male	332 (74.4%)	169 (75.4%)	163 (73.1%)	
Female	115 (25.7%)	55 (24.6%)	60 (26.9%)	
Vital status				0672^†^
Alive	263 (58.8%)	134 (59.8%)	129 (57.8%)	
Dead	184 (41.2%)	90 (40.2%)	94 (42.2%)	
Stage				0.366
I	211 (47.2%)	113 (50.4%)	98 (43.9%)	
II	153 (34.2%)	75(33.5%)	78 (35.0%)	
III	78 (17.4)	33 (14.6%)	45 (20.2%)	
IV	5 (1.1%)	3 (1.3%)	2 (0.9%)	
T stage				0.896
T1	179 (40.0%)	87 (38.8%)	92 (41.3%)	
T2	203 (45.5%)	103 (46.0%)	100 (44.8%)	
T3	55 (12.3%)	28 (12.5%)	27 (12.1%)	
T4	10 (2.2%)	6 (2.7%)	4 (1.8%)	
N stage				0.903
N0	279 (62.4%)	142 (63.4%)	137 (61.4%)	
N1	130 (29.1%)	63 (28.1%)	67 (30.0%)	
N2	35 (7.8%)	18 (8.0%)	17 (7.6%)	
N3	3 (0.7%)	1 (0.4%)	2 (0.9%)	
M stage				1.000
M0	442 (98.9%)	221 (98.7%)	221 (99.1%)	
M1	5 (1.1%)	3 (1.3%)	2 (0.9%)	
Smoke status				0.337
Smoker	293 (65.5%)	142 (63.4%)	151 (67.7%)	
Nonsmoker	154 (34.5%)	82 (36.6%)	72 (32.3)	
Adjuvant treatment				0.279
None	296 (66.2%)	154 (68.8%)	142 (63.7%)	
Chemotherapy	94 (21.0%)	44 (19.6%)	50 (22.4)	
Radiotherapy	16 (3.6%)	10 (4.5%)	6 (2.7%)	
Chemoradiotherapy	41 (9.2%)	16 (7.1%)	25 (11.2%)	

### Identification of dysregulated miRNAs in LUSC

The miRNA expression profiles of 45 pairs of LUSC tumor tissues with normal lung tissues were analyzed. 133 miRNAs were identified differentially expressed miRNAs with *p* value less than 0.05 after FDR adjustment (Supplementary Table S1). Among these, 85 miRNAs were up-regulated and 58 miRNAs were down-regulated.

### Association of miRNAs expression and clinical parameters with overall survival of LUSC patients

We conducted univariate Cox regression assays to identify common miRNAs correlated with overall survival (OS) within each subclass of the following clinical parameters: pathologic N stage, pathologic T stage, and pathologic M stage. MiRNAs were selected if they were significantly correlated with OS in at least two subclasses. Twelve miRNAs were identified in this analysis. The hazard ratios (HR) for the association of miRNAs with OS in each category were shown in Table 2.

**Table 2:** MiRNAs associated with prognosis in different clinical subclasses

miRNA	T1-2 HR(95%CI)	T3-4 HR(95%CI)	N0 HR(95%CI)	N1-3 HR(95%CI)	M0 HR(95%CI)	M1 HR(95%CI)
miR-101-2	-	-	-	0.70(0.51-0.96)	0.81(0.68-0.98)	-
miR-1269	-	0.90(0.82-0.98)	-	0.93(0.87-0.99)	-	-
miR-138-1	-	-	0.86(0.75-0.99)	1.20(1.02-1.40)	-	-
miR-139	1.28(1.08-1.51)	-	1.25(1.03-1.51)	--	1.17(1.01-1.35)	-
miR-144	-	-	0.87(0.77-0.98)	-	0.89(0.81-0.98)	-
miR-182	0.82(0.71-0.96)	-	-	0.75(0.62-0.92)	0.85(0.74-0.97)	-
miR-183	0.86(0.74-0.99)	-	-	0.77(0.62-0.94)	-	-
miR-190	0.82(0.70-0.97)		0.80(0.64-0.99)	-	0.85(0.73-0.98)	-
miR-195	-	0.58(0.38-0.87)	-	0.73(0.56-0.97)	-	-
miR-326	1.13(1.02-1.26)	-	-	1.17(1.02-1.33)	1.13(1.03-1.25)	-
miR-451	-	-	0.87(0.77-0.98)	-	0.91(0.83-0.99)	-
miR-944	-	-	-	0.91(0.83-0.98)	0.93(0.88-0.99)	-

### Establishment of miRNA prognostic model

**Figure 1:**
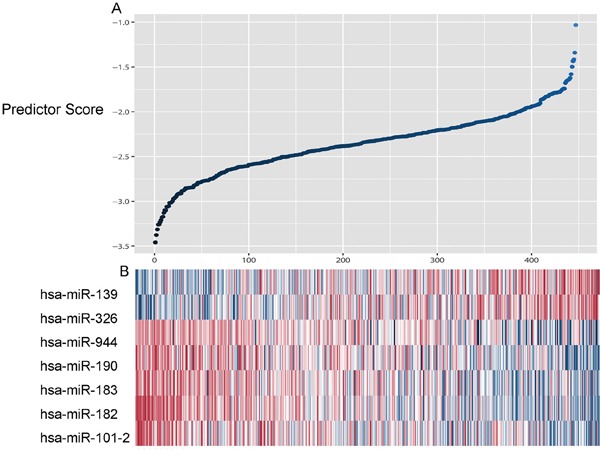
Heatmap and predictor-score of the seven-microRNA signature in LUSC cohort **A.** MicroRNA predictor-score distribution. **B.** Heatmap of the seven-miRNA expression profiles in LUSC patients.

By using the supervised principal component (SPC) method, seven of the 12 miRNAs identified above were selected within the training set. Of these 7 miRNAs, 2 (hsa-mir-139, hsa-mir-326) were negatively correlated with survival, while the other 5 (miR-101-2, miR-182, miR-183, miR-190, hsa-miR-944) were protective. Next, we developed a miRNA prognostic model using the miRNA expression levels based on the log2 reads per million of total aligned miRNA reads. Risk-score = (-0.226 × expression value of hsa-miR-101-2) + (0.147 × expression value of hsa-miR-139) + (-0.084 × expression value of hsa-miR-182) + (-0.026 × expression value of hsa-miR-183) + (-0.125 × expression value of hsa-miR-190) + (0.036 × expression value of hsa-miR-326) + (-0.032 × expression value of hsa-miR-944). The 447 patients were separated into high-risk group or low-risk group using the optimum cutoff point of miRNA scores according to receiver operating characteristic (ROC) curve for predicting 5-year survival in the training set. As shown in Figure 1, high-risk miRNAs tended to up-regulated in patients with high scores, whereas protective miRNAs tended to high expressed in patients with low scores (Figure 1A and 1B).

### Prognostic value of the seven- microRNA signature in LUSC

**Figure 2:**
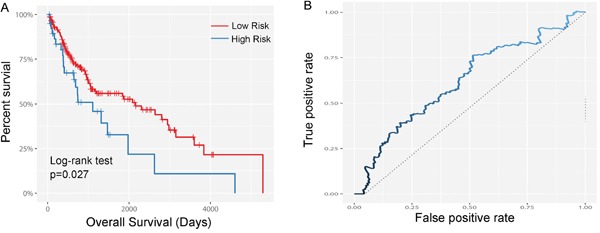
Kaplan–Meier and ROC curves for the seven-miRNA signature in testing set **A.** The Kaplan–Meier curves for testing set (n = 223) divided by the optimum cutoff point. Patients with high scores had poorer outcome in terms of OS (Median OS: 629.6 days vs. 358.3 day, *p*=0.027. **B.** The ROC curve for predicting 60 months survival in the testing set.

**Figure 3:**
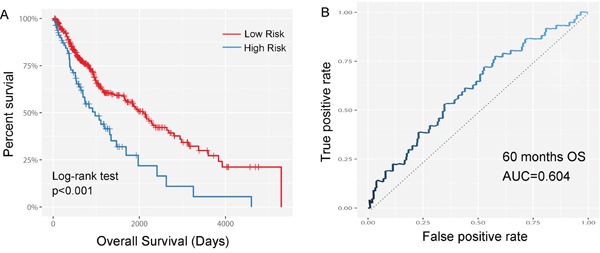
Kaplan–Meier and ROC curves for the seven-miRNA signature in LUSC cohort **A.** The Kaplan–Meier curves for entire LUSC cohort divided by the optimum cutoff point. Patients with high scores had poorer outcome in terms of OS (Median OS: 2086 days vs. 947 day, *p*<0.001. **B.** The ROC curve for predicting 60 months survival in the LUSC cohort.

The ability of prognostic prediction of the seven-miRNA signature was then tested in the testing set and the entire LUSC cohort, respectively. Kaplan-Meier analysis revealed that patients in high-score group had poorer OS in both testing set (*p*=0.027, Figure 2A) and the entire LUSC cohort (*p*<0.001, Figure 3A).

Time-dependent ROC curves were also applied to evaluate the prognostic value of the signature model. The area under ROC curve (AUC) of signature model for the testing set and the entire LUSC cohort was 0.604 (Figure 2B) and 0.610 (Figure 3B), respectively.

We also assessed the prognostic power of the seven-miRNA signature in early stage patients (stage I and II patients, n = 364). Kaplan-Meier analysis revealed that patients in high-risk group are associated with worse OS (*p*<0.001, log-rank test).

Finally, multivariate Cox regression analysis were used to investigate the independent prognostic value of the seven-miRNA signature. Age, gender, T stage, N stage and the miRNA signature were used as covariates. The miRNA signature (HR = 1.965, *p* < 0.001) and T stage (HR = 1.684, *p* =0.004) are showed to be as independent prognostic factors related with OS (Table 3).

**Table 3:** Multivariate analysis of overall survival of patients

Characteristic	HR(95%CI)	P value
Sex (male vs. female)	0.800(0.566-1.133)	0.209
Age (< 65 vs. ≥ 65 years)	1.262(0.908-1.755)	0.166
Smoking status	0.995(0.735-1.347)	0.995
T stage (T1-2 vs T3-4)	1.705(1.200-2.424)	**0.003**
N stage (N0 vs N1-3)	0.773(0.421-1.419)	0.406
miRNA signature	2.809(1.812-4.355)	**<0.001**

### Correlation between miRNA signature and clinical characteristics

We examined the association of seven-miRNA signature with clinical factors in LUSC. No significant differences were observed when patients were stratified by gender, age and smoke status (Supplementary Table S2).

### *In silico* analysis of target genes and pathways

The target genes of seven miRNAs was downloaded from miRecords. A total of 4242 target genes predicted by more than 4 data sets were selected for further analysis. Next, we performed a functional enrichment analysis to elucidate the biological function of target genes of seven miRNAs. A total of 39 Kyoto Encylopedia of Genes and Genomes (KEGG) pathways and 732 Gene Ontology (GO) pathways were enriched (Supplementary Table S3). The results showed that the predicted target genes involved in many important pathways associated with cancer development, e.g., adherens junction, Wnt, TGF-beta and MAPK signaling pathways (Table 4). Moreover, many target genes were enriched in cancer-related pathways for lung cancer.

**Table 4:** Results of over-representation analysis of the predicted target genes

Pathway	Target gene
Wnt signaling pathway	BTRC, FZD6, JUN, RAC1, WNT1, WNT2
MAPK signaling pathway	FOS, TGFBR2, BDNF CACNA1C, MAPK9
Adherens junction	ACTN4, CREBBP, CTNND1, TJP1, IQGAP1
Apoptosis	FOXO1, IGF1R, MCL1, MITF, NOTCH1, NOTCH2, PTGS2, RARG
TGF-beta signaling pathway	ACVR2B, TGFBR1, TGFBR2
VEGF signaling pathway	ITGA5, VEGFA, ITGB3
Non-small cell lung cancer	CDK6, E2F3, FOXO3, GRB2, KRAS, MAPK1, PIK3R1, PRKCA, RARB, RASSF1, RXRA, RXRB, STK4, TGFA

## DISCUSSION

Accumulating evidence reveals that miRNAs could play a crucial role in tumorigenesis and progression of lung cancer. Many studies have reported the potential of miRNAs as biomarkers for early detection, molecular classification, prediction of outcome and treatment efficacy for lung cancer [7]. Although several studies had identified a number of miRNAs with prognostic value, most of these studies did not accurately analyze the expression of miRNAs in the different histotypes and stages. Since the miRNA expression patterns are quite different in different pathological types and tumor stages, the miRNAs identified in those studies may have less power in the clinical application for lung cancer outcome prediction.

In the current study, from the 133 differential expressed miRNAs, 12 miRNAs associate with OS of LUSC patients were identified in clinical subgroups. By using supervised principal components method, a seven-miRNA (miR-101-2, miR-139, miR-182, miR-183, miR-190, miR-326 and miR-944) signature was established and was validated to be an independent factor for outcome prediction for LUSC patient. This signature was demonstrated with high prognostic ability in both the entire LUSC set and the early stage patients.

Dysregulation of miR-139 has been observed in a variety of cancers [8]. Liu et al. reported that down-regulation of miR-139 was correlated with poor survival of colon cancer patients [9]. MiR-139 regulates diverse biological processes, such as proliferation, invasion and metastasis [8]. Low expression of miR-139 was significantly related with invasiveness and lymph node metastasis of NSCLC [10]. Wang et al. reported that downregulation of miR-101 accelerates the progression of lung cancer in RUNX1 dependent manner [11]. MiR-101 was also reported to suppress lung tumorigenesis through inhibition of DNMT3a [12]. By upregulating miR-101, Curcumin could inhibit the expression of EZH2, thus inhibiting lung cancer growth and metastasis [13]. MiR-190 could downregulate the expression of PH domain leucine-rich repeat protein phosphatase (PHLPP) and enhance the activation of Akt which leads to carcinogenesis [14]. Gennarino et al. reported that miR-190 could inhibit TGF-beta signaling and its effects on cell proliferation, morphology and scattering in NSCLC [15]. Recent studies reported that mir-326 was involved in carcinogenesis [16], metastasis, invasion of tumor[17, 18], and chemotherapy resistance [19]. MiR-326 was also identified as a biomarker for outcome prediction for prostate cancer and gastric cancer [20]. MiR-326 could regulate cell proliferation and migration of lung cancer by targeting phox2a [21], CCND1 [22] and NSBP [23]. Several reports of miR-182 highlighted its crucial roles in various cancers [24–26]. Stenvold et al. reported that up-regulation of miR-182 was associated with a good prognosis in LUSC [27]. By targeting different target genes, miR-182 could inhibit cell proliferation and invasion of LAD and LUSC [28–30]. Zhang et al. reported that serum miR-182 and miR-183 could be an effective biomarker for early diagnosis of NSCLC [31]. Powrozek et al. reported that plasma miR-944 could serve as biomarker for diagnosis of LUSC [32]. Through comprehensive analysis of characterization of LAD and LUSC, miR-944 was identified a potential drive-miRNA in classifying tumor histology [33]. Liu et al. reported that miR-944 affects cell growth by targeting EPHA7 [34] or SOCS4 [35] in NSCLC. Up-regulation of miR-944 is correlated with lymph node metastasis and advanced-stage of LUSC [35].

Based on the public data with large sample size, the prognostic value of miRNAs have been evaluated in other tumor types, including lung adenocarcinoma [36], gliomas [37], bladder cancer [38], pancreatic cancer [39], breast cancer [40], hepatocellular carcinoma [41], head and neck squamous cell carcinomas [42, 43], cervical cancer [44], melanoma [45]. Except miR-326 which have been reported as a member of a seven-miRNA signature predicting survival in hepatocellular carcinoma, the other six miRNAs identified in our study have not been recommended as biomarkers with best prognostic value in other tumor types. These results further supported the specificity of the seven-miRNA signature in predicting outcome for LUSC.

To gain a further insight into the functional role of the seven miRNAs, we retrieved their target genes and analyzed their related pathways. Bioinformatic analysis revealed that some important tumor-related genes are simultaneously by two or more miRNAs. Type 1 insulin-like growth factor receptor (IGF1R) is predicted to be the target gene of three miRNAs (miR-182, miR-326 and miR-944). The over-expression of IGF1R, which mediates tumor growth, adhesion, and protection from apoptosis, has been observed in various cancers [46]. ATRX is the predicted target of miR-101, miR-139 and miR-944. Misexpression of ATRX could modify the histone variant composition of chromatin by promoting genomic instability or gene expression changes related with tumorigenesis [47]. In addition, we also found that these miRNAs could regulate several crucial signaling pathways. Dysregulation of TGF-beta signaling pathway is important in cancer progression and cell invasion. Pajares et al. found that TGF-beta-induced protein expression was an independent outcome predictor for adjuvant-treated LUSC patients. Our informatics analysis identified several target genes that are involved in TGF-beta signaling pathway. Yang et al. found that high expression levels of VEGF-B is associated poor survival of LUSC patients. Three target genes related to VGEF signaling pathway have been identified. However, further molecular investigations are needed to confirm these predictions.

Nevertheless, some limitations may exist in the current study. Firstly, the censored rate of TCGA LUSC dataset was relatively high. This may have an impact on the reliability of the survival analysis. Secondly, the value of the miRNA signature should be validated in other independent cohort with long-term follow up.

In summary, our current study identified a seven-miRNA signature as potential outcome predictor for LUSC patients. Future studies using independent cohorts of large sample size from multiple institutions are needed to validate our findings for clinical practice. Future functional investigations are also required to explore the underlying mechanisms of these miRNAs in LUSC development.

## MATERIALS AND METHODS

### TCGA dataset

The LUSC dataset, including Level 3 miRNA expression data with clinical information, was downloaded from TCGA data portal (March 2016). To exclude unrelated causes of death, cases with less than 1-month follow-up less were excluded in the subsequent analysis.

### Identification of dysregulated miRNAs in LUSC

The raw counts of miRNA expression data (Illumina HiSeq Systems) of 45 LUSC with their paired normal tissue were obtained from the TCGA dataset. MiRNA-expression data was normalized by using the R/Bioconductor package edgeR, which is designed for digital gene expression data [48]. MiRNAs with logFC (log2 fold change) < −1 or >1 (*p* less than 0.05, after FDR adjusted) were considered as differentially expressed miRNAs and were selected for further analysis.

### Identification of miRNAs with prognostic value in LUSC

Semi-supervised method which combines the gene expression profile with clinical imformation was used to conduct univariate Cox regression analyses [49, 50]. Common miRNAs associated with OS were identified within each of the subgroups stratified by the TNM system. Common miRNAs identified in at least two independent subclasses were selected for the subsequent studies, using a HR>1 or HR<1 with p<0.05 as the cutoff.

### Definition of prognostic risk model and ROC curve analysis

An importance score was calculated by the SPC method and was assigned to each miRNA [49]. Ten-fold cross validation was used to calculate the best threshold in SPC model and to select significant miRNAs. The TCGA dataset was separated into the training group and the testing group randomly. The linear signature prognostic model was developed based on the SPC method. Then, using the prognostic model, risk scores were compute for all the 447 patients. The best cutoff value of prognostic score was decided in the ROC curve analysis for predicting 5-year survival of the training set. The OS curves were evaluated using the Kaplan–Meier and log-rank method. Time-dependent ROC curves were also applied to assess the predict power of the prognostic model. All analyses were performed by the R/BioConductor (version 3.3.1).

### Bioinformatic analysis of miRNA-target genes and pathways

Potential target genes of the candidate miRNAs were obtained from miRecords v4.0 (www.mirecords.biolead.org) database, which offers a comprehensive data of possible miRNA targets of 11 different data sets. The pathway enrichment analysis was conducted with the GeneTrail gene set enrichment tool. The results were considered significant when p value was less than 0.05 after FDR corrected [36] [51].

## SUPPLEMENTARY MATERIALS FIGURES AND TABLES




