# Supplementary material for: Identification of a seven-miRNA signature as prognostic biomarker for lung squamous cell carcinoma
Source: Oncotarget. 2016 Nov 7;7(49):81670–9. doi: 10.18632/oncotarget.13164 (PMC5348421; doi:10.18632/oncotarget.13164)
Supplement: Supplementary file 1 [file oncotarget-07-81670-s001.pdf]

Identification of a seven-miRNA signature as prognostic biomarker for lung squamous cell carcinoma

SUPPLEMENTARY TABLES

Supplementary Table S1: The differentially expressed miRNAs in paired LUSC samples with normal samples

See Supplementary File 1

Supplementary Table S2: The relationship of 7-miRNA signature and clinical parameters

Variable	High risk	Low risk	*P*
Age(years)			0.787
< 65	30 (19.5%)	124 (80.5%)	
≥ 65	54 (18.4%)	239 (81.6%)	
Sex			0.136
Male	57(17.2%)	275 (82.8%)	
Female	27 (23.5%)	88 (76.5%)	
Smoke status			0.811
Smoker	56 (19.1%)	237 (80.9%)	
Nonsmoker	28 (18.2%)	126 (81.8%)	

Supplementary Table S3: The over-representation analysis for target genes

See Supplementary File 1
