# Supplementary material for: Identification of a seven-miRNA signature as prognostic biomarker for lung squamous cell carcinoma
Source: Oncotarget. 2016 Nov 7;7(49):81670–9. doi: 10.18632/oncotarget.13164 (PMC5348421; doi:10.18632/oncotarget.13164)
Supplement: Supplementary file 2 [file oncotarget-07-81670-s002.docx]

**Supplementary Table S1. The differentially expressed miRNAs in paired LUSC samples with normal samples.**

| **miRNA** | **logFC*** | **logCPM*** | **LR*** | **P Value** | **FDR** |
| --- | --- | --- | --- | --- | --- |
| hsa-let-7c | -1.59416 | 11.1291 | 13.02788 | 0.000307 | 0.000712 |
| hsa-let-7f-1 | -1.00081 | 4.497586 | 49.3753 | 2.11E-12 | 1.05E-11 |
| hsa-mir-100 | -1.66586 | 13.29161 | 21.76359 | 3.08E-06 | 9.00E-06 |
| hsa-mir-101-1 | -1.8875 | 14.76562 | 60.65901 | 6.79E-15 | 4.42E-14 |
| hsa-mir-101-2 | -2.16125 | 6.351579 | 248.5652 | 5.34E-56 | 5.30E-54 |
| hsa-mir-103-2 | 1.034495 | 3.755315 | 51.48219 | 7.22E-13 | 3.77E-12 |
| hsa-mir-105-1 | 4.817074 | 4.425138 | 49.23444 | 2.27E-12 | 1.11E-11 |
| hsa-mir-105-2 | 5.031169 | 4.43804 | 63.69557 | 1.45E-15 | 1.15E-14 |
| hsa-mir-10a | -1.01968 | 15.05485 | 20.25529 | 6.78E-06 | 1.87E-05 |
| hsa-mir-1-2 | -3.2471 | 4.370639 | 159.354 | 1.57E-36 | 5.65E-35 |
| hsa-mir-1226 | 1.064731 | 1.715648 | 14.21447 | 0.000163 | 0.000388 |
| hsa-mir-1247 | -1.47415 | 5.434472 | 31.6065 | 1.89E-08 | 6.94E-08 |
| hsa-mir-1248 | 1.969185 | 2.124608 | 54.89583 | 1.27E-13 | 7.64E-13 |
| hsa-mir-1249 | 1.352309 | 1.924696 | 27.53819 | 1.54E-07 | 4.93E-07 |
| hsa-mir-126 | -1.9279 | 12.54129 | 22.01554 | 2.70E-06 | 8.01E-06 |
| hsa-mir-1269 | 7.963114 | 8.442024 | 208.272 | 3.27E-47 | 2.60E-45 |
| hsa-mir-1271 | 1.131007 | 2.531298 | 34.70687 | 3.83E-09 | 1.55E-08 |
| hsa-mir-129-1 | 1.869515 | 3.387118 | 32.03527 | 1.51E-08 | 5.62E-08 |
| hsa-mir-129-2 | 1.975156 | 3.44354 | 40.12821 | 2.38E-10 | 1.05E-09 |
| hsa-mir-1293 | 3.668549 | 1.958316 | 53.32601 | 2.83E-13 | 1.60E-12 |
| hsa-mir-1301 | 1.212531 | 3.898675 | 51.78174 | 6.20E-13 | 3.28E-12 |
| hsa-mir-1306 | 1.69888 | 3.787826 | 122.241 | 2.04E-28 | 3.86E-27 |
| hsa-mir-1307 | 1.254021 | 10.67399 | 9.11625 | 0.002533 | 0.00508 |
| hsa-mir-130b | 2.427035 | 5.239086 | 170.3151 | 6.31E-39 | 2.51E-37 |
| hsa-mir-133a-1 | -2.99182 | 4.313643 | 151.1824 | 9.56E-35 | 2.71E-33 |
| hsa-mir-135b | 1.173235 | 4.487713 | 29.48807 | 5.63E-08 | 1.93E-07 |
| hsa-mir-137 | 2.483396 | 1.748081 | 28.81013 | 7.98E-08 | 2.64E-07 |
| hsa-mir-138-1 | -1.43628 | 2.627326 | 29.9941 | 4.33E-08 | 1.51E-07 |
| hsa-mir-138-2 | -1.43199 | 2.404332 | 25.83016 | 3.73E-07 | 1.16E-06 |
| hsa-mir-139 | -2.77088 | 6.868929 | 180.1608 | 4.47E-41 | 1.97E-39 |
| hsa-mir-140 | -1.98126 | 10.95468 | 21.34015 | 3.85E-06 | 1.11E-05 |
| hsa-mir-141 | 1.26704 | 10.18344 | 9.204401 | 0.002414 | 0.00489 |
| hsa-mir-143 | -1.62165 | 16.75552 | 741.0703 | 3.51E-163 | 6.96E-161 |
| hsa-mir-144 | -3.74522 | 9.338845 | 61.33753 | 4.81E-15 | 3.24E-14 |
| hsa-mir-145 | -1.46031 | 11.25167 | 11.51317 | 0.000691 | 0.001516 |
| hsa-mir-1468 | -1.00478 | 3.04404 | 28.38266 | 9.96E-08 | 3.24E-07 |
| hsa-mir-146b | -1.26029 | 10.3411 | 9.431 | 0.002133 | 0.004389 |
| hsa-mir-149 | 2.689386 | 6.944388 | 136.7522 | 1.37E-31 | 3.01E-30 |
| hsa-mir-150 | -1.1403 | 10.34198 | 5.947738 | 0.014736 | 0.025659 |
| hsa-mir-181a-1 | -1.36291 | 12.11062 | 11.79203 | 0.000595 | 0.001334 |
| hsa-mir-182 | 1.42085 | 13.72248 | 16.80836 | 4.14E-05 | 0.000103 |
| hsa-mir-183 | 2.289146 | 13.03939 | 32.88615 | 9.77E-09 | 3.73E-08 |
| hsa-mir-184 | -1.91077 | 4.904213 | 23.31051 | 1.38E-06 | 4.18E-06 |
| hsa-mir-190 | -1.35557 | 2.903676 | 54.00754 | 2.00E-13 | 1.15E-12 |
| hsa-mir-1911 | 2.145254 | 2.830671 | 16.65627 | 4.48E-05 | 0.000111 |
| hsa-mir-193b | 1.879941 | 6.576055 | 91.50876 | 1.11E-21 | 1.47E-20 |
| hsa-mir-195 | -1.99424 | 5.965457 | 152.2292 | 5.65E-35 | 1.72E-33 |
| hsa-mir-196a-1 | 4.923341 | 6.281442 | 183.053 | 1.04E-41 | 5.18E-40 |
| hsa-mir-196a-2 | 4.716648 | 2.56156 | 200.6073 | 1.54E-45 | 8.73E-44 |
| hsa-mir-196b | 4.381079 | 8.110024 | 146.3803 | 1.07E-33 | 2.50E-32 |
| hsa-mir-1976 | -1.19758 | 4.526587 | 79.60008 | 4.58E-19 | 4.79E-18 |
| hsa-mir-200a | 1.422948 | 9.436515 | 13.93653 | 0.000189 | 0.000447 |
| hsa-mir-203 | 1.606919 | 14.67937 | 18.43374 | 1.76E-05 | 4.53E-05 |
| hsa-mir-205 | 6.897742 | 12.82945 | 132.4286 | 1.21E-30 | 2.52E-29 |
| hsa-mir-210 | 4.730295 | 10.5233 | 77.24603 | 1.51E-18 | 1.46E-17 |
| hsa-mir-218-1 | -1.58107 | 2.219841 | 52.14703 | 5.15E-13 | 2.84E-12 |
| hsa-mir-218-2 | -2.07808 | 7.23391 | 148.7133 | 3.31E-34 | 8.22E-33 |
| hsa-mir-219-1 | 1.116735 | 2.538105 | 32.44581 | 1.23E-08 | 4.63E-08 |
| hsa-mir-223 | -1.43404 | 9.63079 | 11.24865 | 0.000797 | 0.001729 |
| hsa-mir-224 | 2.264968 | 6.727 | 82.7154 | 9.48E-20 | 1.04E-18 |
| hsa-mir-2277 | 1.913343 | 1.889334 | 48.16024 | 3.93E-12 | 1.90E-11 |
| hsa-mir-26a-2 | -1.35374 | 11.5176 | 10.81835 | 0.001005 | 0.002168 |
| hsa-mir-296 | 1.453176 | 3.627495 | 29.61605 | 5.27E-08 | 1.82E-07 |
| hsa-mir-29a | -1.4509 | 13.26363 | 18.60836 | 1.61E-05 | 4.19E-05 |
| hsa-mir-29c | -1.85237 | 11.92634 | 19.71508 | 8.99E-06 | 2.41E-05 |
| hsa-mir-301a | 1.248762 | 4.075129 | 57.80631 | 2.89E-14 | 1.85E-13 |
| hsa-mir-301b | 3.143031 | 2.149124 | 112.105 | 3.39E-26 | 5.61E-25 |
| hsa-mir-3065 | -2.12929 | 7.505816 | 86.81065 | 1.19E-20 | 1.44E-19 |
| hsa-mir-30a | -3.45555 | 16.09079 | 268.3184 | 2.64E-60 | 3.49E-58 |
| hsa-mir-30b | -2.10157 | 9.449164 | 30.2661 | 3.77E-08 | 1.32E-07 |
| hsa-mir-30c-2 | -1.43535 | 9.23778 | 18.14238 | 2.05E-05 | 5.25E-05 |
| hsa-mir-30d | -2.45473 | 14.18463 | 61.27764 | 4.96E-15 | 3.28E-14 |
| hsa-mir-31 | 4.238567 | 4.534087 | 129.9416 | 4.22E-30 | 8.38E-29 |
| hsa-mir-3127 | 1.026989 | 2.727966 | 34.32465 | 4.66E-09 | 1.85E-08 |
| hsa-mir-3200 | 1.883542 | 2.903379 | 68.01128 | 1.63E-16 | 1.47E-15 |
| hsa-mir-320b-2 | 1.106992 | 2.878974 | 35.2783 | 2.86E-09 | 1.19E-08 |
| hsa-mir-323 | 1.532692 | 2.657063 | 22.49082 | 2.11E-06 | 6.35E-06 |
| hsa-mir-323b | 2.541228 | 2.565971 | 67.12317 | 2.55E-16 | 2.20E-15 |
| hsa-mir-326 | -1.91853 | 4.590196 | 66.43073 | 3.62E-16 | 3.06E-15 |
| hsa-mir-331 | 1.003417 | 5.305208 | 52.61983 | 4.05E-13 | 2.26E-12 |
| hsa-mir-338 | -2.80854 | 10.68145 | 35.02268 | 3.26E-09 | 1.33E-08 |
| hsa-mir-33a | 1.74719 | 5.295182 | 63.34588 | 1.73E-15 | 1.35E-14 |
| hsa-mir-33b | 2.043629 | 2.85571 | 70.3879 | 4.87E-17 | 4.50E-16 |
| hsa-mir-345 | 2.07331 | 4.478669 | 99.71803 | 1.76E-23 | 2.58E-22 |
| hsa-mir-34b | -1.40259 | 5.208234 | 13.06658 | 0.000301 | 0.000702 |
| hsa-mir-34c | -1.74122 | 7.806404 | 20.72949 | 5.29E-06 | 1.47E-05 |
| hsa-mir-3648 | 2.773606 | 2.431047 | 118.0126 | 1.72E-27 | 3.11E-26 |
| hsa-mir-3651 | 1.78856 | 1.756331 | 35.18724 | 2.99E-09 | 1.24E-08 |
| hsa-mir-3653 | 1.002875 | 3.105224 | 37.93579 | 7.31E-10 | 3.15E-09 |
| hsa-mir-3676 | 1.27176 | 3.023033 | 28.59245 | 8.93E-08 | 2.93E-07 |
| hsa-mir-3677 | 1.150271 | 3.032957 | 30.66944 | 3.06E-08 | 1.09E-07 |
| hsa-mir-3687 | 2.723657 | 1.738166 | 65.75855 | 5.10E-16 | 4.22E-15 |
| hsa-mir-375 | -2.67147 | 13.28941 | 30.74255 | 2.95E-08 | 1.06E-07 |
| hsa-mir-3926-1 | -1.16568 | 1.82399 | 21.58074 | 3.39E-06 | 9.83E-06 |
| hsa-mir-3928 | 1.800703 | 1.900075 | 42.89529 | 5.77E-11 | 2.64E-10 |
| hsa-mir-421 | 1.154128 | 2.479527 | 33.01847 | 9.13E-09 | 3.52E-08 |
| hsa-mir-429 | 1.038133 | 6.555532 | 40.62621 | 1.84E-10 | 8.22E-10 |
| hsa-mir-4326 | 1.314985 | 2.834935 | 51.18355 | 8.41E-13 | 4.34E-12 |
| hsa-mir-451 | -3.61084 | 11.50031 | 46.83145 | 7.74E-12 | 3.66E-11 |
| hsa-mir-455 | 1.853012 | 8.22265 | 112.7335 | 2.47E-26 | 4.26E-25 |
| hsa-mir-486 | -3.49777 | 9.667066 | 51.85492 | 5.98E-13 | 3.21E-12 |
| hsa-mir-487a | 1.002647 | 1.675477 | 9.733647 | 0.001809 | 0.0038 |
| hsa-mir-497 | -1.49086 | 5.307966 | 87.53725 | 8.27E-21 | 1.03E-19 |
| hsa-mir-503 | 1.612332 | 2.601648 | 61.99983 | 3.43E-15 | 2.44E-14 |
| hsa-mir-511-1 | -1.73691 | 4.387678 | 93.01946 | 5.18E-22 | 7.09E-21 |
| hsa-mir-511-2 | -1.78696 | 4.400922 | 84.04983 | 4.82E-20 | 5.47E-19 |
| hsa-mir-548b | -1.61922 | 2.309553 | 43.94972 | 3.37E-11 | 1.56E-10 |
| hsa-mir-548v | 1.027656 | 1.62362 | 11.79439 | 0.000594 | 0.001334 |
| hsa-mir-577 | 3.925234 | 3.897161 | 111.6197 | 4.33E-26 | 6.87E-25 |
| hsa-mir-598 | -1.18697 | 5.141323 | 38.71362 | 4.91E-10 | 2.14E-09 |
| hsa-mir-615 | 4.065774 | 2.603611 | 156.7002 | 5.95E-36 | 1.97E-34 |
| hsa-mir-616 | 1.752155 | 2.246104 | 55.00061 | 1.20E-13 | 7.36E-13 |
| hsa-mir-629 | 1.02538 | 7.429759 | 80.08073 | 3.59E-19 | 3.86E-18 |
| hsa-mir-651 | 1.157499 | 3.136145 | 25.75783 | 3.87E-07 | 1.19E-06 |
| hsa-mir-660 | -1.03642 | 6.373263 | 51.97234 | 5.63E-13 | 3.06E-12 |
| hsa-mir-671 | 1.318031 | 3.464159 | 87.64263 | 7.84E-21 | 1.00E-19 |
| hsa-mir-675 | 1.213102 | 6.278822 | 8.988503 | 0.002717 | 0.00542 |
| hsa-mir-708 | 2.778247 | 7.83498 | 149.7872 | 1.93E-34 | 5.11E-33 |
| hsa-mir-7-1 | 1.086067 | 4.532772 | 56.13505 | 6.77E-14 | 4.20E-13 |
| hsa-mir-744 | 1.259283 | 5.744702 | 77.80065 | 1.14E-18 | 1.13E-17 |
| hsa-mir-760 | 2.611377 | 2.310492 | 103.9617 | 2.06E-24 | 3.15E-23 |
| hsa-mir-767 | 5.161617 | 4.646168 | 84.92002 | 3.11E-20 | 3.63E-19 |
| hsa-mir-877 | 1.965679 | 1.980332 | 54.29318 | 1.73E-13 | 1.02E-12 |
| hsa-mir-891a | 1.826464 | 5.117381 | 17.96656 | 2.25E-05 | 5.72E-05 |
| hsa-mir-9-1 | 4.705761 | 10.93797 | 61.89457 | 3.62E-15 | 2.52E-14 |
| hsa-mir-9-2 | 4.679273 | 10.93391 | 61.55984 | 4.29E-15 | 2.94E-14 |
| hsa-mir-9-3 | 2.88854 | 1.802997 | 37.32921 | 9.98E-10 | 4.26E-09 |
| hsa-mir-937 | 2.045877 | 2.483959 | 73.70743 | 9.06E-18 | 8.56E-17 |
| hsa-mir-939 | 1.296444 | 1.645415 | 22.47356 | 2.13E-06 | 6.36E-06 |
| hsa-mir-940 | 1.843486 | 2.521089 | 64.11445 | 1.17E-15 | 9.51E-15 |
| hsa-mir-944 | 5.378694 | 6.198007 | 202.8864 | 4.90E-46 | 3.24E-44 |
| hsa-mir-96 | 2.103932 | 4.169962 | 78.42938 | 8.29E-19 | 8.44E-18 |
| hsa-mir-99a | -1.59794 | 9.426791 | 17.90047 | 2.33E-05 | 5.89E-05 |

*FC: fold change; CPM: Counts Per Million; LR: likehood Raito

**Supplementary Table S3. The over-representation analysis for target genes**

| **Category** | **Subcategory** | **expected** | **observed** | **p-value**  (fdr) |
| --- | --- | --- | --- | --- |
| KEGG | [visit](http://www.genome.jp/dbget-bin/show_pathway?hsa04120+8945+867+868+996+8453+8452+8450+8065+1161+23291+55294+51343+8916+26091+55958+23295+4281+4734+378884+5371+9886+23221+6477+6500+22954+4591+55236+7319+7321+7322+7323+10477+140739+7326+3093+7332+7334+63893+55585+92912+54926+55284+7337+9690+11060) Ubiquitin mediated proteolysis | 23.7367 | 45 | 0.000223966 |
| KEGG | [visit](http://www.genome.jp/dbget-bin/show_pathway?hsa00190+518+245973+1353+1349+4718+4719) Oxidative phosphorylation | 22.8829 | 6 | 0.000225224 |
| KEGG | [visit](http://www.genome.jp/dbget-bin/show_pathway?hsa04520+52+88+998+1387+1495+1499+2033+3480+3643+8826+4008+5594+4233+51701+5792+5879+4087+4088+6591+117178+83439+7046+7048+7082+7414+10163+8976+7525) Adherens junction | 12.8076 | 28 | 0.000514205 |
| KEGG | [visit](http://www.genome.jp/dbget-bin/show_pathway?hsa04720+107+775+1387+2033+2776+2891+2902+2911+2915+3708+3709+3845+5594+4893+23236+5332+5500+4659+5532+5534+5567+5568+5578+5613+5908+6195) Long-term potentiation | 11.9537 | 26 | 0.00078738 |
| KEGG | [visit](http://www.genome.jp/dbget-bin/show_pathway?hsa05211+9915+998+1387+8453+54583+112398+112399+2033+2034+2113+2549+2885+3091+3725+3845+5594+4233+4893+5062+57144+5295+5781+5879+5908+7039+7422) Renal cell carcinoma | 11.9537 | 26 | 0.00078738 |
| KEGG | [visit](http://www.genome.jp/dbget-bin/show_pathway?hsa04350+90+93+353500+659+1030+1387+2033+392255+3400+5594+5308+5515+5516+5934+6093+9475+6198+6500+4086+4087+4088+4090+4091+6667+7046+7048+7057+7058+7059+9372) TGF-beta signaling pathway | 14.5153 | 30 | 0.00078738 |
| KEGG | [visit](http://www.genome.jp/dbget-bin/show_pathway?hsa01100+60496+33+36+2182+55902+128+56895+10768+23382+210+216+220+144245+440138+85365+79796+262+427+445+518+245973+8706+10317+10678+84002+8704+8702+9331+586+587+56898+10380+847+1120+79586+1152+1353+1349+55454+56474+1544+1573+113612+1582+1629+9162+1609+1718+1719+1737+1788+5169+55500+55224+2235+2530+10690+2581+2585+2590+2591+8693+51809+2628+2730+2650+9615+2686+2710+2744+2746+29925+132789+57678+2821+9563+138050+3099+3156+3157+3290+51144+3417+3418+3423+3633+3705+2531+3939+160287+3945+9388+79888+4047+4121+4144+8972+11320+11343+326625+8898+9107+9108+4548+4669+4718+4719+29922+23057+4952+5048+53354+79646+5091+80055+55361+10026+23556+54965+5287+23396+23236+5332+5335+5337+84172+246721+10621+10622+5980+6240+50484+6309+6342+259230+8879+9197+55512+6487+30815+8867+6898+1787+7299+7498+9942+64131+64132) Metabolic pathways | 191.26 | 148 | 0.00080346 |
| KEGG | [visit](http://www.genome.jp/dbget-bin/show_pathway?hsa04510+88+394+596+824+858+859+894+998+1301+1286+1289+1499+1793+2002+2335+2885+3479+3480+3673+3678+3685+3688+3690+3696+3725+3915+5594+5601+4233+91807+5062+57144+55742+80310+5156+5170+5295+23396+5500+4659+5578+5829+5879+5908+6093+9475+6464+399694+7057+7058+7059+7094+7410+7414+7422) Focal adhesion | 34.3243 | 55 | 0.00191901 |
| KEGG | [visit](http://www.genome.jp/dbget-bin/show_pathway?hsa04971+107+108+112+482+483+1080+7430+2770+2773+2776+3708+3709+3772+3759+54207+3776+3784+91807+23236+5332+5567+5568+5578+5613+115111+389015) Gastric acid secretion | 12.6368 | 26 | 0.00191901 |
| KEGG | [visit](http://www.genome.jp/dbget-bin/show_pathway?hsa04810+10152+88+324+23365+8874+998+1072+1073+1730+1793+55740+7430+8817+2247+8074+2254+2263+2335+8826+3673+3678+3685+3687+3688+3690+3696+3845+5594+22808+4478+4628+91807+10787+4893+5062+57144+80310+5156+5216+5217+5295+79837+23396+5500+4659+5829+5879+5962+6093+9475+22800+85464+7114+7117+7410+7414+10163+8976) Regulation of actin cytoskeleton | 36.8858 | 58 | 0.0020636 |
| KEGG | [visit](http://www.genome.jp/dbget-bin/show_pathway?hsa04144+153+157+382+84364+858+859+867+868+998+25978+51510+7852+10059+8411+30844+22905+2066+89853+2263+9815+3480+4233+4734+84612+10015+5156+23396+5337+5371+5584+23362+8766+22841+84440+57403+5867+5868+7879+9135+5979+4087+4088+4091+60682+10254+7037+7046+7048+9101+51652+51028+79720+9525+64145+9372) Endocytosis | 35.0074 | 55 | 0.0029082 |
| KEGG | [visit](http://www.genome.jp/dbget-bin/show_pathway?hsa05100+858+859+867+868+23607+998+1495+1499+2017+10059+1793+9844+2335+2549+3678+3688+4233+5295+5829+5879+6464+399694+7414+10163+8976) Bacterial invasion of epithelial cells | 12.466 | 25 | 0.00309626 |
| KEGG | [visit](http://www.genome.jp/dbget-bin/show_pathway?hsa05322+88+729+942+94239+3020+8349+440689+3111+3127+6738) Systemic lupus erythematosus | 24.0783 | 10 | 0.0042196 |
| KEGG | [visit](http://www.genome.jp/dbget-bin/show_pathway?hsa05216+8030+1499+3845+5594+8031+4893+7849+5979+6256+6257+83439+7170+7175) Thyroid cancer | 4.95227 | 13 | 0.00455441 |
| KEGG | [visit](http://www.genome.jp/dbget-bin/show_pathway?hsa04916+107+108+112+1385+1387+1499+1910+2033+7976+8322+7855+8323+2770+2773+2775+2776+4254+3845+5594+4286+4893+23236+5332+5567+5568+5578+5613+83439+7299+7471+7472) Melanogenesis | 17.4183 | 31 | 0.00572629 |
| KEGG | [visit](http://www.genome.jp/dbget-bin/show_pathway?hsa04540+107+108+112+153+1812+1813+2697+2770+2773+2776+2885+2911+2915+3358+3708+3709+3845+5594+4893+80310+5156+23236+5332+5567+5568+5578+5613+7082) Gap junction | 15.3691 | 28 | 0.00655634 |
| KEGG | [visit](http://www.genome.jp/dbget-bin/show_pathway?hsa05131+8945+960+998+2017+1793+9844+23291+3576+3678+3688+5594+5601+5216+5217+5879+6093+9475+7322+7414+10163+8976) Shigellosis | 10.9291 | 21 | 0.0133934 |
| KEGG | [visit](http://www.genome.jp/dbget-bin/show_pathway?hsa04010+627+775+777+781+9254+93589+782+783+785+998+1843+11221+1846+2002+355+8817+2247+8074+2254+2263+2353+2885+3554+3725+3845+5606+4215+4216+9448+5594+9479+23162+5601+8550+4149+4208+22808+4763+4776+51701+4893+4915+5062+5156+5495+5532+5534+5567+5568+5578+5613+5879+5908+5921+25780+6195+22800+6722+6789+3925+57551+7046+7048+51776) MAPK signaling pathway | 45.595 | 64 | 0.0168977 |
| KEGG | [visit](http://www.genome.jp/dbget-bin/show_pathway?hsa04910+867+868+2002+10211+2308+2885+2997+3099+3643+3667+3845+5594+5601+4893+5139+5140+5170+5255+5257+5295+10891+5500+5506+5562+5567+5568+51422+5573+5584+5613+5792+23433+6198+6464+399694+8835+122809) Insulin signaling pathway | 23.3952 | 37 | 0.0169985 |
| KEGG | [visit](http://www.genome.jp/dbget-bin/show_pathway?hsa05414+107+108+112+153+488+775+781+9254+93589+782+783+785+1605+1756+3479+3673+3678+3685+3688+3690+3696+5567+5568+5613+6443+6444+7170) Dilated cardiomyopathy | 15.7106 | 27 | 0.0170073 |
| KEGG | [visit](http://www.genome.jp/dbget-bin/show_pathway?hsa04730+1394+2770+2773+2775+2776+2891+2892+2911+2915+3479+3480+3708+3709+3845+4067+5594+4893+23236+5332+5515+5516+5578) Long-term depression | 11.9537 | 22 | 0.0170073 |
| KEGG | [visit](http://www.genome.jp/dbget-bin/show_pathway?hsa05210+324+26060+8313+596+332+1499+54205+2353+3725+3845+5594+5601+4437+5295+5879+4087+4088+83439+7046+7048) Colorectal cancer | 10.5876 | 20 | 0.01748 |
| KEGG | [visit](http://www.genome.jp/dbget-bin/show_pathway?hsa05012+518+1349+54205+2861+120892+4718+4719+6622+7326+7332+7416) Parkinson’s disease | 22.5414 | 11 | 0.0190941 |
| KEGG | [visit](http://www.genome.jp/dbget-bin/show_pathway?hsa00982+128+220+1544+2327) Drug metabolism - cytochrome P450 | 12.466 | 4 | 0.0191497 |
| KEGG | [visit](http://www.genome.jp/dbget-bin/show_pathway?hsa05412+88+488+775+781+9254+93589+782+783+785+1495+1499+1605+1756+2697+3673+3678+3685+3688+3690+3696+6443+6444+83439) Arrhythmogenic right ventricular cardiomyopathy (ARVC) | 12.9784 | 23 | 0.0196125 |
| KEGG | [visit](http://www.genome.jp/dbget-bin/show_pathway?hsa00980+128+1646+220+1544) Metabolism of xenobiotics by cytochrome P450 | 12.1245 | 4 | 0.0235031 |
| KEGG | [visit](http://www.genome.jp/dbget-bin/show_pathway?hsa04912+107+108+112+775+998+2002+2488+2776+2798+2885+1839+3708+3709+3725+3845+5606+4215+4216+5594+5601+4893+23236+5332+5337+5567+5568+5578+5613) GnRH signaling pathway | 17.2475 | 28 | 0.0282801 |
| KEGG | [visit](http://www.genome.jp/dbget-bin/show_pathway?hsa04320+64506+2113+2114+2120+2885+3845+5594+4851+4853+56907) Dorso-ventral axis formation | 4.2692 | 10 | 0.0321559 |
| KEGG | [visit](http://www.genome.jp/dbget-bin/show_pathway?hsa05223+1021+1871+2309+2885+3845+5594+4893+5170+5295+5335+5578+5915+11186+6256+6257+6789+7039) Non-small cell lung cancer | 9.22146 | 17 | 0.0372979 |
| KEGG | [visit](http://www.genome.jp/dbget-bin/show_pathway?hsa03050+23198+9491) Proteasome | 8.19685 | 2 | 0.0388988 |
| KEGG | [visit](http://www.genome.jp/dbget-bin/show_pathway?hsa04114+107+108+112+8945+996+1017+64506+23291+3479+3480+3708+3709+5594+5500+5515+5516+5526+5527+5529+5532+5534+5567+5568+5613+6195+6500+9748+8243+7532+10971) Oocyte meiosis | 19.4675 | 30 | 0.0421211 |
| KEGG | [visit](http://www.genome.jp/dbget-bin/show_pathway?hsa04060+90+93+659+6355+10803+7852+1896+10913+355+2690+3459+3587+3596+3554+3556+53832+50615+163702+3574+3576+4254+3977+4233+5156+5618+7046+7048+55504+8600+8740+7422) Cytokine-cytokine receptor interaction | 45.2535 | 31 | 0.0422467 |
| KEGG | [visit](http://www.genome.jp/dbget-bin/show_pathway?hsa04623+10621+10622+8737) Cytosolic DNA-sensing pathway | 9.563 | 3 | 0.0422467 |
| KEGG | [visit](http://www.genome.jp/dbget-bin/show_pathway?hsa00830+128+216+1544+9227) Retinol metabolism | 11.0999 | 4 | 0.0422467 |
| KEGG | [visit](http://www.genome.jp/dbget-bin/show_pathway?hsa04340+353500+8945+1454+53944+1456+23291+2736+2737+4036+5567+5568+5613+5727+51715+7471+7472+7546) Hedgehog signaling pathway | 9.563 | 17 | 0.0468588 |
| Gene Ontology | [visit](http://www.godatabase.org/cgi-bin/amigo/go.cgi?view=details&search_constraint=terms&depth=0&query=GO:0005515) protein binding | 1403.6 | 1704 | 2.20661e-29 |
| Gene Ontology | [visit](http://www.godatabase.org/cgi-bin/amigo/go.cgi?view=details&search_constraint=terms&depth=0&query=GO:0005488) binding | 2133.83 | 2401 | 4.83996e-29 |
| Gene Ontology | [visit](http://www.godatabase.org/cgi-bin/amigo/go.cgi?view=details&search_constraint=terms&depth=0&query=GO:0009987) cellular process | 2002.53 | 2279 | 7.2477e-29 |
| Gene Ontology | [visit](http://www.godatabase.org/cgi-bin/amigo/go.cgi?view=details&search_constraint=terms&depth=0&query=GO:0007606) sensory perception of chemical stimulus | 82.3619 | 6 | 3.14526e-28 |
| Gene Ontology | [visit](http://www.godatabase.org/cgi-bin/amigo/go.cgi?view=details&search_constraint=terms&depth=0&query=GO:0050794) regulation of cellular process | 1076.91 | 1349 | 3.54522e-26 |
| Gene Ontology | [visit](http://www.godatabase.org/cgi-bin/amigo/go.cgi?view=details&search_constraint=terms&depth=0&query=GO:0007608) sensory perception of smell | 74.4359 | 5 | 5.62988e-26 |
| Gene Ontology | [visit](http://www.godatabase.org/cgi-bin/amigo/go.cgi?view=details&search_constraint=terms&depth=0&query=GO:0065007) biological regulation | 1201.14 | 1475 | 1.07357e-25 |
| Gene Ontology | [visit](http://www.godatabase.org/cgi-bin/amigo/go.cgi?view=details&search_constraint=terms&depth=0&query=GO:0050789) regulation of biological process | 1133.08 | 1401 | 4.44066e-25 |
| Gene Ontology | [visit](http://www.godatabase.org/cgi-bin/amigo/go.cgi?view=details&search_constraint=terms&depth=0&query=GO:0032502) developmental process | 589.629 | 806 | 1.67278e-23 |
| Gene Ontology | [visit](http://www.godatabase.org/cgi-bin/amigo/go.cgi?view=details&search_constraint=terms&depth=0&query=GO:0007275) multicellular organismal development | 537.42 | 740 | 4.87161e-22 |
| Gene Ontology | [visit](http://www.godatabase.org/cgi-bin/amigo/go.cgi?view=details&search_constraint=terms&depth=0&query=GO:0023052) signaling | 615.302 | 817 | 7.2664e-20 |
| Gene Ontology | [visit](http://www.godatabase.org/cgi-bin/amigo/go.cgi?view=details&search_constraint=terms&depth=0&query=GO:0048731) system development | 443.341 | 621 | 1.24083e-19 |
| Gene Ontology | [visit](http://www.godatabase.org/cgi-bin/amigo/go.cgi?view=details&search_constraint=terms&depth=0&query=GO:0048856) anatomical structure development | 491.587 | 674 | 3.64331e-19 |
| Gene Ontology | [visit](http://www.godatabase.org/cgi-bin/amigo/go.cgi?view=details&search_constraint=terms&depth=0&query=GO:0060255) regulation of macromolecule metabolic process | 587.733 | 774 | 1.48789e-17 |
| Gene Ontology | [visit](http://www.godatabase.org/cgi-bin/amigo/go.cgi?view=details&search_constraint=terms&depth=0&query=GO:0007399) nervous system development | 202.286 | 323 | 2.14201e-17 |
| Gene Ontology | [visit](http://www.godatabase.org/cgi-bin/amigo/go.cgi?view=details&search_constraint=terms&depth=0&query=GO:0019222) regulation of metabolic process | 680.778 | 875 | 2.58448e-17 |
| Gene Ontology | [visit](http://www.godatabase.org/cgi-bin/amigo/go.cgi?view=details&search_constraint=terms&depth=0&query=GO:0080090) regulation of primary metabolic process | 617.714 | 801 | 1.77332e-16 |
| Gene Ontology | [visit](http://www.godatabase.org/cgi-bin/amigo/go.cgi?view=details&search_constraint=terms&depth=0&query=GO:0023033) signaling pathway | 438.862 | 597 | 8.35687e-16 |
| Gene Ontology | [visit](http://www.godatabase.org/cgi-bin/amigo/go.cgi?view=details&search_constraint=terms&depth=0&query=GO:0051179) localization | 579.29 | 754 | 1.06934e-15 |
| Gene Ontology | [visit](http://www.godatabase.org/cgi-bin/amigo/go.cgi?view=details&search_constraint=terms&depth=0&query=GO:0031323) regulation of cellular metabolic process | 649.246 | 828 | 3.42796e-15 |
| Gene Ontology | [visit](http://www.godatabase.org/cgi-bin/amigo/go.cgi?view=details&search_constraint=terms&depth=0&query=GO:0004930) G-protein coupled receptor activity | 147.321 | 64 | 4.36004e-15 |
| Gene Ontology | [visit](http://www.godatabase.org/cgi-bin/amigo/go.cgi?view=details&search_constraint=terms&depth=0&query=GO:0010468) regulation of gene expression | 509.679 | 670 | 1.46831e-14 |
| Gene Ontology | [visit](http://www.godatabase.org/cgi-bin/amigo/go.cgi?view=details&search_constraint=terms&depth=0&query=GO:0009889) regulation of biosynthetic process | 528.977 | 690 | 2.65451e-14 |
| Gene Ontology | [visit](http://www.godatabase.org/cgi-bin/amigo/go.cgi?view=details&search_constraint=terms&depth=0&query=GO:0031326) regulation of cellular biosynthetic process | 525.186 | 685 | 3.51786e-14 |
| Gene Ontology | [visit](http://www.godatabase.org/cgi-bin/amigo/go.cgi?view=details&search_constraint=terms&depth=0&query=GO:0010556) regulation of macromolecule biosynthetic process | 499.858 | 656 | 4.62323e-14 |
| Gene Ontology | [visit](http://www.godatabase.org/cgi-bin/amigo/go.cgi?view=details&search_constraint=terms&depth=0&query=GO:0019219) regulation of nucleobase, nucleoside, nucleotide and nucleic acid metabolic process | 523.808 | 681 | 8.95717e-14 |
| Gene Ontology | [visit](http://www.godatabase.org/cgi-bin/amigo/go.cgi?view=details&search_constraint=terms&depth=0&query=GO:0051171) regulation of nitrogen compound metabolic process | 528.288 | 684 | 1.92111e-13 |
| Gene Ontology | [visit](http://www.godatabase.org/cgi-bin/amigo/go.cgi?view=details&search_constraint=terms&depth=0&query=GO:0023046) signaling process | 444.031 | 588 | 3.9671e-13 |
| Gene Ontology | [visit](http://www.godatabase.org/cgi-bin/amigo/go.cgi?view=details&search_constraint=terms&depth=0&query=GO:0023060) signal transmission | 442.997 | 585 | 8.30903e-13 |
| Gene Ontology | [visit](http://www.godatabase.org/cgi-bin/amigo/go.cgi?view=details&search_constraint=terms&depth=0&query=GO:0016337) cell-cell adhesion | 54.7931 | 112 | 9.67322e-13 |
| Gene Ontology | [visit](http://www.godatabase.org/cgi-bin/amigo/go.cgi?view=details&search_constraint=terms&depth=0&query=GO:0007600) sensory perception | 143.013 | 68 | 2.12928e-12 |
| Gene Ontology | [visit](http://www.godatabase.org/cgi-bin/amigo/go.cgi?view=details&search_constraint=terms&depth=0&query=GO:0016043) cellular component organization | 483.833 | 628 | 2.27812e-12 |
| Gene Ontology | [visit](http://www.godatabase.org/cgi-bin/amigo/go.cgi?view=details&search_constraint=terms&depth=0&query=GO:0044459) plasma membrane part | 348.574 | 475 | 2.37386e-12 |
| Gene Ontology | [visit](http://www.godatabase.org/cgi-bin/amigo/go.cgi?view=details&search_constraint=terms&depth=0&query=GO:0005622) intracellular | 1952.05 | 2132 | 5.63011e-12 |
| Gene Ontology | [visit](http://www.godatabase.org/cgi-bin/amigo/go.cgi?view=details&search_constraint=terms&depth=0&query=GO:0044260) cellular macromolecule metabolic process | 981.968 | 1159 | 8.26097e-12 |
| Gene Ontology | [visit](http://www.godatabase.org/cgi-bin/amigo/go.cgi?view=details&search_constraint=terms&depth=0&query=GO:0048513) organ development | 333.066 | 453 | 1.42379e-11 |
| Gene Ontology | [visit](http://www.godatabase.org/cgi-bin/amigo/go.cgi?view=details&search_constraint=terms&depth=0&query=GO:0045449) regulation of transcription | 456.092 | 591 | 2.19309e-11 |
| Gene Ontology | [visit](http://www.godatabase.org/cgi-bin/amigo/go.cgi?view=details&search_constraint=terms&depth=0&query=GO:0009653) anatomical structure morphogenesis | 240.538 | 344 | 2.33669e-11 |
| Gene Ontology | [visit](http://www.godatabase.org/cgi-bin/amigo/go.cgi?view=details&search_constraint=terms&depth=0&query=GO:0007156) homophilic cell adhesion | 23.9504 | 61 | 2.38179e-11 |
| Gene Ontology | [visit](http://www.godatabase.org/cgi-bin/amigo/go.cgi?view=details&search_constraint=terms&depth=0&query=GO:0006350) transcription | 472.806 | 606 | 8.0533e-11 |
| Gene Ontology | [visit](http://www.godatabase.org/cgi-bin/amigo/go.cgi?view=details&search_constraint=terms&depth=0&query=GO:0030528) transcription regulator activity | 262.421 | 367 | 8.35172e-11 |
| Gene Ontology | [visit](http://www.godatabase.org/cgi-bin/amigo/go.cgi?view=details&search_constraint=terms&depth=0&query=GO:0048522) positive regulation of cellular process | 351.158 | 469 | 9.29828e-11 |
| Gene Ontology | [visit](http://www.godatabase.org/cgi-bin/amigo/go.cgi?view=details&search_constraint=terms&depth=0&query=GO:0048523) negative regulation of cellular process | 324.968 | 437 | 2.24417e-10 |
| Gene Ontology | [visit](http://www.godatabase.org/cgi-bin/amigo/go.cgi?view=details&search_constraint=terms&depth=0&query=GO:0044424) intracellular part | 1891.22 | 2060 | 2.58444e-10 |
| Gene Ontology | [visit](http://www.godatabase.org/cgi-bin/amigo/go.cgi?view=details&search_constraint=terms&depth=0&query=GO:0051234) establishment of localization | 501.408 | 633 | 3.8615e-10 |
| Gene Ontology | [visit](http://www.godatabase.org/cgi-bin/amigo/go.cgi?view=details&search_constraint=terms&depth=0&query=GO:0045935) positive regulation of nucleobase, nucleoside, nucleotide and nucleic acid metabolic process | 117.168 | 188 | 4.09338e-10 |
| Gene Ontology | [visit](http://www.godatabase.org/cgi-bin/amigo/go.cgi?view=details&search_constraint=terms&depth=0&query=GO:0048869) cellular developmental process | 331.515 | 442 | 5.82074e-10 |
| Gene Ontology | [visit](http://www.godatabase.org/cgi-bin/amigo/go.cgi?view=details&search_constraint=terms&depth=0&query=GO:0007154) cell communication | 290.679 | 395 | 6.34903e-10 |
| Gene Ontology | [visit](http://www.godatabase.org/cgi-bin/amigo/go.cgi?view=details&search_constraint=terms&depth=0&query=GO:0030154) cell differentiation | 316.008 | 424 | 6.36822e-10 |
| Gene Ontology | [visit](http://www.godatabase.org/cgi-bin/amigo/go.cgi?view=details&search_constraint=terms&depth=0&query=GO:0006810) transport | 495.205 | 624 | 7.56503e-10 |
| Gene Ontology | [visit](http://www.godatabase.org/cgi-bin/amigo/go.cgi?view=details&search_constraint=terms&depth=0&query=GO:0043231) intracellular membranebounded organelle | 1443.23 | 1613 | 9.02415e-10 |
| Gene Ontology | [visit](http://www.godatabase.org/cgi-bin/amigo/go.cgi?view=details&search_constraint=terms&depth=0&query=GO:0043227) membrane-bounded organelle | 1444.44 | 1614 | 9.37266e-10 |
| Gene Ontology | [visit](http://www.godatabase.org/cgi-bin/amigo/go.cgi?view=details&search_constraint=terms&depth=0&query=GO:0051173) positive regulation of nitrogen compound metabolic process | 120.786 | 191 | 9.69225e-10 |
| Gene Ontology | [visit](http://www.godatabase.org/cgi-bin/amigo/go.cgi?view=details&search_constraint=terms&depth=0&query=GO:0008150) biological process | 2467.07 | 2598 | 1.1118e-09 |
| Gene Ontology | [visit](http://www.godatabase.org/cgi-bin/amigo/go.cgi?view=details&search_constraint=terms&depth=0&query=GO:0010604) positive regulation of macromolecule metabolic  process | 168.17 | 249 | 1.23531e-09 |
| Gene Ontology | [visit](http://www.godatabase.org/cgi-bin/amigo/go.cgi?view=details&search_constraint=terms&depth=0&query=GO:0003674) molecular function | 2664.18 | 2774 | 1.36637e-09 |
| Gene Ontology | [visit](http://www.godatabase.org/cgi-bin/amigo/go.cgi?view=details&search_constraint=terms&depth=0&query=GO:0003700) transcription factor activity | 166.102 | 246 | 1.55626e-09 |
| Gene Ontology | [visit](http://www.godatabase.org/cgi-bin/amigo/go.cgi?view=details&search_constraint=terms&depth=0&query=GO:0007155) cell adhesion | 142.496 | 217 | 1.65918e-09 |
| Gene Ontology | [visit](http://www.godatabase.org/cgi-bin/amigo/go.cgi?view=details&search_constraint=terms&depth=0&query=GO:0022610) biological adhesion | 142.669 | 217 | 1.84314e-09 |
| Gene Ontology | [visit](http://www.godatabase.org/cgi-bin/amigo/go.cgi?view=details&search_constraint=terms&depth=0&query=GO:0007166) cell surface receptor linked signaling pathway | 257.941 | 354 | 2.06478e-09 |
| Gene Ontology | [visit](http://www.godatabase.org/cgi-bin/amigo/go.cgi?view=details&search_constraint=terms&depth=0&query=GO:0044464) cell part | 2595.95 | 2712 | 2.41343e-09 |
| Gene Ontology | [visit](http://www.godatabase.org/cgi-bin/amigo/go.cgi?view=details&search_constraint=terms&depth=0&query=GO:0005623) cell | 2596.12 | 2712 | 2.51067e-09 |
| Gene Ontology | [visit](http://www.godatabase.org/cgi-bin/amigo/go.cgi?view=details&search_constraint=terms&depth=0&query=GO:0048518) positive regulation of biological process | 385.964 | 499 | 2.51067e-09 |
| Gene Ontology | [visit](http://www.godatabase.org/cgi-bin/amigo/go.cgi?view=details&search_constraint=terms&depth=0&query=GO:0009059) macromolecule biosynthetic process | 619.782 | 755 | 2.64639e-09 |
| Gene Ontology | [visit](http://www.godatabase.org/cgi-bin/amigo/go.cgi?view=details&search_constraint=terms&depth=0&query=GO:0007167) enzyme linked receptor protein signaling pathway | 81.845 | 139 | 2.92198e-09 |
| Gene Ontology | [visit](http://www.godatabase.org/cgi-bin/amigo/go.cgi?view=details&search_constraint=terms&depth=0&query=GO:0034645) cellular macromolecule biosynthetic process | 608.065 | 742 | 2.92198e-09 |
| Gene Ontology | [visit](http://www.godatabase.org/cgi-bin/amigo/go.cgi?view=details&search_constraint=terms&depth=0&query=GO:0043170) macromolecule metabolic process | 1081.04 | 1239 | 3.32814e-09 |
| Gene Ontology | [visit](http://www.godatabase.org/cgi-bin/amigo/go.cgi?view=details&search_constraint=terms&depth=0&query=GO:0007165) signal transduction | 384.585 | 496 | 4.02968e-09 |
| Gene Ontology | [visit](http://www.godatabase.org/cgi-bin/amigo/go.cgi?view=details&search_constraint=terms&depth=0&query=GO:0051254) positive regulation of RNA metabolic process | 89.9434 | 149 | 4.16504e-09 |
| Gene Ontology | [visit](http://www.godatabase.org/cgi-bin/amigo/go.cgi?view=details&search_constraint=terms&depth=0&query=GO:0023034) intracellular signaling pathway | 262.249 | 357 | 4.19168e-09 |
| Gene Ontology | [visit](http://www.godatabase.org/cgi-bin/amigo/go.cgi?view=details&search_constraint=terms&depth=0&query=GO:0045941) positive regulation of transcription | 103.211 | 166 | 4.19168e-09 |
| Gene Ontology | [visit](http://www.godatabase.org/cgi-bin/amigo/go.cgi?view=details&search_constraint=terms&depth=0&query=GO:0046872) metal ion binding | 652.348 | 787 | 5.95067e-09 |
| Gene Ontology | [visit](http://www.godatabase.org/cgi-bin/amigo/go.cgi?view=details&search_constraint=terms&depth=0&query=GO:0043005) neuron projection | 63.9253 | 114 | 6.11252e-09 |
| Gene Ontology | [visit](http://www.godatabase.org/cgi-bin/amigo/go.cgi?view=details&search_constraint=terms&depth=0&query=GO:0045893) positive regulation of transcription, DNA-dependent | 88.9095 | 147 | 6.11252e-09 |
| Gene Ontology | [visit](http://www.godatabase.org/cgi-bin/amigo/go.cgi?view=details&search_constraint=terms&depth=0&query=GO:0009893) positive regulation of metabolic process | 181.265 | 261 | 6.42683e-09 |
| Gene Ontology | [visit](http://www.godatabase.org/cgi-bin/amigo/go.cgi?view=details&search_constraint=terms&depth=0&query=GO:0010628) positive regulation of gene expression | 108.897 | 172 | 8.31374e-09 |
| Gene Ontology | [visit](http://www.godatabase.org/cgi-bin/amigo/go.cgi?view=details&search_constraint=terms&depth=0&query=GO:0048519) negative regulation of biological process | 355.638 | 461 | 9.42754e-09 |
| Gene Ontology | [visit](http://www.godatabase.org/cgi-bin/amigo/go.cgi?view=details&search_constraint=terms&depth=0&query=GO:0008092) cytoskeletal protein binding | 88.2203 | 145 | 1.2333e-08 |
| Gene Ontology | [visit](http://www.godatabase.org/cgi-bin/amigo/go.cgi?view=details&search_constraint=terms&depth=0&query=GO:0042995) cell projection | 131.986 | 200 | 1.24022e-08 |
| Gene Ontology | [visit](http://www.godatabase.org/cgi-bin/amigo/go.cgi?view=details&search_constraint=terms&depth=0&query=GO:0033036) macromolecule localization | 216.415 | 301 | 1.26176e-08 |
| Gene Ontology | [visit](http://www.godatabase.org/cgi-bin/amigo/go.cgi?view=details&search_constraint=terms&depth=0&query=GO:0006357) regulation of transcription from RNA polymerase II promoter | 130.952 | 198 | 1.82741e-08 |
| Gene Ontology | [visit](http://www.godatabase.org/cgi-bin/amigo/go.cgi?view=details&search_constraint=terms&depth=0&query=GO:0043169) cation binding | 659.24 | 790 | 1.95215e-08 |
| Gene Ontology | [visit](http://www.godatabase.org/cgi-bin/amigo/go.cgi?view=details&search_constraint=terms&depth=0&query=GO:0051252) regulation of RNA  metabolic process | 322.039 | 421 | 2.02247e-08 |
| Gene Ontology | [visit](http://www.godatabase.org/cgi-bin/amigo/go.cgi?view=details&search_constraint=terms&depth=0&query=GO:0043687) post-translational protein modification | 260.009 | 350 | 2.34961e-08 |
| Gene Ontology | [visit](http://www.godatabase.org/cgi-bin/amigo/go.cgi?view=details&search_constraint=terms&depth=0&query=GO:0043167) ion binding | 660.963 | 791 | 2.41361e-08 |
| Gene Ontology | [visit](http://www.godatabase.org/cgi-bin/amigo/go.cgi?view=details&search_constraint=terms&depth=0&query=GO:0044237) cellular metabolic process | 1280.4 | 1434 | 2.53155e-08 |
| Gene Ontology | [visit](http://www.godatabase.org/cgi-bin/amigo/go.cgi?view=details&search_constraint=terms&depth=0&query=GO:0019904) protein domain specific binding | 67.716 | 117 | 2.54297e-08 |
| Gene Ontology | [visit](http://www.godatabase.org/cgi-bin/amigo/go.cgi?view=details&search_constraint=terms&depth=0&query=GO:0006366) transcription from RNA polymerase II promoter | 158.349 | 230 | 3.33648e-08 |
| Gene Ontology | [visit](http://www.godatabase.org/cgi-bin/amigo/go.cgi?view=details&search_constraint=terms&depth=0&query=GO:0048468) cell development | 138.533 | 206 | 3.36787e-08 |
| Gene Ontology | [visit](http://www.godatabase.org/cgi-bin/amigo/go.cgi?view=details&search_constraint=terms&depth=0&query=GO:0009790) embryonic development | 107.863 | 168 | 3.67707e-08 |
| Gene Ontology | [visit](http://www.godatabase.org/cgi-bin/amigo/go.cgi?view=details&search_constraint=terms&depth=0&query=GO:0031325) positive regulation of cellular metabolic process | 172.133 | 246 | 4.10619e-08 |
| Gene Ontology | [visit](http://www.godatabase.org/cgi-bin/amigo/go.cgi?view=details&search_constraint=terms&depth=0&query=GO:0006464) protein modification process | 304.636 | 399 | 4.50891e-08 |
| Gene Ontology | [visit](http://www.godatabase.org/cgi-bin/amigo/go.cgi?view=details&search_constraint=terms&depth=0&query=GO:0043229) intracellular organelle | 1610.37 | 1762 | 4.96662e-08 |
| Gene Ontology | [visit](http://www.godatabase.org/cgi-bin/amigo/go.cgi?view=details&search_constraint=terms&depth=0&query=GO:0043226) organelle | 1612.78 | 1764 | 5.41482e-08 |
| Gene Ontology | [visit](http://www.godatabase.org/cgi-bin/amigo/go.cgi?view=details&search_constraint=terms&depth=0&query=GO:0016192) vesicle-mediated transport | 115.789 | 177 | 5.79249e-08 |
| Gene Ontology | [visit](http://www.godatabase.org/cgi-bin/amigo/go.cgi?view=details&search_constraint=terms&depth=0&query=GO:0008104) protein localization | 180.576 | 255 | 6.36021e-08 |
| Gene Ontology | [visit](http://www.godatabase.org/cgi-bin/amigo/go.cgi?view=details&search_constraint=terms&depth=0&query=GO:0010557) positive regulation of macromolecule biosynthetic process | 120.958 | 183 | 7.04337e-08 |
| Gene Ontology | [visit](http://www.godatabase.org/cgi-bin/amigo/go.cgi?view=details&search_constraint=terms&depth=0&query=GO:0005737) cytoplasm | 1322.62 | 1472 | 7.20284e-08 |
| Gene Ontology | [visit](http://www.godatabase.org/cgi-bin/amigo/go.cgi?view=details&search_constraint=terms&depth=0&query=GO:0005634) nucleus | 895.643 | 1033 | 9.03484e-08 |
| Gene Ontology | [visit](http://www.godatabase.org/cgi-bin/amigo/go.cgi?view=details&search_constraint=terms&depth=0&query=GO:0050890) cognition | 159.899 | 97 | 9.83974e-08 |
| Gene Ontology | [visit](http://www.godatabase.org/cgi-bin/amigo/go.cgi?view=details&search_constraint=terms&depth=0&query=GO:0043412) macromolecule modification | 318.42 | 412 | 1.07277e-07 |
| Gene Ontology | [visit](http://www.godatabase.org/cgi-bin/amigo/go.cgi?view=details&search_constraint=terms&depth=0&query=GO:0044238) primary metabolic process | 1314.52 | 1462 | 1.07301e-07 |
| Gene Ontology | [visit](http://www.godatabase.org/cgi-bin/amigo/go.cgi?view=details&search_constraint=terms&depth=0&query=GO:0022008) neurogenesis | 111.309 | 170 | 1.2373e-07 |
| Gene Ontology | [visit](http://www.godatabase.org/cgi-bin/amigo/go.cgi?view=details&search_constraint=terms&depth=0&query=GO:0031328) positive regulation of cellular biosynthetic process | 128.54 | 191 | 1.33247e-07 |
| Gene Ontology | [visit](http://www.godatabase.org/cgi-bin/amigo/go.cgi?view=details&search_constraint=terms&depth=0&query=GO:0009891) positive regulation of biosynthetic process | 130.435 | 193 | 1.55572e-07 |
| Gene Ontology | [visit](http://www.godatabase.org/cgi-bin/amigo/go.cgi?view=details&search_constraint=terms&depth=0&query=GO:0016563) transcription activator activity | 74.0913 | 122 | 2.25823e-07 |
| Gene Ontology | [visit](http://www.godatabase.org/cgi-bin/amigo/go.cgi?view=details&search_constraint=terms&depth=0&query=GO:0009058) biosynthetic process | 764.346 | 890 | 3.53852e-07 |
| Gene Ontology | [visit](http://www.godatabase.org/cgi-bin/amigo/go.cgi?view=details&search_constraint=terms&depth=0&query=GO:0045944) positive regulation of transcription from RNA polymerase II promoter | 68.5775 | 114 | 3.96575e-07 |
| Gene Ontology | [visit](http://www.godatabase.org/cgi-bin/amigo/go.cgi?view=details&search_constraint=terms&depth=0&query=GO:0005882) intermediate filament | 30.6703 | 6 | 4.06761e-07 |
| Gene Ontology | [visit](http://www.godatabase.org/cgi-bin/amigo/go.cgi?view=details&search_constraint=terms&depth=0&query=GO:0016477) cell migration | 79.605 | 128 | 4.43854e-07 |
| Gene Ontology | [visit](http://www.godatabase.org/cgi-bin/amigo/go.cgi?view=details&search_constraint=terms&depth=0&query=GO:0010629) negative regulation of gene expression | 98.7309 | 152 | 4.61203e-07 |
| Gene Ontology | [visit](http://www.godatabase.org/cgi-bin/amigo/go.cgi?view=details&search_constraint=terms&depth=0&query=GO:0006928) cellular component movement | 112.515 | 169 | 4.6307e-07 |
| Gene Ontology | [visit](http://www.godatabase.org/cgi-bin/amigo/go.cgi?view=details&search_constraint=terms&depth=0&query=GO:0006139) nucleobase, nucleoside, nucleotide and nucleic acid  metabolic process | 739.879 | 863 | 4.63424e-07 |
| Gene Ontology | [visit](http://www.godatabase.org/cgi-bin/amigo/go.cgi?view=details&search_constraint=terms&depth=0&query=GO:0006355) regulation of transcription, DNA-dependent | 313.596 | 402 | 5.05502e-07 |
| Gene Ontology | [visit](http://www.godatabase.org/cgi-bin/amigo/go.cgi?view=details&search_constraint=terms&depth=0&query=GO:0045111) intermediate filament cytoskeleton | 32.2211 | 7 | 5.19473e-07 |
| Gene Ontology | [visit](http://www.godatabase.org/cgi-bin/amigo/go.cgi?view=details&search_constraint=terms&depth=0&query=GO:0005509) calcium ion binding | 107.863 | 163 | 5.23773e-07 |
| Gene Ontology | [visit](http://www.godatabase.org/cgi-bin/amigo/go.cgi?view=details&search_constraint=terms&depth=0&query=GO:0030054) cell junction | 92.8725 | 144 | 6.60279e-07 |
| Gene Ontology | [visit](http://www.godatabase.org/cgi-bin/amigo/go.cgi?view=details&search_constraint=terms&depth=0&query=GO:0045202) synapse | 63.9253 | 107 | 6.96504e-07 |
| Gene Ontology | [visit](http://www.godatabase.org/cgi-bin/amigo/go.cgi?view=details&search_constraint=terms&depth=0&query=GO:0010558) negative regulation of macromolecule biosynthetic process | 105.279 | 159 | 8.08009e-07 |
| Gene Ontology | [visit](http://www.godatabase.org/cgi-bin/amigo/go.cgi?view=details&search_constraint=terms&depth=0&query=GO:0005576) extracellular region | 350.814 | 265 | 9.19462e-07 |
| Gene Ontology | [visit](http://www.godatabase.org/cgi-bin/amigo/go.cgi?view=details&search_constraint=terms&depth=0&query=GO:0035556) intracellular signal transduction | 207.628 | 280 | 9.42829e-07 |
| Gene Ontology | [visit](http://www.godatabase.org/cgi-bin/amigo/go.cgi?view=details&search_constraint=terms&depth=0&query=GO:0032501) multicellular organismal process | 797.601 | 920 | 1.08078e-06 |
| Gene Ontology | [visit](http://www.godatabase.org/cgi-bin/amigo/go.cgi?view=details&search_constraint=terms&depth=0&query=GO:0007420) brain development | 54.7931 | 94 | 1.36009e-06 |
| Gene Ontology | [visit](http://www.godatabase.org/cgi-bin/amigo/go.cgi?view=details&search_constraint=terms&depth=0&query=GO:0005886) plasma membrane | 647.696 | 761 | 1.38834e-06 |
| Gene Ontology | [visit](http://www.godatabase.org/cgi-bin/amigo/go.cgi?view=details&search_constraint=terms&depth=0&query=GO:0004888) transmembrane receptor  activity | 219.689 | 152 | 1.49396e-06 |
| Gene Ontology | [visit](http://www.godatabase.org/cgi-bin/amigo/go.cgi?view=details&search_constraint=terms&depth=0&query=GO:0010646) regulation of cell communication | 202.114 | 272 | 1.78819e-06 |
| Gene Ontology | [visit](http://www.godatabase.org/cgi-bin/amigo/go.cgi?view=details&search_constraint=terms&depth=0&query=GO:0044249) cellular biosynthetic process | 745.393 | 863 | 1.81379e-06 |
| Gene Ontology | [visit](http://www.godatabase.org/cgi-bin/amigo/go.cgi?view=details&search_constraint=terms&depth=0&query=GO:0051128) regulation of cellular component organization | 95.2848 | 145 | 1.93122e-06 |
| Gene Ontology | [visit](http://www.godatabase.org/cgi-bin/amigo/go.cgi?view=details&search_constraint=terms&depth=0&query=GO:0031327) negative regulation of cellular biosynthetic process | 108.38 | 161 | 2.00098e-06 |
| Gene Ontology | [visit](http://www.godatabase.org/cgi-bin/amigo/go.cgi?view=details&search_constraint=terms&depth=0&query=GO:0009890) negative regulation of biosynthetic process | 110.103 | 163 | 2.09415e-06 |
| Gene Ontology | [visit](http://www.godatabase.org/cgi-bin/amigo/go.cgi?view=details&search_constraint=terms&depth=0&query=GO:0090304) nucleic acid metabolic process | 641.665 | 752 | 2.59274e-06 |
| Gene Ontology | [visit](http://www.godatabase.org/cgi-bin/amigo/go.cgi?view=details&search_constraint=terms&depth=0&query=GO:0030030) cell projection organization | 78.9158 | 124 | 2.63649e-06 |
| Gene Ontology | [visit](http://www.godatabase.org/cgi-bin/amigo/go.cgi?view=details&search_constraint=terms&depth=0&query=GO:0045184) establishment of protein localization | 155.764 | 217 | 2.69276e-06 |
| Gene Ontology | [visit](http://www.godatabase.org/cgi-bin/amigo/go.cgi?view=details&search_constraint=terms&depth=0&query=GO:0007417) central nervous system development | 79.7773 | 125 | 2.73415e-06 |
| Gene Ontology | [visit](http://www.godatabase.org/cgi-bin/amigo/go.cgi?view=details&search_constraint=terms&depth=0&query=GO:0048666) neuron development | 68.7498 | 111 | 2.74311e-06 |
| Gene Ontology | [visit](http://www.godatabase.org/cgi-bin/amigo/go.cgi?view=details&search_constraint=terms&depth=0&query=GO:0006351) transcription, DNA dependent | 335.823 | 421 | 3.00586e-06 |
| Gene Ontology | [visit](http://www.godatabase.org/cgi-bin/amigo/go.cgi?view=details&search_constraint=terms&depth=0&query=GO:0048699) generation of neurons | 103.555 | 154 | 3.43003e-06 |
| Gene Ontology | [visit](http://www.godatabase.org/cgi-bin/amigo/go.cgi?view=details&search_constraint=terms&depth=0&query=GO:0040007) growth | 93.0448 | 141 | 3.60211e-06 |
| Gene Ontology | [visit](http://www.godatabase.org/cgi-bin/amigo/go.cgi?view=details&search_constraint=terms&depth=0&query=GO:0032774) RNA biosynthetic process | 336.512 | 421 | 3.7714e-06 |
| Gene Ontology | [visit](http://www.godatabase.org/cgi-bin/amigo/go.cgi?view=details&search_constraint=terms&depth=0&query=GO:0030182) neuron differentiation | 94.0787 | 142 | 4.141e-06 |
| Gene Ontology | [visit](http://www.godatabase.org/cgi-bin/amigo/go.cgi?view=details&search_constraint=terms&depth=0&query=GO:0000902) cell morphogenesis | 74.0913 | 117 | 4.33264e-06 |
| Gene Ontology | [visit](http://www.godatabase.org/cgi-bin/amigo/go.cgi?view=details&search_constraint=terms&depth=0&query=GO:0007169) transmembrane receptor protein tyrosine kinase signaling pathway | 51.0024 | 87 | 5.01651e-06 |
| Gene Ontology | [visit](http://www.godatabase.org/cgi-bin/amigo/go.cgi?view=details&search_constraint=terms&depth=0&query=GO:0010467) gene expression | 655.794 | 764 | 5.04313e-06 |
| Gene Ontology | [visit](http://www.godatabase.org/cgi-bin/amigo/go.cgi?view=details&search_constraint=terms&depth=0&query=GO:0032870) cellular response to hormone stimulus | 31.3596 | 60 | 6.12616e-06 |
| Gene Ontology | [visit](http://www.godatabase.org/cgi-bin/amigo/go.cgi?view=details&search_constraint=terms&depth=0&query=GO:0035466) regulation of signaling pathway | 176.096 | 238 | 8.0098e-06 |
| Gene Ontology | [visit](http://www.godatabase.org/cgi-bin/amigo/go.cgi?view=details&search_constraint=terms&depth=0&query=GO:0032879) regulation of localization | 127.678 | 181 | 9.026e-06 |
| Gene Ontology | [visit](http://www.godatabase.org/cgi-bin/amigo/go.cgi?view=details&search_constraint=terms&depth=0&query=GO:0003677) DNA binding | 404.056 | 492 | 9.12353e-06 |
| Gene Ontology | [visit](http://www.godatabase.org/cgi-bin/amigo/go.cgi?view=details&search_constraint=terms&depth=0&query=GO:0003702) RNA polymerase II transcription factor activity | 41.181 | 73 | 9.37633e-06 |
| Gene Ontology | [visit](http://www.godatabase.org/cgi-bin/amigo/go.cgi?view=details&search_constraint=terms&depth=0&query=GO:0009887) organ morphogenesis | 113.549 | 164 | 9.37633e-06 |
| Gene Ontology | [visit](http://www.godatabase.org/cgi-bin/amigo/go.cgi?view=details&search_constraint=terms&depth=0&query=GO:0071310) cellular response to organic substance | 46.5224 | 80 | 1.03809e-05 |
| Gene Ontology | [visit](http://www.godatabase.org/cgi-bin/amigo/go.cgi?view=details&search_constraint=terms&depth=0&query=GO:0016481) negative regulation of transcription | 88.2203 | 133 | 1.03991e-05 |
| Gene Ontology | [visit](http://www.godatabase.org/cgi-bin/amigo/go.cgi?view=details&search_constraint=terms&depth=0&query=GO:0008270) zinc ion binding | 346.161 | 428 | 1.07709e-05 |
| Gene Ontology | [visit](http://www.godatabase.org/cgi-bin/amigo/go.cgi?view=details&search_constraint=terms&depth=0&query=GO:0010605) negative regulation of macromolecule metabolic process | 142.496 | 198 | 1.07709e-05 |
| Gene Ontology | [visit](http://www.godatabase.org/cgi-bin/amigo/go.cgi?view=details&search_constraint=terms&depth=0&query=GO:0032553) ribonucleotide binding | 318.937 | 398 | 1.07709e-05 |
| Gene Ontology | [visit](http://www.godatabase.org/cgi-bin/amigo/go.cgi?view=details&search_constraint=terms&depth=0&query=GO:0032555) purine ribonucleotide binding | 318.937 | 398 | 1.07709e-05 |
| Gene Ontology | [visit](http://www.godatabase.org/cgi-bin/amigo/go.cgi?view=details&search_constraint=terms&depth=0&query=GO:0048870) cell motility | 85.2911 | 129 | 1.24432e-05 |
| Gene Ontology | [visit](http://www.godatabase.org/cgi-bin/amigo/go.cgi?view=details&search_constraint=terms&depth=0&query=GO:0051674) localization of cell | 85.2911 | 129 | 1.24432e-05 |
| Gene Ontology | [visit](http://www.godatabase.org/cgi-bin/amigo/go.cgi?view=details&search_constraint=terms&depth=0&query=GO:0043565) sequence-specific DNA binding | 111.654 | 161 | 1.25295e-05 |
| Gene Ontology | [visit](http://www.godatabase.org/cgi-bin/amigo/go.cgi?view=details&search_constraint=terms&depth=0&query=GO:0051641) cellular localization | 207.8 | 273 | 1.25295e-05 |
| Gene Ontology | [visit](http://www.godatabase.org/cgi-bin/amigo/go.cgi?view=details&search_constraint=terms&depth=0&query=GO:0071495) cellular response to endogenous stimulus | 32.738 | 61 | 1.25295e-05 |
| Gene Ontology | [visit](http://www.godatabase.org/cgi-bin/amigo/go.cgi?view=details&search_constraint=terms&depth=0&query=GO:0044463) cell projection part | 62.2022 | 100 | 1.25973e-05 |
| Gene Ontology | [visit](http://www.godatabase.org/cgi-bin/amigo/go.cgi?view=details&search_constraint=terms&depth=0&query=GO:0016323) basolateral plasma membrane | 38.5964 | 69 | 1.27296e-05 |
| Gene Ontology | [visit](http://www.godatabase.org/cgi-bin/amigo/go.cgi?view=details&search_constraint=terms&depth=0&query=GO:0034641) cellular nitrogen compound metabolic process | 789.847 | 900 | 1.37129e-05 |
| Gene Ontology | [visit](http://www.godatabase.org/cgi-bin/amigo/go.cgi?view=details&search_constraint=terms&depth=0&query=GO:0032989) cellular component morphogenesis | 81.5004 | 124 | 1.42482e-05 |
| Gene Ontology | [visit](http://www.godatabase.org/cgi-bin/amigo/go.cgi?view=details&search_constraint=terms&depth=0&query=GO:0008152) metabolic process | 1444.09 | 1568 | 1.50333e-05 |
| Gene Ontology | [visit](http://www.godatabase.org/cgi-bin/amigo/go.cgi?view=details&search_constraint=terms&depth=0&query=GO:0050793) regulation of developmental process | 139.05 | 193 | 1.5414e-05 |
| Gene Ontology | [visit](http://www.godatabase.org/cgi-bin/amigo/go.cgi?view=details&search_constraint=terms&depth=0&query=GO:0031175) neuron projection development | 55.4823 | 91 | 1.55048e-05 |
| Gene Ontology | [visit](http://www.godatabase.org/cgi-bin/amigo/go.cgi?view=details&search_constraint=terms&depth=0&query=GO:0048812) neuron projection morphogenesis | 42.5594 | 74 | 1.58981e-05 |
| Gene Ontology | [visit](http://www.godatabase.org/cgi-bin/amigo/go.cgi?view=details&search_constraint=terms&depth=0&query=GO:0044267) cellular protein metabolic process | 424.732 | 512 | 1.59741e-05 |
| Gene Ontology | [visit](http://www.godatabase.org/cgi-bin/amigo/go.cgi?view=details&search_constraint=terms&depth=0&query=GO:0045934) negative regulation of nucleobase, nucleoside, nucleotide and nucleic acid metabolic process | 98.9032 | 145 | 1.61898e-05 |
| Gene Ontology | [visit](http://www.godatabase.org/cgi-bin/amigo/go.cgi?view=details&search_constraint=terms&depth=0&query=GO:0015031) protein transport | 153.007 | 209 | 1.72292e-05 |
| Gene Ontology | [visit](http://www.godatabase.org/cgi-bin/amigo/go.cgi?view=details&search_constraint=terms&depth=0&query=GO:0000904) cell morphogenesis involved in differentiation | 52.5531 | 87 | 1.73179e-05 |
| Gene Ontology | [visit](http://www.godatabase.org/cgi-bin/amigo/go.cgi?view=details&search_constraint=terms&depth=0&query=GO:0001655) urogenital system development | 25.3289 | 50 | 1.8467e-05 |
| Gene Ontology | [visit](http://www.godatabase.org/cgi-bin/amigo/go.cgi?view=details&search_constraint=terms&depth=0&query=GO:0006996) organelle organization | 258.63 | 329 | 1.84683e-05 |
| Gene Ontology | [visit](http://www.godatabase.org/cgi-bin/amigo/go.cgi?view=details&search_constraint=terms&depth=0&query=GO:0006807) nitrogen compound  metabolic process | 809.49 | 918 | 2.22047e-05 |
| Gene Ontology | [visit](http://www.godatabase.org/cgi-bin/amigo/go.cgi?view=details&search_constraint=terms&depth=0&query=GO:0045595) regulation of cell differentiation | 97.0079 | 142 | 2.25193e-05 |
| Gene Ontology | [visit](http://www.godatabase.org/cgi-bin/amigo/go.cgi?view=details&search_constraint=terms&depth=0&query=GO:0019899) enzyme binding | 110.448 | 158 | 2.43519e-05 |
| Gene Ontology | [visit](http://www.godatabase.org/cgi-bin/amigo/go.cgi?view=details&search_constraint=terms&depth=0&query=GO:0040012) regulation of locomotion | 42.3871 | 73 | 2.66672e-05 |
| Gene Ontology | [visit](http://www.godatabase.org/cgi-bin/amigo/go.cgi?view=details&search_constraint=terms&depth=0&query=GO:0030425) dendrite | 32.0488 | 59 | 2.68446e-05 |
| Gene Ontology | [visit](http://www.godatabase.org/cgi-bin/amigo/go.cgi?view=details&search_constraint=terms&depth=0&query=GO:0051172) negative regulation of nitrogen compound metabolic process | 99.9371 | 145 | 2.89941e-05 |
| Gene Ontology | [visit](http://www.godatabase.org/cgi-bin/amigo/go.cgi?view=details&search_constraint=terms&depth=0&query=GO:0007423) sensory organ development | 43.2486 | 74 | 2.91161e-05 |
| Gene Ontology | [visit](http://www.godatabase.org/cgi-bin/amigo/go.cgi?view=details&search_constraint=terms&depth=0&query=GO:0008287) protein serinethreonine phosphatase complex | 6.71991 | 20 | 3.47632e-05 |
| Gene Ontology | [visit](http://www.godatabase.org/cgi-bin/amigo/go.cgi?view=details&search_constraint=terms&depth=0&query=GO:0046907) intracellular transport | 129.746 | 180 | 3.52732e-05 |
| Gene Ontology | [visit](http://www.godatabase.org/cgi-bin/amigo/go.cgi?view=details&search_constraint=terms&depth=0&query=GO:0005794) Golgi apparatus | 153.869 | 208 | 3.71685e-05 |
| Gene Ontology | [visit](http://www.godatabase.org/cgi-bin/amigo/go.cgi?view=details&search_constraint=terms&depth=0&query=GO:0003779) actin binding | 56.6884 | 91 | 3.86534e-05 |
| Gene Ontology | [visit](http://www.godatabase.org/cgi-bin/amigo/go.cgi?view=details&search_constraint=terms&depth=0&query=GO:0031324) negative regulation of cellular metabolic process | 139.395 | 191 | 3.94085e-05 |
| Gene Ontology | [visit](http://www.godatabase.org/cgi-bin/amigo/go.cgi?view=details&search_constraint=terms&depth=0&query=GO:0044431) Golgi apparatus part | 93.0448 | 136 | 3.94085e-05 |
| Gene Ontology | [visit](http://www.godatabase.org/cgi-bin/amigo/go.cgi?view=details&search_constraint=terms&depth=0&query=GO:0009892) negative regulation of metabolic process | 153.179 | 207 | 3.95465e-05 |
| Gene Ontology | [visit](http://www.godatabase.org/cgi-bin/amigo/go.cgi?view=details&search_constraint=terms&depth=0&query=GO:0051272) positive regulation of cellular component movement | 23.9504 | 47 | 4.39168e-05 |
| Gene Ontology | [visit](http://www.godatabase.org/cgi-bin/amigo/go.cgi?view=details&search_constraint=terms&depth=0&query=GO:0045597) positive regulation of cell differentiation | 44.6271 | 75 | 4.97871e-05 |
| Gene Ontology | [visit](http://www.godatabase.org/cgi-bin/amigo/go.cgi?view=details&search_constraint=terms&depth=0&query=GO:0017076) purine nucleotide binding | 333.238 | 408 | 5.04324e-05 |
| Gene Ontology | [visit](http://www.godatabase.org/cgi-bin/amigo/go.cgi?view=details&search_constraint=terms&depth=0&query=GO:0051270) regulation of cellular component movement | 42.3871 | 72 | 5.13084e-05 |
| Gene Ontology | [visit](http://www.godatabase.org/cgi-bin/amigo/go.cgi?view=details&search_constraint=terms&depth=0&query=GO:0033279) ribosomal subunit | 20.8489 | 4 | 5.64093e-05 |
| Gene Ontology | [visit](http://www.godatabase.org/cgi-bin/amigo/go.cgi?view=details&search_constraint=terms&depth=0&query=GO:0051094) positive regulation of developmental process | 61.1684 | 96 | 5.64093e-05 |
| Gene Ontology | [visit](http://www.godatabase.org/cgi-bin/amigo/go.cgi?view=details&search_constraint=terms&depth=0&query=GO:0030424) axon | 30.6703 | 56 | 6.1497e-05 |
| Gene Ontology | [visit](http://www.godatabase.org/cgi-bin/amigo/go.cgi?view=details&search_constraint=terms&depth=0&query=GO:0009792) embryonic development ending in birth or egg hatching | 65.3037 | 101 | 6.25344e-05 |
| Gene Ontology | [visit](http://www.godatabase.org/cgi-bin/amigo/go.cgi?view=details&search_constraint=terms&depth=0&query=GO:0016568) chromatin modification | 55.8269 | 89 | 6.44936e-05 |
| Gene Ontology | [visit](http://www.godatabase.org/cgi-bin/amigo/go.cgi?view=details&search_constraint=terms&depth=0&query=GO:0016044) cellular membrane organization | 76.6758 | 115 | 6.48864e-05 |
| Gene Ontology | [visit](http://www.godatabase.org/cgi-bin/amigo/go.cgi?view=details&search_constraint=terms&depth=0&query=GO:0048732) gland development | 35.1503 | 62 | 6.48864e-05 |
| Gene Ontology | [visit](http://www.godatabase.org/cgi-bin/amigo/go.cgi?view=details&search_constraint=terms&depth=0&query=GO:0043009) chordate embryonic development | 64.6145 | 100 | 6.65128e-05 |
| Gene Ontology | [visit](http://www.godatabase.org/cgi-bin/amigo/go.cgi?view=details&search_constraint=terms&depth=0&query=GO:0061024) membrane organization | 76.8482 | 115 | 7.18474e-05 |
| Gene Ontology | [visit](http://www.godatabase.org/cgi-bin/amigo/go.cgi?view=details&search_constraint=terms&depth=0&query=GO:0006468) protein amino acid phosphorylation | 139.05 | 189 | 7.1982e-05 |
| Gene Ontology | [visit](http://www.godatabase.org/cgi-bin/amigo/go.cgi?view=details&search_constraint=terms&depth=0&query=GO:0051649) establishment of localization in cell | 192.293 | 250 | 7.32252e-05 |
| Gene Ontology | [visit](http://www.godatabase.org/cgi-bin/amigo/go.cgi?view=details&search_constraint=terms&depth=0&query=GO:0007268) synaptic transmission | 62.3745 | 97 | 7.32656e-05 |
| Gene Ontology | [visit](http://www.godatabase.org/cgi-bin/amigo/go.cgi?view=details&search_constraint=terms&depth=0&query=GO:0006793) phosphorus metabolic process | 225.375 | 287 | 7.92316e-05 |
| Gene Ontology | [visit](http://www.godatabase.org/cgi-bin/amigo/go.cgi?view=details&search_constraint=terms&depth=0&query=GO:0006796) phosphate metabolic process | 225.375 | 287 | 7.92316e-05 |
| Gene Ontology | [visit](http://www.godatabase.org/cgi-bin/amigo/go.cgi?view=details&search_constraint=terms&depth=0&query=GO:0006897) endocytosis | 47.5563 | 78 | 7.92316e-05 |
| Gene Ontology | [visit](http://www.godatabase.org/cgi-bin/amigo/go.cgi?view=details&search_constraint=terms&depth=0&query=GO:0007264) small GTPase mediated signal transduction | 74.6082 | 112 | 7.92316e-05 |
| Gene Ontology | [visit](http://www.godatabase.org/cgi-bin/amigo/go.cgi?view=details&search_constraint=terms&depth=0&query=GO:0010324) membrane invagination | 47.5563 | 78 | 7.92316e-05 |
| Gene Ontology | [visit](http://www.godatabase.org/cgi-bin/amigo/go.cgi?view=details&search_constraint=terms&depth=0&query=GO:0048667) cell morphogenesis involved in neuron differentiation | 41.5256 | 70 | 8.72254e-05 |
| Gene Ontology | [visit](http://www.godatabase.org/cgi-bin/amigo/go.cgi?view=details&search_constraint=terms&depth=0&query=GO:0005768) endosome | 66.6821 | 102 | 8.82571e-05 |
| Gene Ontology | [visit](http://www.godatabase.org/cgi-bin/amigo/go.cgi?view=details&search_constraint=terms&depth=0&query=GO:0032559) adenyl ribonucleotide binding | 258.975 | 324 | 8.92747e-05 |
| Gene Ontology | [visit](http://www.godatabase.org/cgi-bin/amigo/go.cgi?view=details&search_constraint=terms&depth=0&query=GO:0003735) structural constituent of ribosome | 27.2242 | 8 | 9.1381e-05 |
| Gene Ontology | [visit](http://www.godatabase.org/cgi-bin/amigo/go.cgi?view=details&search_constraint=terms&depth=0&query=GO:0044445) cytosolic part | 25.5012 | 7 | 9.4538e-05 |
| Gene Ontology | [visit](http://www.godatabase.org/cgi-bin/amigo/go.cgi?view=details&search_constraint=terms&depth=0&query=GO:0006605) protein targeting | 47.0393 | 77 | 9.6041e-05 |
| Gene Ontology | [visit](http://www.godatabase.org/cgi-bin/amigo/go.cgi?view=details&search_constraint=terms&depth=0&query=GO:0040017) positive regulation of locomotion | 23.9504 | 46 | 9.6041e-05 |
| Gene Ontology | [visit](http://www.godatabase.org/cgi-bin/amigo/go.cgi?view=details&search_constraint=terms&depth=0&query=GO:0005769) early endosome | 23.2612 | 45 | 9.75756e-05 |
| Gene Ontology | [visit](http://www.godatabase.org/cgi-bin/amigo/go.cgi?view=details&search_constraint=terms&depth=0&query=GO:0019226) transmission of nerve impulse | 72.5405 | 109 | 9.77717e-05 |
| Gene Ontology | [visit](http://www.godatabase.org/cgi-bin/amigo/go.cgi?view=details&search_constraint=terms&depth=0&query=GO:0034097) response to cytokine stimulus | 18.4367 | 38 | 9.85724e-05 |
| Gene Ontology | [visit](http://www.godatabase.org/cgi-bin/amigo/go.cgi?view=details&search_constraint=terms&depth=0&query=GO:0009725) response to hormone stimulus | 79.2604 | 117 | 0.000109214 |
| Gene Ontology | [visit](http://www.godatabase.org/cgi-bin/amigo/go.cgi?view=details&search_constraint=terms&depth=0&query=GO:0009719) response to endogenous stimulus | 88.3926 | 128 | 0.000112039 |
| Gene Ontology | [visit](http://www.godatabase.org/cgi-bin/amigo/go.cgi?view=details&search_constraint=terms&depth=0&query=GO:0045892) negative regulation of transcription, DNA-dependent | 71.1621 | 107 | 0.000113589 |
| Gene Ontology | [visit](http://www.godatabase.org/cgi-bin/amigo/go.cgi?view=details&search_constraint=terms&depth=0&query=GO:0001944) vasculature development | 59.9622 | 93 | 0.000120101 |
| Gene Ontology | [visit](http://www.godatabase.org/cgi-bin/amigo/go.cgi?view=details&search_constraint=terms&depth=0&query=GO:0004842) ubiquitin-protein ligase activity | 32.9103 | 58 | 0.000123496 |
| Gene Ontology | [visit](http://www.godatabase.org/cgi-bin/amigo/go.cgi?view=details&search_constraint=terms&depth=0&query=GO:0005884) actin filament | 7.23682 | 20 | 0.000123496 |
| Gene Ontology | [visit](http://www.godatabase.org/cgi-bin/amigo/go.cgi?view=details&search_constraint=terms&depth=0&query=GO:0030334) regulation of cell migration | 38.2518 | 65 | 0.000133226 |
| Gene Ontology | [visit](http://www.godatabase.org/cgi-bin/amigo/go.cgi?view=details&search_constraint=terms&depth=0&query=GO:0051246) regulation of protein metabolic process | 112.343 | 156 | 0.000138115 |
| Gene Ontology | [visit](http://www.godatabase.org/cgi-bin/amigo/go.cgi?view=details&search_constraint=terms&depth=0&query=GO:0051253) negative regulation of RNA metabolic process | 72.3682 | 108 | 0.000143912 |
| Gene Ontology | [visit](http://www.godatabase.org/cgi-bin/amigo/go.cgi?view=details&search_constraint=terms&depth=0&query=GO:0016358) dendrite development | 9.6491 | 24 | 0.000144306 |
| Gene Ontology | [visit](http://www.godatabase.org/cgi-bin/amigo/go.cgi?view=details&search_constraint=terms&depth=0&query=GO:0006325) chromatin organization | 73.2297 | 109 | 0.000146951 |
| Gene Ontology | [visit](http://www.godatabase.org/cgi-bin/amigo/go.cgi?view=details&search_constraint=terms&depth=0&query=GO:0001501) skeletal system development | 65.9929 | 100 | 0.000154839 |
| Gene Ontology | [visit](http://www.godatabase.org/cgi-bin/amigo/go.cgi?view=details&search_constraint=terms&depth=0&query=GO:0030175) filopodium | 5.68607 | 17 | 0.000159917 |
| Gene Ontology | [visit](http://www.godatabase.org/cgi-bin/amigo/go.cgi?view=details&search_constraint=terms&depth=0&query=GO:0005912) adherens junction | 25.1566 | 47 | 0.000163822 |
| Gene Ontology | [visit](http://www.godatabase.org/cgi-bin/amigo/go.cgi?view=details&search_constraint=terms&depth=0&query=GO:0030335) positive regulation of cell migration | 22.3997 | 43 | 0.000183193 |
| Gene Ontology | [visit](http://www.godatabase.org/cgi-bin/amigo/go.cgi?view=details&search_constraint=terms&depth=0&query=GO:0001654) eye development | 26.7073 | 49 | 0.000184051 |
| Gene Ontology | [visit](http://www.godatabase.org/cgi-bin/amigo/go.cgi?view=details&search_constraint=terms&depth=0&query=GO:0030029) actin filament-based process | 51.8639 | 82 | 0.000185413 |
| Gene Ontology | [visit](http://www.godatabase.org/cgi-bin/amigo/go.cgi?view=details&search_constraint=terms&depth=0&query=GO:0048858) cell projection morphogenesis | 48.7624 | 78 | 0.000191964 |
| Gene Ontology | [visit](http://www.godatabase.org/cgi-bin/amigo/go.cgi?view=details&search_constraint=terms&depth=0&query=GO:0001701) in utero embryonic development | 38.7687 | 65 | 0.000205247 |
| Gene Ontology | [visit](http://www.godatabase.org/cgi-bin/amigo/go.cgi?view=details&search_constraint=terms&depth=0&query=GO:0007507) heart development | 41.8702 | 69 | 0.000209782 |
| Gene Ontology | [visit](http://www.godatabase.org/cgi-bin/amigo/go.cgi?view=details&search_constraint=terms&depth=0&query=GO:0034613) cellular protein localization | 86.3249 | 124 | 0.000219031 |
| Gene Ontology | [visit](http://www.godatabase.org/cgi-bin/amigo/go.cgi?view=details&search_constraint=terms&depth=0&query=GO:0031589) cell-substrate adhesion | 26.1904 | 48 | 0.000228133 |
| Gene Ontology | [visit](http://www.godatabase.org/cgi-bin/amigo/go.cgi?view=details&search_constraint=terms&depth=0&query=GO:0042742) defense response to bacterium | 20.8489 | 5 | 0.000231765 |
| Gene Ontology | [visit](http://www.godatabase.org/cgi-bin/amigo/go.cgi?view=details&search_constraint=terms&depth=0&query=GO:0001568) blood vessel development | 58.5838 | 90 | 0.000233909 |
| Gene Ontology | [visit](http://www.godatabase.org/cgi-bin/amigo/go.cgi?view=details&search_constraint=terms&depth=0&query=GO:0005524) ATP binding | 255.012 | 316 | 0.000239068 |
| Gene Ontology | [visit](http://www.godatabase.org/cgi-bin/amigo/go.cgi?view=details&search_constraint=terms&depth=0&query=GO:0005615) extracellular space | 129.574 | 88 | 0.00024877 |
| Gene Ontology | [visit](http://www.godatabase.org/cgi-bin/amigo/go.cgi?view=details&search_constraint=terms&depth=0&query=GO:0031528) microvillus membrane | 2.41227 | 10 | 0.000248923 |
| Gene Ontology | [visit](http://www.godatabase.org/cgi-bin/amigo/go.cgi?view=details&search_constraint=terms&depth=0&query=GO:0051130) positive regulation of cellular component organization | 39.8025 | 66 | 0.000252019 |
| Gene Ontology | [visit](http://www.godatabase.org/cgi-bin/amigo/go.cgi?view=details&search_constraint=terms&depth=0&query=GO:0070727) cellular macromolecule localization | 86.6696 | 124 | 0.00025941 |
| Gene Ontology | [visit](http://www.godatabase.org/cgi-bin/amigo/go.cgi?view=details&search_constraint=terms&depth=0&query=GO:0007409) axonogenesis | 37.5625 | 63 | 0.000264008 |
| Gene Ontology | [visit](http://www.godatabase.org/cgi-bin/amigo/go.cgi?view=details&search_constraint=terms&depth=0&query=GO:0010033) response to organic substance | 155.592 | 204 | 0.00028837 |
| Gene Ontology | [visit](http://www.godatabase.org/cgi-bin/amigo/go.cgi?view=details&search_constraint=terms&depth=0&query=GO:0040011) locomotion | 106.312 | 147 | 0.000291967 |
| Gene Ontology | [visit](http://www.godatabase.org/cgi-bin/amigo/go.cgi?view=details&search_constraint=terms&depth=0&query=GO:0046914) transition metal ion binding | 393.718 | 466 | 0.000301149 |
| Gene Ontology | [visit](http://www.godatabase.org/cgi-bin/amigo/go.cgi?view=details&search_constraint=terms&depth=0&query=GO:0048646) anatomical structure formation involved in morphogenesis | 81.3281 | 117 | 0.000340585 |
| Gene Ontology | [visit](http://www.godatabase.org/cgi-bin/amigo/go.cgi?view=details&search_constraint=terms&depth=0&query=GO:0023051) regulation of signaling process | 136.81 | 182 | 0.000342117 |
| Gene Ontology | [visit](http://www.godatabase.org/cgi-bin/amigo/go.cgi?view=details&search_constraint=terms&depth=0&query=GO:0044456) synapse part | 47.2116 | 75 | 0.000342117 |
| Gene Ontology | [visit](http://www.godatabase.org/cgi-bin/amigo/go.cgi?view=details&search_constraint=terms&depth=0&query=GO:0060541) respiratory system development | 19.4705 | 38 | 0.000359952 |
| Gene Ontology | [visit](http://www.godatabase.org/cgi-bin/amigo/go.cgi?view=details&search_constraint=terms&depth=0&query=GO:0006886) intracellular protein transport | 75.642 | 110 | 0.000361769 |
| Gene Ontology | [visit](http://www.godatabase.org/cgi-bin/amigo/go.cgi?view=details&search_constraint=terms&depth=0&query=GO:0030323) respiratory tube development | 18.0921 | 36 | 0.000364205 |
| Gene Ontology | [visit](http://www.godatabase.org/cgi-bin/amigo/go.cgi?view=details&search_constraint=terms&depth=0&query=GO:0030036) actin cytoskeleton organization | 48.9347 | 77 | 0.000376918 |
| Gene Ontology | [visit](http://www.godatabase.org/cgi-bin/amigo/go.cgi?view=details&search_constraint=terms&depth=0&query=GO:0009952) anteriorposterior pattern formation | 26.7073 | 48 | 0.000377339 |
| Gene Ontology | [visit](http://www.godatabase.org/cgi-bin/amigo/go.cgi?view=details&search_constraint=terms&depth=0&query=GO:0048589) developmental growth | 28.2581 | 50 | 0.000407356 |
| Gene Ontology | [visit](http://www.godatabase.org/cgi-bin/amigo/go.cgi?view=details&search_constraint=terms&depth=0&query=GO:0009888) tissue development | 142.496 | 188 | 0.000407515 |
| Gene Ontology | [visit](http://www.godatabase.org/cgi-bin/amigo/go.cgi?view=details&search_constraint=terms&depth=0&query=GO:0007010) cytoskeleton organization | 90.9772 | 128 | 0.000419132 |
| Gene Ontology | [visit](http://www.godatabase.org/cgi-bin/amigo/go.cgi?view=details&search_constraint=terms&depth=0&query=GO:0007389) pattern specification process | 49.107 | 77 | 0.000422489 |
| Gene Ontology | [visit](http://www.godatabase.org/cgi-bin/amigo/go.cgi?view=details&search_constraint=terms&depth=0&query=GO:0035295) tube development | 58.7561 | 89 | 0.000429887 |
| Gene Ontology | [visit](http://www.godatabase.org/cgi-bin/amigo/go.cgi?view=details&search_constraint=terms&depth=0&query=GO:0051276) chromosome organization | 94.4233 | 132 | 0.000430046 |
| Gene Ontology | [visit](http://www.godatabase.org/cgi-bin/amigo/go.cgi?view=details&search_constraint=terms&depth=0&query=GO:0007179) transforming growth factor beta receptor signaling pathway | 17.5751 | 35 | 0.000448551 |
| Gene Ontology | [visit](http://www.godatabase.org/cgi-bin/amigo/go.cgi?view=details&search_constraint=terms&depth=0&query=GO:0003682) chromatin binding | 30.6703 | 53 | 0.000473735 |
| Gene Ontology | [visit](http://www.godatabase.org/cgi-bin/amigo/go.cgi?view=details&search_constraint=terms&depth=0&query=GO:0030554) adenyl nucleotide binding | 272.932 | 333 | 0.000481508 |
| Gene Ontology | [visit](http://www.godatabase.org/cgi-bin/amigo/go.cgi?view=details&search_constraint=terms&depth=0&query=GO:0008134) transcription factor binding | 90.4603 | 127 | 0.000488448 |
| Gene Ontology | [visit](http://www.godatabase.org/cgi-bin/amigo/go.cgi?view=details&search_constraint=terms&depth=0&query=GO:0007267) cell-cell signaling | 124.06 | 166 | 0.000541512 |
| Gene Ontology | [visit](http://www.godatabase.org/cgi-bin/amigo/go.cgi?view=details&search_constraint=terms&depth=0&query=GO:0032990) cell part morphogenesis | 51.1747 | 79 | 0.000585652 |
| Gene Ontology | [visit](http://www.godatabase.org/cgi-bin/amigo/go.cgi?view=details&search_constraint=terms&depth=0&query=GO:0070410) co-SMAD binding | 1.37844 | 7 | 0.000590112 |
| Gene Ontology | [visit](http://www.godatabase.org/cgi-bin/amigo/go.cgi?view=details&search_constraint=terms&depth=0&query=GO:0007160) cell-matrix adhesion | 20.6766 | 39 | 0.000634994 |
| Gene Ontology | [visit](http://www.godatabase.org/cgi-bin/amigo/go.cgi?view=details&search_constraint=terms&depth=0&query=GO:0030695) GTPase regulator activity | 72.5405 | 105 | 0.000638333 |
| Gene Ontology | [visit](http://www.godatabase.org/cgi-bin/amigo/go.cgi?view=details&search_constraint=terms&depth=0&query=GO:0031253) cell projection membrane | 19.9874 | 38 | 0.000649207 |
| Gene Ontology | [visit](http://www.godatabase.org/cgi-bin/amigo/go.cgi?view=details&search_constraint=terms&depth=0&query=GO:0042221) response to chemical stimulus | 261.215 | 319 | 0.000651208 |
| Gene Ontology | [visit](http://www.godatabase.org/cgi-bin/amigo/go.cgi?view=details&search_constraint=terms&depth=0&query=GO:0070161) anchoring junction | 28.0858 | 49 | 0.00069238 |
| Gene Ontology | [visit](http://www.godatabase.org/cgi-bin/amigo/go.cgi?view=details&search_constraint=terms&depth=0&query=GO:0000166) nucleotide binding | 388.893 | 457 | 0.000697178 |
| Gene Ontology | [visit](http://www.godatabase.org/cgi-bin/amigo/go.cgi?view=details&search_constraint=terms&depth=0&query=GO:0007517) muscle organ development | 44.2825 | 70 | 0.000697178 |
| Gene Ontology | [visit](http://www.godatabase.org/cgi-bin/amigo/go.cgi?view=details&search_constraint=terms&depth=0&query=GO:0008219) cell death | 215.382 | 268 | 0.000742613 |
| Gene Ontology | [visit](http://www.godatabase.org/cgi-bin/amigo/go.cgi?view=details&search_constraint=terms&depth=0&query=GO:0001883) purine nucleoside binding | 279.135 | 338 | 0.000756607 |
| Gene Ontology | [visit](http://www.godatabase.org/cgi-bin/amigo/go.cgi?view=details&search_constraint=terms&depth=0&query=GO:0010648) negative regulation of cell communication | 58.0669 | 87 | 0.000760898 |
| Gene Ontology | [visit](http://www.godatabase.org/cgi-bin/amigo/go.cgi?view=details&search_constraint=terms&depth=0&query=GO:0031252) cell leading edge | 28.9473 | 50 | 0.000760898 |
| Gene Ontology | [visit](http://www.godatabase.org/cgi-bin/amigo/go.cgi?view=details&search_constraint=terms&depth=0&query=GO:0046332) SMAD binding | 8.09835 | 20 | 0.000768772 |
| Gene Ontology | [visit](http://www.godatabase.org/cgi-bin/amigo/go.cgi?view=details&search_constraint=terms&depth=0&query=GO:0071375) cellular response to peptide hormone stimulus | 17.4028 | 34 | 0.000852569 |
| Gene Ontology | [visit](http://www.godatabase.org/cgi-bin/amigo/go.cgi?view=details&search_constraint=terms&depth=0&query=GO:0016265) death | 215.899 | 268 | 0.000866491 |
| Gene Ontology | [visit](http://www.godatabase.org/cgi-bin/amigo/go.cgi?view=details&search_constraint=terms&depth=0&query=GO:0023057) negative regulation of signaling process | 21.7105 | 40 | 0.000879564 |
| Gene Ontology | [visit](http://www.godatabase.org/cgi-bin/amigo/go.cgi?view=details&search_constraint=terms&depth=0&query=GO:0016070) RNA metabolic process | 447.304 | 518 | 0.000922179 |
| Gene Ontology | [visit](http://www.godatabase.org/cgi-bin/amigo/go.cgi?view=details&search_constraint=terms&depth=0&query=GO:0060589) nucleoside-triphosphatase regulator activity | 74.0913 | 106 | 0.000927783 |
| Gene Ontology | [visit](http://www.godatabase.org/cgi-bin/amigo/go.cgi?view=details&search_constraint=terms&depth=0&query=GO:0001882) nucleoside binding | 280.858 | 339 | 0.000932165 |
| Gene Ontology | [visit](http://www.godatabase.org/cgi-bin/amigo/go.cgi?view=details&search_constraint=terms&depth=0&query=GO:0045121) membrane raft | 26.1904 | 46 | 0.000937807 |
| Gene Ontology | [visit](http://www.godatabase.org/cgi-bin/amigo/go.cgi?view=details&search_constraint=terms&depth=0&query=GO:0009966) regulation of signal transduction | 135.949 | 178 | 0.00094044 |
| Gene Ontology | [visit](http://www.godatabase.org/cgi-bin/amigo/go.cgi?view=details&search_constraint=terms&depth=0&query=GO:0048568) embryonic organ development | 36.8733 | 60 | 0.00094462 |
| Gene Ontology | [visit](http://www.godatabase.org/cgi-bin/amigo/go.cgi?view=details&search_constraint=terms&depth=0&query=GO:0070887) cellular response to chemical stimulus | 77.5374 | 110 | 0.000965205 |
| Gene Ontology | [visit](http://www.godatabase.org/cgi-bin/amigo/go.cgi?view=details&search_constraint=terms&depth=0&query=GO:0043234) protein complex | 461.778 | 533 | 0.00097834 |
| Gene Ontology | [visit](http://www.godatabase.org/cgi-bin/amigo/go.cgi?view=details&search_constraint=terms&depth=0&query=GO:0072001) renal system development | 18.9536 | 36 | 0.000981289 |
| Gene Ontology | [visit](http://www.godatabase.org/cgi-bin/amigo/go.cgi?view=details&search_constraint=terms&depth=0&query=GO:0019787) small conjugating protein ligase activity | 36.1841 | 59 | 0.00100126 |
| Gene Ontology | [visit](http://www.godatabase.org/cgi-bin/amigo/go.cgi?view=details&search_constraint=terms&depth=0&query=GO:0043434) response to peptide hormone stimulus | 34.6334 | 57 | 0.00100126 |
| Gene Ontology | [visit](http://www.godatabase.org/cgi-bin/amigo/go.cgi?view=details&search_constraint=terms&depth=0&query=GO:0030324) lung development | 17.5751 | 34 | 0.00101957 |
| Gene Ontology | [visit](http://www.godatabase.org/cgi-bin/amigo/go.cgi?view=details&search_constraint=terms&depth=0&query=GO:0005840) ribosome | 34.1164 | 15 | 0.00106942 |
| Gene Ontology | [visit](http://www.godatabase.org/cgi-bin/amigo/go.cgi?view=details&search_constraint=terms&depth=0&query=GO:0019902) phosphatase binding | 8.95987 | 21 | 0.0011928 |
| Gene Ontology | [visit](http://www.godatabase.org/cgi-bin/amigo/go.cgi?view=details&search_constraint=terms&depth=0&query=GO:0001822) kidney development | 18.4367 | 35 | 0.00122311 |
| Gene Ontology | [visit](http://www.godatabase.org/cgi-bin/amigo/go.cgi?view=details&search_constraint=terms&depth=0&query=GO:0016055) Wnt receptor signaling pathway | 32.5657 | 54 | 0.00122311 |
| Gene Ontology | [visit](http://www.godatabase.org/cgi-bin/amigo/go.cgi?view=details&search_constraint=terms&depth=0&query=GO:0007178) transmembrane receptor protein serinethreonine kinase signaling pathway | 28.775 | 49 | 0.00124721 |
| Gene Ontology | [visit](http://www.godatabase.org/cgi-bin/amigo/go.cgi?view=details&search_constraint=terms&depth=0&query=GO:0016564) transcription repressor activity | 58.9284 | 87 | 0.00124721 |
| Gene Ontology | [visit](http://www.godatabase.org/cgi-bin/amigo/go.cgi?view=details&search_constraint=terms&depth=0&query=GO:0009968) negative regulation of signal transduction | 21.3659 | 39 | 0.00128103 |
| Gene Ontology | [visit](http://www.godatabase.org/cgi-bin/amigo/go.cgi?view=details&search_constraint=terms&depth=0&query=GO:0030522) intracellular receptor mediated signaling pathway | 16.369 | 32 | 0.00128981 |
| Gene Ontology | [visit](http://www.godatabase.org/cgi-bin/amigo/go.cgi?view=details&search_constraint=terms&depth=0&query=GO:0017171) serine hydrolase activity | 30.8426 | 13 | 0.00136447 |
| Gene Ontology | [visit](http://www.godatabase.org/cgi-bin/amigo/go.cgi?view=details&search_constraint=terms&depth=0&query=GO:0001763) morphogenesis of a branching structure | 22.9166 | 41 | 0.00139202 |
| Gene Ontology | [visit](http://www.godatabase.org/cgi-bin/amigo/go.cgi?view=details&search_constraint=terms&depth=0&query=GO:0045177) apical part of cell | 35.8395 | 58 | 0.00139202 |
| Gene Ontology | [visit](http://www.godatabase.org/cgi-bin/amigo/go.cgi?view=details&search_constraint=terms&depth=0&query=GO:0048598) embryonic morphogenesis | 59.9622 | 88 | 0.00140094 |
| Gene Ontology | [visit](http://www.godatabase.org/cgi-bin/amigo/go.cgi?view=details&search_constraint=terms&depth=0&query=GO:0006511) ubiquitin-dependent protein catabolic process | 51.0024 | 77 | 0.00142186 |
| Gene Ontology | [visit](http://www.godatabase.org/cgi-bin/amigo/go.cgi?view=details&search_constraint=terms&depth=0&query=GO:0000139) Golgi membrane | 77.5374 | 109 | 0.00145101 |
| Gene Ontology | [visit](http://www.godatabase.org/cgi-bin/amigo/go.cgi?view=details&search_constraint=terms&depth=0&query=GO:0055123) digestive system development | 11.0275 | 24 | 0.00146494 |
| Gene Ontology | [visit](http://www.godatabase.org/cgi-bin/amigo/go.cgi?view=details&search_constraint=terms&depth=0&query=GO:0003676) nucleic acid binding | 565.334 | 640 | 0.00146672 |
| Gene Ontology | [visit](http://www.godatabase.org/cgi-bin/amigo/go.cgi?view=details&search_constraint=terms&depth=0&query=GO:0003704) specific RNA polymerase  II transcription factor activity | 7.23682 | 18 | 0.00148471 |
| Gene Ontology | [visit](http://www.godatabase.org/cgi-bin/amigo/go.cgi?view=details&search_constraint=terms&depth=0&query=GO:0000122) negative regulation of transcription from RNA polymerase  II promoter | 50.3131 | 76 | 0.00151712 |
| Gene Ontology | [visit](http://www.godatabase.org/cgi-bin/amigo/go.cgi?view=details&search_constraint=terms&depth=0&query=GO:0048729) tissue morphogenesis | 50.3131 | 76 | 0.00151712 |
| Gene Ontology | [visit](http://www.godatabase.org/cgi-bin/amigo/go.cgi?view=details&search_constraint=terms&depth=0&query=GO:0021700) developmental maturation | 17.2305 | 33 | 0.00152338 |
| Gene Ontology | [visit](http://www.godatabase.org/cgi-bin/amigo/go.cgi?view=details&search_constraint=terms&depth=0&query=GO:0006417) regulation of translation | 26.0181 | 45 | 0.00153442 |
| Gene Ontology | [visit](http://www.godatabase.org/cgi-bin/amigo/go.cgi?view=details&search_constraint=terms&depth=0&query=GO:0051049) regulation of transport | 90.4603 | 124 | 0.00156263 |
| Gene Ontology | [visit](http://www.godatabase.org/cgi-bin/amigo/go.cgi?view=details&search_constraint=terms&depth=0&query=GO:0008285) negative regulation of cell proliferation | 65.9929 | 95 | 0.00156558 |
| Gene Ontology | [visit](http://www.godatabase.org/cgi-bin/amigo/go.cgi?view=details&search_constraint=terms&depth=0&query=GO:0032869) cellular response to insulin stimulus | 15.8521 | 31 | 0.00156575 |
| Gene Ontology | [visit](http://www.godatabase.org/cgi-bin/amigo/go.cgi?view=details&search_constraint=terms&depth=0&query=GO:0014069) postsynaptic density | 13.7844 | 28 | 0.00158389 |
| Gene Ontology | [visit](http://www.godatabase.org/cgi-bin/amigo/go.cgi?view=details&search_constraint=terms&depth=0&query=GO:0019941) modification-dependent protein catabolic process | 52.0362 | 78 | 0.00158413 |
| Gene Ontology | [visit](http://www.godatabase.org/cgi-bin/amigo/go.cgi?view=details&search_constraint=terms&depth=0&query=GO:0043632) modification-dependent macromolecule catabolic process | 52.0362 | 78 | 0.00158413 |
| Gene Ontology | [visit](http://www.godatabase.org/cgi-bin/amigo/go.cgi?view=details&search_constraint=terms&depth=0&query=GO:0002009) morphogenesis of an epithelium | 38.4241 | 61 | 0.00159759 |
| Gene Ontology | [visit](http://www.godatabase.org/cgi-bin/amigo/go.cgi?view=details&search_constraint=terms&depth=0&query=GO:0061061) muscle structure development | 52.8977 | 79 | 0.00162523 |
| Gene Ontology | [visit](http://www.godatabase.org/cgi-bin/amigo/go.cgi?view=details&search_constraint=terms&depth=0&query=GO:0008013) beta-catenin binding | 7.92604 | 19 | 0.00167367 |
| Gene Ontology | [visit](http://www.godatabase.org/cgi-bin/amigo/go.cgi?view=details&search_constraint=terms&depth=0&query=GO:0030900) forebrain development | 29.9811 | 50 | 0.00174583 |
| Gene Ontology | [visit](http://www.godatabase.org/cgi-bin/amigo/go.cgi?view=details&search_constraint=terms&depth=0&query=GO:0008236) serine-type peptidase activity | 30.3257 | 13 | 0.00180756 |
| Gene Ontology | [visit](http://www.godatabase.org/cgi-bin/amigo/go.cgi?view=details&search_constraint=terms&depth=0&query=GO:0070936) protein K48-linked ubiquitination | 4.47994 | 13 | 0.00202495 |
| Gene Ontology | [visit](http://www.godatabase.org/cgi-bin/amigo/go.cgi?view=details&search_constraint=terms&depth=0&query=GO:0000910) cytokinesis | 9.30448 | 21 | 0.00204481 |
| Gene Ontology | [visit](http://www.godatabase.org/cgi-bin/amigo/go.cgi?view=details&search_constraint=terms&depth=0&query=GO:0006414) translational elongation | 17.9197 | 5 | 0.00204481 |
| Gene Ontology | [visit](http://www.godatabase.org/cgi-bin/amigo/go.cgi?view=details&search_constraint=terms&depth=0&query=GO:0030165) PDZ domain binding | 9.30448 | 21 | 0.00204481 |
| Gene Ontology | [visit](http://www.godatabase.org/cgi-bin/amigo/go.cgi?view=details&search_constraint=terms&depth=0&query=GO:0031226) intrinsic to plasma membrane | 208.145 | 256 | 0.00205857 |
| Gene Ontology | [visit](http://www.godatabase.org/cgi-bin/amigo/go.cgi?view=details&search_constraint=terms&depth=0&query=GO:0019538) protein metabolic process | 505.716 | 575 | 0.00218929 |
| Gene Ontology | [visit](http://www.godatabase.org/cgi-bin/amigo/go.cgi?view=details&search_constraint=terms&depth=0&query=GO:0012501) programmed cell death | 196.6 | 243 | 0.0022012 |
| Gene Ontology | [visit](http://www.godatabase.org/cgi-bin/amigo/go.cgi?view=details&search_constraint=terms&depth=0&query=GO:0012505) endomembrane system | 250.36 | 302 | 0.0022012 |
| Gene Ontology | [visit](http://www.godatabase.org/cgi-bin/amigo/go.cgi?view=details&search_constraint=terms&depth=0&query=GO:0002520) immune system development | 62.5468 | 90 | 0.00226981 |
| Gene Ontology | [visit](http://www.godatabase.org/cgi-bin/amigo/go.cgi?view=details&search_constraint=terms&depth=0&query=GO:0019903) protein phosphatase binding | 8.09835 | 19 | 0.00227608 |
| Gene Ontology | [visit](http://www.godatabase.org/cgi-bin/amigo/go.cgi?view=details&search_constraint=terms&depth=0&query=GO:0031981) nuclear lumen | 261.559 | 314 | 0.00229816 |
| Gene Ontology | [visit](http://www.godatabase.org/cgi-bin/amigo/go.cgi?view=details&search_constraint=terms&depth=0&query=GO:0007185) transmembrane receptor protein tyrosine phosphatase signaling pathway | 1.20614 | 6 | 0.00239055 |
| Gene Ontology | [visit](http://www.godatabase.org/cgi-bin/amigo/go.cgi?view=details&search_constraint=terms&depth=0&query=GO:0030001) metal ion transport | 85.4634 | 117 | 0.00239682 |
| Gene Ontology | [visit](http://www.godatabase.org/cgi-bin/amigo/go.cgi?view=details&search_constraint=terms&depth=0&query=GO:0016881) acid-amino acid ligase activity | 42.2148 | 65 | 0.00240715 |
| Gene Ontology | [visit](http://www.godatabase.org/cgi-bin/amigo/go.cgi?view=details&search_constraint=terms&depth=0&query=GO:0032268) regulation of cellular protein metabolic process | 99.2478 | 133 | 0.00240715 |
| Gene Ontology | [visit](http://www.godatabase.org/cgi-bin/amigo/go.cgi?view=details&search_constraint=terms&depth=0&query=GO:0048592) eye morphogenesis | 14.129 | 28 | 0.00240715 |
| Gene Ontology | [visit](http://www.godatabase.org/cgi-bin/amigo/go.cgi?view=details&search_constraint=terms&depth=0&query=GO:0003712) transcription cofactor activity | 62.7191 | 90 | 0.00245667 |
| Gene Ontology | [visit](http://www.godatabase.org/cgi-bin/amigo/go.cgi?view=details&search_constraint=terms&depth=0&query=GO:0005743) mitochondrial inner membrane | 52.5531 | 30 | 0.00254744 |
| Gene Ontology | [visit](http://www.godatabase.org/cgi-bin/amigo/go.cgi?view=details&search_constraint=terms&depth=0&query=GO:0042325) regulation of phosphorylation | 91.6664 | 124 | 0.00259649 |
| Gene Ontology | [visit](http://www.godatabase.org/cgi-bin/amigo/go.cgi?view=details&search_constraint=terms&depth=0&query=GO:0048565) digestive tract development | 9.47679 | 21 | 0.00264924 |
| Gene Ontology | [visit](http://www.godatabase.org/cgi-bin/amigo/go.cgi?view=details&search_constraint=terms&depth=0&query=GO:0004872) receptor activity | 290.851 | 239 | 0.00286605 |
| Gene Ontology | [visit](http://www.godatabase.org/cgi-bin/amigo/go.cgi?view=details&search_constraint=terms&depth=0&query=GO:0030529) ribonucleoprotein complex | 86.8419 | 58 | 0.00292259 |
| Gene Ontology | [visit](http://www.godatabase.org/cgi-bin/amigo/go.cgi?view=details&search_constraint=terms&depth=0&query=GO:0004252) serine-type endopeptidase activity | 26.535 | 11 | 0.00313208 |
| Gene Ontology | [visit](http://www.godatabase.org/cgi-bin/amigo/go.cgi?view=details&search_constraint=terms&depth=0&query=GO:0050790) regulation of catalytic activity | 158.176 | 199 | 0.00315781 |
| Gene Ontology | [visit](http://www.godatabase.org/cgi-bin/amigo/go.cgi?view=details&search_constraint=terms&depth=0&query=GO:0005198) structural molecule activity | 104.417 | 73 | 0.00319307 |
| Gene Ontology | [visit](http://www.godatabase.org/cgi-bin/amigo/go.cgi?view=details&search_constraint=terms&depth=0&query=GO:0016324) apical plasma membrane | 28.4304 | 47 | 0.00319626 |
| Gene Ontology | [visit](http://www.godatabase.org/cgi-bin/amigo/go.cgi?view=details&search_constraint=terms&depth=0&query=GO:0006915) apoptosis | 195.222 | 240 | 0.00322397 |
| Gene Ontology | [visit](http://www.godatabase.org/cgi-bin/amigo/go.cgi?view=details&search_constraint=terms&depth=0&query=GO:0005887) integral to plasma membrane | 204.354 | 250 | 0.00325171 |
| Gene Ontology | [visit](http://www.godatabase.org/cgi-bin/amigo/go.cgi?view=details&search_constraint=terms&depth=0&query=GO:0005901) caveola | 8.95987 | 20 | 0.00325171 |
| Gene Ontology | [visit](http://www.godatabase.org/cgi-bin/amigo/go.cgi?view=details&search_constraint=terms&depth=0&query=GO:0017124) SH3 domain binding | 17.2305 | 32 | 0.00325171 |
| Gene Ontology | [visit](http://www.godatabase.org/cgi-bin/amigo/go.cgi?view=details&search_constraint=terms&depth=0&query=GO:0016020) membrane | 1255.42 | 1343 | 0.00328825 |
| Gene Ontology | [visit](http://www.godatabase.org/cgi-bin/amigo/go.cgi?view=details&search_constraint=terms&depth=0&query=GO:0065009) regulation of molecular function | 186.262 | 230 | 0.00328825 |
| Gene Ontology | [visit](http://www.godatabase.org/cgi-bin/amigo/go.cgi?view=details&search_constraint=terms&depth=0&query=GO:0044428) nuclear part | 333.928 | 390 | 0.00359097 |
| Gene Ontology | [visit](http://www.godatabase.org/cgi-bin/amigo/go.cgi?view=details&search_constraint=terms&depth=0&query=GO:0016310) phosphorylation | 196.6 | 241 | 0.00367491 |
| Gene Ontology | [visit](http://www.godatabase.org/cgi-bin/amigo/go.cgi?view=details&search_constraint=terms&depth=0&query=GO:0043549) regulation of kinase activity | 67.716 | 95 | 0.00369364 |
| Gene Ontology | [visit](http://www.godatabase.org/cgi-bin/amigo/go.cgi?view=details&search_constraint=terms&depth=0&query=GO:0035264) multicellular organism growth | 12.406 | 25 | 0.00373432 |
| Gene Ontology | [visit](http://www.godatabase.org/cgi-bin/amigo/go.cgi?view=details&search_constraint=terms&depth=0&query=GO:0003002) regionalization | 38.941 | 60 | 0.00389242 |
| Gene Ontology | [visit](http://www.godatabase.org/cgi-bin/amigo/go.cgi?view=details&search_constraint=terms&depth=0&query=GO:0043583) ear development | 17.4028 | 32 | 0.00389732 |
| Gene Ontology | [visit](http://www.godatabase.org/cgi-bin/amigo/go.cgi?view=details&search_constraint=terms&depth=0&query=GO:0005654) nucleoplasm | 161.795 | 202 | 0.00419727 |
| Gene Ontology | [visit](http://www.godatabase.org/cgi-bin/amigo/go.cgi?view=details&search_constraint=terms&depth=0&query=GO:0016491) oxidoreductase activity | 118.718 | 86 | 0.00421601 |
| Gene Ontology | [visit](http://www.godatabase.org/cgi-bin/amigo/go.cgi?view=details&search_constraint=terms&depth=0&query=GO:0051716) cellular response to stimulus | 189.88 | 233 | 0.00428557 |
| Gene Ontology | [visit](http://www.godatabase.org/cgi-bin/amigo/go.cgi?view=details&search_constraint=terms&depth=0&query=GO:0051603) proteolysis involved in cellular protein catabolic process | 59.6176 | 85 | 0.00429453 |
| Gene Ontology | [visit](http://www.godatabase.org/cgi-bin/amigo/go.cgi?view=details&search_constraint=terms&depth=0&query=GO:0043010) camera-type eye development | 21.1935 | 37 | 0.0043122 |
| Gene Ontology | [visit](http://www.godatabase.org/cgi-bin/amigo/go.cgi?view=details&search_constraint=terms&depth=0&query=GO:0044451) nucleoplasm part | 103.383 | 136 | 0.00440417 |
| Gene Ontology | [visit](http://www.godatabase.org/cgi-bin/amigo/go.cgi?view=details&search_constraint=terms&depth=0&query=GO:0060537) muscle tissue development | 29.6365 | 48 | 0.0044196 |
| Gene Ontology | [visit](http://www.godatabase.org/cgi-bin/amigo/go.cgi?view=details&search_constraint=terms&depth=0&query=GO:0030850) prostate gland development | 7.23682 | 17 | 0.0044691 |
| Gene Ontology | [visit](http://www.godatabase.org/cgi-bin/amigo/go.cgi?view=details&search_constraint=terms&depth=0&query=GO:0042127) regulation of cell proliferation | 146.804 | 185 | 0.00449279 |
| Gene Ontology | [visit](http://www.godatabase.org/cgi-bin/amigo/go.cgi?view=details&search_constraint=terms&depth=0&query=GO:0043274) phospholipase binding | 1.72305 | 7 | 0.00459616 |
| Gene Ontology | [visit](http://www.godatabase.org/cgi-bin/amigo/go.cgi?view=details&search_constraint=terms&depth=0&query=GO:0035107) appendage morphogenesis | 17.5751 | 32 | 0.00460406 |
| Gene Ontology | [visit](http://www.godatabase.org/cgi-bin/amigo/go.cgi?view=details&search_constraint=terms&depth=0&query=GO:0035108) limb morphogenesis | 17.5751 | 32 | 0.00460406 |
| Gene Ontology | [visit](http://www.godatabase.org/cgi-bin/amigo/go.cgi?view=details&search_constraint=terms&depth=0&query=GO:0004721) phosphoprotein phosphatase activity | 28.9473 | 47 | 0.00469614 |
| Gene Ontology | [visit](http://www.godatabase.org/cgi-bin/amigo/go.cgi?view=details&search_constraint=terms&depth=0&query=GO:0046323) glucose import | 6.03068 | 15 | 0.00469614 |
| Gene Ontology | [visit](http://www.godatabase.org/cgi-bin/amigo/go.cgi?view=details&search_constraint=terms&depth=0&query=GO:0019220) regulation of phosphate metabolic process | 95.8017 | 127 | 0.00481792 |
| Gene Ontology | [visit](http://www.godatabase.org/cgi-bin/amigo/go.cgi?view=details&search_constraint=terms&depth=0&query=GO:0051174) regulation of phosphorus metabolic process | 95.8017 | 127 | 0.00481792 |
| Gene Ontology | [visit](http://www.godatabase.org/cgi-bin/amigo/go.cgi?view=details&search_constraint=terms&depth=0&query=GO:0010608) posttranscriptional regulation of gene expression | 42.5594 | 64 | 0.00490362 |
| Gene Ontology | [visit](http://www.godatabase.org/cgi-bin/amigo/go.cgi?view=details&search_constraint=terms&depth=0&query=GO:0045211) postsynaptic membrane | 26.7073 | 44 | 0.00499879 |
| Gene Ontology | [visit](http://www.godatabase.org/cgi-bin/amigo/go.cgi?view=details&search_constraint=terms&depth=0&query=GO:0000932) cytoplasmic mRNA processing body | 4.30763 | 12 | 0.00512926 |
| Gene Ontology | [visit](http://www.godatabase.org/cgi-bin/amigo/go.cgi?view=details&search_constraint=terms&depth=0&query=GO:0001667) ameboidal cell migration | 8.61526 | 19 | 0.00515614 |
| Gene Ontology | [visit](http://www.godatabase.org/cgi-bin/amigo/go.cgi?view=details&search_constraint=terms&depth=0&query=GO:0048736) appendage development | 18.4367 | 33 | 0.00517485 |
| Gene Ontology | [visit](http://www.godatabase.org/cgi-bin/amigo/go.cgi?view=details&search_constraint=terms&depth=0&query=GO:0060173) limb development | 18.4367 | 33 | 0.00517485 |
| Gene Ontology | [visit](http://www.godatabase.org/cgi-bin/amigo/go.cgi?view=details&search_constraint=terms&depth=0&query=GO:0003713) transcription coactivator activity | 37.0456 | 57 | 0.00532102 |
| Gene Ontology | [visit](http://www.godatabase.org/cgi-bin/amigo/go.cgi?view=details&search_constraint=terms&depth=0&query=GO:0048839) inner ear development | 14.129 | 27 | 0.00539553 |
| Gene Ontology | [visit](http://www.godatabase.org/cgi-bin/amigo/go.cgi?view=details&search_constraint=terms&depth=0&query=GO:0044257) cellular protein catabolic process | 60.1345 | 85 | 0.00546543 |
| Gene Ontology | [visit](http://www.godatabase.org/cgi-bin/amigo/go.cgi?view=details&search_constraint=terms&depth=0&query=GO:0050808) synapse organization | 12.7506 | 25 | 0.00560118 |
| Gene Ontology | [visit](http://www.godatabase.org/cgi-bin/amigo/go.cgi?view=details&search_constraint=terms&depth=0&query=GO:0061008) hepaticobiliary system development | 11.3721 | 23 | 0.00566327 |
| Gene Ontology | [visit](http://www.godatabase.org/cgi-bin/amigo/go.cgi?view=details&search_constraint=terms&depth=0&query=GO:0005154) epidermal growth factor receptor binding | 2.23997 | 8 | 0.00573295 |
| Gene Ontology | [visit](http://www.godatabase.org/cgi-bin/amigo/go.cgi?view=details&search_constraint=terms&depth=0&query=GO:0006607) NLS-bearing substrate import into nucleus | 2.23997 | 8 | 0.00573295 |
| Gene Ontology | [visit](http://www.godatabase.org/cgi-bin/amigo/go.cgi?view=details&search_constraint=terms&depth=0&query=GO:0010559) regulation of glycoprotein biosynthetic process | 2.23997 | 8 | 0.00573295 |
| Gene Ontology | [visit](http://www.godatabase.org/cgi-bin/amigo/go.cgi?view=details&search_constraint=terms&depth=0&query=GO:0015085) calcium ion transmembrane transporter activity | 2.23997 | 8 | 0.00573295 |
| Gene Ontology | [visit](http://www.godatabase.org/cgi-bin/amigo/go.cgi?view=details&search_constraint=terms&depth=0&query=GO:0010941) regulation of cell death | 155.764 | 194 | 0.00576574 |
| Gene Ontology | [visit](http://www.godatabase.org/cgi-bin/amigo/go.cgi?view=details&search_constraint=terms&depth=0&query=GO:0048534) hemopoietic or lymphoid organ development | 58.5838 | 83 | 0.00576574 |
| Gene Ontology | [visit](http://www.godatabase.org/cgi-bin/amigo/go.cgi?view=details&search_constraint=terms&depth=0&query=GO:0005096) GTPase activator activity | 39.6302 | 60 | 0.00584 |
| Gene Ontology | [visit](http://www.godatabase.org/cgi-bin/amigo/go.cgi?view=details&search_constraint=terms&depth=0&query=GO:0008047) enzyme activator activity | 60.3068 | 85 | 0.00587208 |
| Gene Ontology | [visit](http://www.godatabase.org/cgi-bin/amigo/go.cgi?view=details&search_constraint=terms&depth=0&query=GO:0060284) regulation of cell development | 46.1778 | 68 | 0.00590488 |
| Gene Ontology | [visit](http://www.godatabase.org/cgi-bin/amigo/go.cgi?view=details&search_constraint=terms&depth=0&query=GO:0010564) regulation of cell cycle process | 24.6397 | 41 | 0.00593266 |
| Gene Ontology | [visit](http://www.godatabase.org/cgi-bin/amigo/go.cgi?view=details&search_constraint=terms&depth=0&query=GO:0031103) axon regeneration | 3.2738 | 10 | 0.00597702 |
| Gene Ontology | [visit](http://www.godatabase.org/cgi-bin/amigo/go.cgi?view=details&search_constraint=terms&depth=0&query=GO:0006119) oxidative phosphorylation | 17.9197 | 6 | 0.00613779 |
| Gene Ontology | [visit](http://www.godatabase.org/cgi-bin/amigo/go.cgi?view=details&search_constraint=terms&depth=0&query=GO:0005902) microvillus | 8.09835 | 18 | 0.00619407 |
| Gene Ontology | [visit](http://www.godatabase.org/cgi-bin/amigo/go.cgi?view=details&search_constraint=terms&depth=0&query=GO:0007416) synapse assembly | 8.09835 | 18 | 0.00619407 |
| Gene Ontology | [visit](http://www.godatabase.org/cgi-bin/amigo/go.cgi?view=details&search_constraint=terms&depth=0&query=GO:0060740) prostate gland epithelium morphogenesis | 4.99685 | 13 | 0.00639694 |
| Gene Ontology | [visit](http://www.godatabase.org/cgi-bin/amigo/go.cgi?view=details&search_constraint=terms&depth=0&query=GO:0035467) negative regulation of signaling pathway | 46.3501 | 68 | 0.0064835 |
| Gene Ontology | [visit](http://www.godatabase.org/cgi-bin/amigo/go.cgi?view=details&search_constraint=terms&depth=0&query=GO:0032331) negative regulation of chondrocyte differentiation | 1.37844 | 6 | 0.00677804 |
| Gene Ontology | [visit](http://www.godatabase.org/cgi-bin/amigo/go.cgi?view=details&search_constraint=terms&depth=0&query=GO:0042578) phosphoric ester hydrolase activity | 58.9284 | 83 | 0.00679278 |
| Gene Ontology | [visit](http://www.godatabase.org/cgi-bin/amigo/go.cgi?view=details&search_constraint=terms&depth=0&query=GO:0044444) cytoplasmic part | 893.231 | 969 | 0.00683754 |
| Gene Ontology | [visit](http://www.godatabase.org/cgi-bin/amigo/go.cgi?view=details&search_constraint=terms&depth=0&query=GO:0016874) ligase activity | 75.1251 | 102 | 0.00689695 |
| Gene Ontology | [visit](http://www.godatabase.org/cgi-bin/amigo/go.cgi?view=details&search_constraint=terms&depth=0&query=GO:0003924) GTPase activity | 36.701 | 56 | 0.0069892 |
| Gene Ontology | [visit](http://www.godatabase.org/cgi-bin/amigo/go.cgi?view=details&search_constraint=terms&depth=0&query=GO:0016311) dephosphorylation | 30.3257 | 48 | 0.00701818 |
| Gene Ontology | [visit](http://www.godatabase.org/cgi-bin/amigo/go.cgi?view=details&search_constraint=terms&depth=0&query=GO:0022804) active transmembrane transporter activity | 59.1007 | 83 | 0.00738617 |
| Gene Ontology | [visit](http://www.godatabase.org/cgi-bin/amigo/go.cgi?view=details&search_constraint=terms&depth=0&query=GO:0000118) histone deacetylase complex | 7.58143 | 17 | 0.00763536 |
| Gene Ontology | [visit](http://www.godatabase.org/cgi-bin/amigo/go.cgi?view=details&search_constraint=terms&depth=0&query=GO:0030097) hemopoiesis | 54.9654 | 78 | 0.00763536 |
| Gene Ontology | [visit](http://www.godatabase.org/cgi-bin/amigo/go.cgi?view=details&search_constraint=terms&depth=0&query=GO:0016585) chromatin remodeling complex | 14.4736 | 27 | 0.00769103 |
| Gene Ontology | [visit](http://www.godatabase.org/cgi-bin/amigo/go.cgi?view=details&search_constraint=terms&depth=0&query=GO:0040008) regulation of growth | 64.2699 | 89 | 0.00769103 |
| Gene Ontology | [visit](http://www.godatabase.org/cgi-bin/amigo/go.cgi?view=details&search_constraint=terms&depth=0&query=GO:0048514) blood vessel morphogenesis | 50.8301 | 73 | 0.00776996 |
| Gene Ontology | [visit](http://www.godatabase.org/cgi-bin/amigo/go.cgi?view=details&search_constraint=terms&depth=0&query=GO:0051338) regulation of transferase activity | 70.3006 | 96 | 0.00790328 |
| Gene Ontology | [visit](http://www.godatabase.org/cgi-bin/amigo/go.cgi?view=details&search_constraint=terms&depth=0&query=GO:0010942) positive regulation of cell death | 81.5004 | 109 | 0.00790462 |
| Gene Ontology | [visit](http://www.godatabase.org/cgi-bin/amigo/go.cgi?view=details&search_constraint=terms&depth=0&query=GO:0060070) canonical Wnt receptor signaling pathway | 13.7844 | 26 | 0.00790462 |
| Gene Ontology | [visit](http://www.godatabase.org/cgi-bin/amigo/go.cgi?view=details&search_constraint=terms&depth=0&query=GO:0005938) cell cortex | 22.7443 | 38 | 0.00810909 |
| Gene Ontology | [visit](http://www.godatabase.org/cgi-bin/amigo/go.cgi?view=details&search_constraint=terms&depth=0&query=GO:0016773) phosphotransferase activity, alcohol group as acceptor | 121.992 | 155 | 0.00810909 |
| Gene Ontology | [visit](http://www.godatabase.org/cgi-bin/amigo/go.cgi?view=details&search_constraint=terms&depth=0&query=GO:0022604) regulation of cell morphogenesis | 27.3965 | 44 | 0.00810909 |
| Gene Ontology | [visit](http://www.godatabase.org/cgi-bin/amigo/go.cgi?view=details&search_constraint=terms&depth=0&query=GO:0030100) regulation of endocytosis | 13.0952 | 25 | 0.00810909 |
| Gene Ontology | [visit](http://www.godatabase.org/cgi-bin/amigo/go.cgi?view=details&search_constraint=terms&depth=0&query=GO:0065008) regulation of biological quality | 296.882 | 346 | 0.00810909 |
| Gene Ontology | [visit](http://www.godatabase.org/cgi-bin/amigo/go.cgi?view=details&search_constraint=terms&depth=0&query=GO:0004879) ligand-dependent nuclear receptor activity | 8.95987 | 19 | 0.00821424 |
| Gene Ontology | [visit](http://www.godatabase.org/cgi-bin/amigo/go.cgi?view=details&search_constraint=terms&depth=0&query=GO:0016197) endosome transport | 12.406 | 24 | 0.00821752 |
| Gene Ontology | [visit](http://www.godatabase.org/cgi-bin/amigo/go.cgi?view=details&search_constraint=terms&depth=0&query=GO:0045859) regulation of protein kinase activity | 65.3037 | 90 | 0.00822974 |
| Gene Ontology | [visit](http://www.godatabase.org/cgi-bin/amigo/go.cgi?view=details&search_constraint=terms&depth=0&query=GO:0048538) thymus development | 3.96302 | 11 | 0.00831507 |
| Gene Ontology | [visit](http://www.godatabase.org/cgi-bin/amigo/go.cgi?view=details&search_constraint=terms&depth=0&query=GO:0060322) head development | 3.96302 | 11 | 0.00831507 |
| Gene Ontology | [visit](http://www.godatabase.org/cgi-bin/amigo/go.cgi?view=details&search_constraint=terms&depth=0&query=GO:0001657) ureteric bud development | 10.3383 | 21 | 0.00835768 |
| Gene Ontology | [visit](http://www.godatabase.org/cgi-bin/amigo/go.cgi?view=details&search_constraint=terms&depth=0&query=GO:0007265) Ras protein signal transduction | 39.4579 | 59 | 0.00835768 |
| Gene Ontology | [visit](http://www.godatabase.org/cgi-bin/amigo/go.cgi?view=details&search_constraint=terms&depth=0&query=GO:0009791) post-embryonic development | 11.0275 | 22 | 0.00835768 |
| Gene Ontology | [visit](http://www.godatabase.org/cgi-bin/amigo/go.cgi?view=details&search_constraint=terms&depth=0&query=GO:0046873) metal ion transmembrane transporter activity | 22.0551 | 37 | 0.00852651 |
| Gene Ontology | [visit](http://www.godatabase.org/cgi-bin/amigo/go.cgi?view=details&search_constraint=terms&depth=0&query=GO:0032868) response to insulin stimulus | 23.6058 | 39 | 0.00862091 |
| Gene Ontology | [visit](http://www.godatabase.org/cgi-bin/amigo/go.cgi?view=details&search_constraint=terms&depth=0&query=GO:0043067) regulation of programmed cell death | 154.558 | 191 | 0.00865439 |
| Gene Ontology | [visit](http://www.godatabase.org/cgi-bin/amigo/go.cgi?view=details&search_constraint=terms&depth=0&query=GO:0035091) phosphoinositide binding | 19.8151 | 34 | 0.00879483 |
| Gene Ontology | [visit](http://www.godatabase.org/cgi-bin/amigo/go.cgi?view=details&search_constraint=terms&depth=0&query=GO:0060512) prostate gland morphogenesis | 5.16916 | 13 | 0.00879483 |
| Gene Ontology | [visit](http://www.godatabase.org/cgi-bin/amigo/go.cgi?view=details&search_constraint=terms&depth=0&query=GO:0006911) phagocytosis, engulfment | 1.89536 | 7 | 0.00908087 |
| Gene Ontology | [visit](http://www.godatabase.org/cgi-bin/amigo/go.cgi?view=details&search_constraint=terms&depth=0&query=GO:0035326) enhancer binding | 7.06452 | 16 | 0.00908087 |
| Gene Ontology | [visit](http://www.godatabase.org/cgi-bin/amigo/go.cgi?view=details&search_constraint=terms&depth=0&query=GO:0060525) prostate glandular acinus development | 1.89536 | 7 | 0.00908087 |
| Gene Ontology | [visit](http://www.godatabase.org/cgi-bin/amigo/go.cgi?view=details&search_constraint=terms&depth=0&query=GO:0070412) R-SMAD binding | 1.89536 | 7 | 0.00908087 |
| Gene Ontology | [visit](http://www.godatabase.org/cgi-bin/amigo/go.cgi?view=details&search_constraint=terms&depth=0&query=GO:0016879) ligase activity, forming carbon-nitrogen bonds | 47.0393 | 68 | 0.00912864 |
| Gene Ontology | [visit](http://www.godatabase.org/cgi-bin/amigo/go.cgi?view=details&search_constraint=terms&depth=0&query=GO:0030055) cell-substrate junction | 16.8859 | 30 | 0.00926582 |
| Gene Ontology | [visit](http://www.godatabase.org/cgi-bin/amigo/go.cgi?view=details&search_constraint=terms&depth=0&query=GO:0031988) membrane-bounded vesicle | 115.445 | 147 | 0.0095282 |
| Gene Ontology | [visit](http://www.godatabase.org/cgi-bin/amigo/go.cgi?view=details&search_constraint=terms&depth=0&query=GO:0005275) amine transmembrane  transporter activity | 12.5783 | 24 | 0.00979686 |
| Gene Ontology | [visit](http://www.godatabase.org/cgi-bin/amigo/go.cgi?view=details&search_constraint=terms&depth=0&query=GO:0030518) steroid hormone receptor signaling pathway | 12.5783 | 24 | 0.00979686 |
| Gene Ontology | [visit](http://www.godatabase.org/cgi-bin/amigo/go.cgi?view=details&search_constraint=terms&depth=0&query=GO:0048562) embryonic organ morphogenesis | 23.7781 | 39 | 0.00979686 |
| Gene Ontology | [visit](http://www.godatabase.org/cgi-bin/amigo/go.cgi?view=details&search_constraint=terms&depth=0&query=GO:0060324) face development | 2.92919 | 9 | 0.00981438 |
| Gene Ontology | [visit](http://www.godatabase.org/cgi-bin/amigo/go.cgi?view=details&search_constraint=terms&depth=0&query=GO:0003707) steroid hormone receptor activity | 8.44296 | 18 | 0.00993535 |
| Gene Ontology | [visit](http://www.godatabase.org/cgi-bin/amigo/go.cgi?view=details&search_constraint=terms&depth=0&query=GO:0030234) enzyme regulator activity | 150.595 | 186 | 0.0100463 |
| Gene Ontology | [visit](http://www.godatabase.org/cgi-bin/amigo/go.cgi?view=details&search_constraint=terms&depth=0&query=GO:0000164) protein phosphatase type 1 complex | 0.689221 | 4 | 0.0100925 |
| Gene Ontology | [visit](http://www.godatabase.org/cgi-bin/amigo/go.cgi?view=details&search_constraint=terms&depth=0&query=GO:0022603) regulation of anatomical structure morphogenesis | 53.9315 | 76 | 0.0100925 |
| Gene Ontology | [visit](http://www.godatabase.org/cgi-bin/amigo/go.cgi?view=details&search_constraint=terms&depth=0&query=GO:0001889) liver development | 11.1998 | 22 | 0.010109 |
| Gene Ontology | [visit](http://www.godatabase.org/cgi-bin/amigo/go.cgi?view=details&search_constraint=terms&depth=0&query=GO:0031982) vesicle | 123.715 | 156 | 0.0102324 |
| Gene Ontology | [visit](http://www.godatabase.org/cgi-bin/amigo/go.cgi?view=details&search_constraint=terms&depth=0&query=GO:0021954) central nervous system neuron development | 4.65224 | 12 | 0.0102431 |
| Gene Ontology | [visit](http://www.godatabase.org/cgi-bin/amigo/go.cgi?view=details&search_constraint=terms&depth=0&query=GO:0051239) regulation of ulticellular organismal process | 187.124 | 226 | 0.0104128 |
| Gene Ontology | [visit](http://www.godatabase.org/cgi-bin/amigo/go.cgi?view=details&search_constraint=terms&depth=0&query=GO:0006913) nucleocytoplasmic transport | 38.2518 | 57 | 0.0104905 |
| Gene Ontology | [visit](http://www.godatabase.org/cgi-bin/amigo/go.cgi?view=details&search_constraint=terms&depth=0&query=GO:0022891) substrate-specific transmembrane transporter activity | 141.807 | 176 | 0.0106772 |
| Gene Ontology | [visit](http://www.godatabase.org/cgi-bin/amigo/go.cgi?view=details&search_constraint=terms&depth=0&query=GO:0006470) protein amino acid dephosphorylation | 23.9504 | 39 | 0.0110642 |
| Gene Ontology | [visit](http://www.godatabase.org/cgi-bin/amigo/go.cgi?view=details&search_constraint=terms&depth=0&query=GO:0006811) ion transport | 142.841 | 177 | 0.0111146 |
| Gene Ontology | [visit](http://www.godatabase.org/cgi-bin/amigo/go.cgi?view=details&search_constraint=terms&depth=0&query=GO:0008283) cell proliferation | 201.253 | 241 | 0.0114837 |
| Gene Ontology | [visit](http://www.godatabase.org/cgi-bin/amigo/go.cgi?view=details&search_constraint=terms&depth=0&query=GO:0051169) nuclear transport | 38.4241 | 57 | 0.0116458 |
| Gene Ontology | [visit](http://www.godatabase.org/cgi-bin/amigo/go.cgi?view=details&search_constraint=terms&depth=0&query=GO:0005626) insoluble fraction | 143.013 | 177 | 0.0116966 |
| Gene Ontology | [visit](http://www.godatabase.org/cgi-bin/amigo/go.cgi?view=details&search_constraint=terms&depth=0&query=GO:0051493) regulation of cytoskeleton organization | 24.812 | 40 | 0.0117399 |
| Gene Ontology | [visit](http://www.godatabase.org/cgi-bin/amigo/go.cgi?view=details&search_constraint=terms&depth=0&query=GO:0006469) negative regulation of protein kinase activity | 17.9197 | 31 | 0.0119339 |
| Gene Ontology | [visit](http://www.godatabase.org/cgi-bin/amigo/go.cgi?view=details&search_constraint=terms&depth=0&query=GO:0005746) mitochondrial respiratory chain | 12.0614 | 3 | 0.0119472 |
| Gene Ontology | [visit](http://www.godatabase.org/cgi-bin/amigo/go.cgi?view=details&search_constraint=terms&depth=0&query=GO:0007034) vacuolar transport | 5.34146 | 13 | 0.0119472 |
| Gene Ontology | [visit](http://www.godatabase.org/cgi-bin/amigo/go.cgi?view=details&search_constraint=terms&depth=0&query=GO:0033261) regulation of S phase | 4.13533 | 11 | 0.0119472 |
| Gene Ontology | [visit](http://www.godatabase.org/cgi-bin/amigo/go.cgi?view=details&search_constraint=terms&depth=0&query=GO:0048471) perinuclear region of cytoplasm | 56.8607 | 79 | 0.0120716 |
| Gene Ontology | [visit](http://www.godatabase.org/cgi-bin/amigo/go.cgi?view=details&search_constraint=terms&depth=0&query=GO:0007173) epidermal growth factor receptor signaling pathway | 7.92604 | 17 | 0.012127 |
| Gene Ontology | [visit](http://www.godatabase.org/cgi-bin/amigo/go.cgi?view=details&search_constraint=terms&depth=0&query=GO:0061138) morphogenesis of a branching epithelium | 19.4705 | 33 | 0.012231 |
| Gene Ontology | [visit](http://www.godatabase.org/cgi-bin/amigo/go.cgi?view=details&search_constraint=terms&depth=0&query=GO:0042981) regulation of apoptosis | 153.179 | 188 | 0.0123209 |
| Gene Ontology | [visit](http://www.godatabase.org/cgi-bin/amigo/go.cgi?view=details&search_constraint=terms&depth=0&query=GO:0022857) transmembrane transporter activity | 155.936 | 191 | 0.0124375 |
| Gene Ontology | [visit](http://www.godatabase.org/cgi-bin/amigo/go.cgi?view=details&search_constraint=terms&depth=0&query=GO:0008286) insulin receptor signaling pathway | 9.30448 | 19 | 0.0125392 |
| Gene Ontology | [visit](http://www.godatabase.org/cgi-bin/amigo/go.cgi?view=details&search_constraint=terms&depth=0&query=GO:0044429) mitochondrial part | 106.657 | 79 | 0.0129841 |
| Gene Ontology | [visit](http://www.godatabase.org/cgi-bin/amigo/go.cgi?view=details&search_constraint=terms&depth=0&query=GO:0042692) muscle cell differentiation | 24.9843 | 40 | 0.0132264 |
| Gene Ontology | [visit](http://www.godatabase.org/cgi-bin/amigo/go.cgi?view=details&search_constraint=terms&depth=0&query=GO:0006816) calcium ion transport | 33.7718 | 51 | 0.0132332 |
| Gene Ontology | [visit](http://www.godatabase.org/cgi-bin/amigo/go.cgi?view=details&search_constraint=terms&depth=0&query=GO:0051726) regulation of cell cycle | 77.7097 | 103 | 0.0132332 |
| Gene Ontology | [visit](http://www.godatabase.org/cgi-bin/amigo/go.cgi?view=details&search_constraint=terms&depth=0&query=GO:0043025) neuronal cell body | 31.3596 | 48 | 0.0132582 |
| Gene Ontology | [visit](http://www.godatabase.org/cgi-bin/amigo/go.cgi?view=details&search_constraint=terms&depth=0&query=GO:0044297) cell body | 31.3596 | 48 | 0.0132582 |
| Gene Ontology | [visit](http://www.godatabase.org/cgi-bin/amigo/go.cgi?view=details&search_constraint=terms&depth=0&query=GO:0004672) protein kinase activity | 102.349 | 131 | 0.0134352 |
| Gene Ontology | [visit](http://www.godatabase.org/cgi-bin/amigo/go.cgi?view=details&search_constraint=terms&depth=0&query=GO:0031102) neuron projection regeneration | 3.61841 | 10 | 0.013581 |
| Gene Ontology | [visit](http://www.godatabase.org/cgi-bin/amigo/go.cgi?view=details&search_constraint=terms&depth=0&query=GO:0016023) cytoplasmic membranebounded vesicle | 112.171 | 142 | 0.0136188 |
| Gene Ontology | [visit](http://www.godatabase.org/cgi-bin/amigo/go.cgi?view=details&search_constraint=terms&depth=0&query=GO:0003705) RNA polymerase II transcription factor activity, enhancer binding | 6.71991 | 15 | 0.0141853 |
| Gene Ontology | [visit](http://www.godatabase.org/cgi-bin/amigo/go.cgi?view=details&search_constraint=terms&depth=0&query=GO:0014706) striated muscle tissue development | 28.2581 | 44 | 0.0141853 |
| Gene Ontology | [visit](http://www.godatabase.org/cgi-bin/amigo/go.cgi?view=details&search_constraint=terms&depth=0&query=GO:0055037) recycling endosome | 6.71991 | 15 | 0.0141853 |
| Gene Ontology | [visit](http://www.godatabase.org/cgi-bin/amigo/go.cgi?view=details&search_constraint=terms&depth=0&query=GO:0005388) calcium-transporting AT-  Pase activity | 1.55075 | 6 | 0.01445 |
| Gene Ontology | [visit](http://www.godatabase.org/cgi-bin/amigo/go.cgi?view=details&search_constraint=terms&depth=0&query=GO:0007440) foregut morphogenesis | 1.55075 | 6 | 0.01445 |
| Gene Ontology | [visit](http://www.godatabase.org/cgi-bin/amigo/go.cgi?view=details&search_constraint=terms&depth=0&query=GO:0030879) mammary gland development | 15.1629 | 27 | 0.01445 |
| Gene Ontology | [visit](http://www.godatabase.org/cgi-bin/amigo/go.cgi?view=details&search_constraint=terms&depth=0&query=GO:0043068) positive regulation of programmed cell death | 80.6389 | 106 | 0.0148314 |
| Gene Ontology | [visit](http://www.godatabase.org/cgi-bin/amigo/go.cgi?view=details&search_constraint=terms&depth=0&query=GO:0070507) regulation of microtubule cytoskeleton organization | 7.40913 | 16 | 0.0148314 |
| Gene Ontology | [visit](http://www.godatabase.org/cgi-bin/amigo/go.cgi?view=details&search_constraint=terms&depth=0&query=GO:0006606) protein import into nucleus | 23.6058 | 38 | 0.0150025 |
| Gene Ontology | [visit](http://www.godatabase.org/cgi-bin/amigo/go.cgi?view=details&search_constraint=terms&depth=0&query=GO:0060828) regulation of canonical Wnt receptor signaling pathway | 8.09835 | 17 | 0.0152548 |
| Gene Ontology | [visit](http://www.godatabase.org/cgi-bin/amigo/go.cgi?view=details&search_constraint=terms&depth=0&query=GO:0008645) hexose transport | 10.166 | 20 | 0.0152769 |
| Gene Ontology | [visit](http://www.godatabase.org/cgi-bin/amigo/go.cgi?view=details&search_constraint=terms&depth=0&query=GO:0015758) glucose transport | 10.166 | 20 | 0.0152769 |
| Gene Ontology | [visit](http://www.godatabase.org/cgi-bin/amigo/go.cgi?view=details&search_constraint=terms&depth=0&query=GO:0030163) protein catabolic process | 68.5775 | 92 | 0.0153445 |
| Gene Ontology | [visit](http://www.godatabase.org/cgi-bin/amigo/go.cgi?view=details&search_constraint=terms&depth=0&query=GO:0000165) MAPKKK cascade | 51.5193 | 72 | 0.0156324 |
| Gene Ontology | [visit](http://www.godatabase.org/cgi-bin/amigo/go.cgi?view=details&search_constraint=terms&depth=0&query=GO:0034660) ncRNA metabolic process | 40.664 | 24 | 0.0158531 |
| Gene Ontology | [visit](http://www.godatabase.org/cgi-bin/amigo/go.cgi?view=details&search_constraint=terms&depth=0&query=GO:0046324) regulation of glucose import | 5.51377 | 13 | 0.0159113 |
| Gene Ontology | [visit](http://www.godatabase.org/cgi-bin/amigo/go.cgi?view=details&search_constraint=terms&depth=0&query=GO:0048066) developmental pigmentation | 5.51377 | 13 | 0.0159113 |
| Gene Ontology | [visit](http://www.godatabase.org/cgi-bin/amigo/go.cgi?view=details&search_constraint=terms&depth=0&query=GO:0070469) respiratory chain | 13.2675 | 4 | 0.0161666 |
| Gene Ontology | [visit](http://www.godatabase.org/cgi-bin/amigo/go.cgi?view=details&search_constraint=terms&depth=0&query=GO:0007164) establishment of tissue polarity | 2.06766 | 7 | 0.0163057 |
| Gene Ontology | [visit](http://www.godatabase.org/cgi-bin/amigo/go.cgi?view=details&search_constraint=terms&depth=0&query=GO:0032403) protein complex binding | 42.3871 | 61 | 0.0163057 |
| Gene Ontology | [visit](http://www.godatabase.org/cgi-bin/amigo/go.cgi?view=details&search_constraint=terms&depth=0&query=GO:0070838) divalent metal ion transport | 34.978 | 52 | 0.0164203 |
| Gene Ontology | [visit](http://www.godatabase.org/cgi-bin/amigo/go.cgi?view=details&search_constraint=terms&depth=0&query=GO:0045664) regulation of neuron differentiation | 31.0149 | 47 | 0.0176011 |
| Gene Ontology | [visit](http://www.godatabase.org/cgi-bin/amigo/go.cgi?view=details&search_constraint=terms&depth=0&query=GO:0030427) site of polarized growth | 11.0275 | 21 | 0.0183364 |
| Gene Ontology | [visit](http://www.godatabase.org/cgi-bin/amigo/go.cgi?view=details&search_constraint=terms&depth=0&query=GO:0019866) organelle inner membrane | 57.55 | 38 | 0.018437 |
| Gene Ontology | [visit](http://www.godatabase.org/cgi-bin/amigo/go.cgi?view=details&search_constraint=terms&depth=0&query=GO:0031410) cytoplasmic vesicle | 118.546 | 148 | 0.0185998 |
| Gene Ontology | [visit](http://www.godatabase.org/cgi-bin/amigo/go.cgi?view=details&search_constraint=terms&depth=0&query=GO:0015749) monosaccharide transport | 10.3383 | 20 | 0.0187383 |
| Gene Ontology | [visit](http://www.godatabase.org/cgi-bin/amigo/go.cgi?view=details&search_constraint=terms&depth=0&query=GO:0045216) cell-cell junction organization | 7.58143 | 16 | 0.0188717 |
| Gene Ontology | [visit](http://www.godatabase.org/cgi-bin/amigo/go.cgi?view=details&search_constraint=terms&depth=0&query=GO:0060021) palate development | 7.58143 | 16 | 0.0188717 |
| Gene Ontology | [visit](http://www.godatabase.org/cgi-bin/amigo/go.cgi?view=details&search_constraint=terms&depth=0&query=GO:0032535) regulation of cellular component size | 60.4792 | 82 | 0.0190544 |
| Gene Ontology | [visit](http://www.godatabase.org/cgi-bin/amigo/go.cgi?view=details&search_constraint=terms&depth=0&query=GO:0042472) inner ear morphogenesis | 8.95987 | 18 | 0.0191539 |
| Gene Ontology | [visit](http://www.godatabase.org/cgi-bin/amigo/go.cgi?view=details&search_constraint=terms&depth=0&query=GO:0018210) peptidyl-threonine modification | 4.99685 | 12 | 0.0193533 |
| Gene Ontology | [visit](http://www.godatabase.org/cgi-bin/amigo/go.cgi?view=details&search_constraint=terms&depth=0&query=GO:0050767) regulation of neurogenesis | 37.7349 | 55 | 0.0194313 |
| Gene Ontology | [visit](http://www.godatabase.org/cgi-bin/amigo/go.cgi?view=details&search_constraint=terms&depth=0&query=GO:0060348) bone development | 34.4611 | 51 | 0.0196169 |
| Gene Ontology | [visit](http://www.godatabase.org/cgi-bin/amigo/go.cgi?view=details&search_constraint=terms&depth=0&query=GO:0030326) embryonic limb morpho-  genesis | 15.5075 | 27 | 0.0196533 |
| Gene Ontology | [visit](http://www.godatabase.org/cgi-bin/amigo/go.cgi?view=details&search_constraint=terms&depth=0&query=GO:0035113) embryonic appendage morphogenesis | 15.5075 | 27 | 0.0196533 |
| Gene Ontology | [visit](http://www.godatabase.org/cgi-bin/amigo/go.cgi?view=details&search_constraint=terms&depth=0&query=GO:0060491) regulation of cell projection assembly | 3.79072 | 10 | 0.0196533 |
| Gene Ontology | [visit](http://www.godatabase.org/cgi-bin/amigo/go.cgi?view=details&search_constraint=terms&depth=0&query=GO:0030099) myeloid cell differentiation | 27.2242 | 42 | 0.0203121 |
| Gene Ontology | [visit](http://www.godatabase.org/cgi-bin/amigo/go.cgi?view=details&search_constraint=terms&depth=0&query=GO:0043566) structure-specific DNA binding | 25.6735 | 40 | 0.0210229 |
| Gene Ontology | [visit](http://www.godatabase.org/cgi-bin/amigo/go.cgi?view=details&search_constraint=terms&depth=0&query=GO:0005856) cytoskeleton | 242.95 | 283 | 0.0210941 |
| Gene Ontology | [visit](http://www.godatabase.org/cgi-bin/amigo/go.cgi?view=details&search_constraint=terms&depth=0&query=GO:0031625) ubiquitin protein ligase binding | 11.8891 | 22 | 0.0211547 |
| Gene Ontology | [visit](http://www.godatabase.org/cgi-bin/amigo/go.cgi?view=details&search_constraint=terms&depth=0&query=GO:0016072) rRNA metabolic process | 17.4028 | 7 | 0.0213926 |
| Gene Ontology | [visit](http://www.godatabase.org/cgi-bin/amigo/go.cgi?view=details&search_constraint=terms&depth=0&query=GO:0006812) cation transport | 101.143 | 128 | 0.0213989 |
| Gene Ontology | [visit](http://www.godatabase.org/cgi-bin/amigo/go.cgi?view=details&search_constraint=terms&depth=0&query=GO:0015630) microtubule cytoskeleton | 101.143 | 128 | 0.0213989 |
| Gene Ontology | [visit](http://www.godatabase.org/cgi-bin/amigo/go.cgi?view=details&search_constraint=terms&depth=0&query=GO:0051170) nuclear import | 24.1227 | 38 | 0.0214283 |
| Gene Ontology | [visit](http://www.godatabase.org/cgi-bin/amigo/go.cgi?view=details&search_constraint=terms&depth=0&query=GO:0051216) cartilage development | 16.369 | 28 | 0.0215512 |
| Gene Ontology | [visit](http://www.godatabase.org/cgi-bin/amigo/go.cgi?view=details&search_constraint=terms&depth=0&query=GO:0019199) transmembrane receptor protein kinase activity | 14.129 | 25 | 0.0217454 |
| Gene Ontology | [visit](http://www.godatabase.org/cgi-bin/amigo/go.cgi?view=details&search_constraint=terms&depth=0&query=GO:0034330) cell junction organization | 14.129 | 25 | 0.0217454 |
| Gene Ontology | [visit](http://www.godatabase.org/cgi-bin/amigo/go.cgi?view=details&search_constraint=terms&depth=0&query=GO:0031344) regulation of cell projection organization | 22.572 | 36 | 0.0217705 |
| Gene Ontology | [visit](http://www.godatabase.org/cgi-bin/amigo/go.cgi?view=details&search_constraint=terms&depth=0&query=GO:0015992) proton transport | 11.1998 | 3 | 0.0220613 |
| Gene Ontology | [visit](http://www.godatabase.org/cgi-bin/amigo/go.cgi?view=details&search_constraint=terms&depth=0&query=GO:0055114) oxidation reduction | 111.482 | 85 | 0.0223158 |
| Gene Ontology | [visit](http://www.godatabase.org/cgi-bin/amigo/go.cgi?view=details&search_constraint=terms&depth=0&query=GO:0000159) protein phosphatase type  2A complex | 3.2738 | 9 | 0.0225259 |
| Gene Ontology | [visit](http://www.godatabase.org/cgi-bin/amigo/go.cgi?view=details&search_constraint=terms&depth=0&query=GO:0030032) lamellipodium assembly | 3.2738 | 9 | 0.0225259 |
| Gene Ontology | [visit](http://www.godatabase.org/cgi-bin/amigo/go.cgi?view=details&search_constraint=terms&depth=0&query=GO:0007158) neuron cell-cell adhesion | 1.20614 | 5 | 0.022577 |
| Gene Ontology | [visit](http://www.godatabase.org/cgi-bin/amigo/go.cgi?view=details&search_constraint=terms&depth=0&query=GO:0050434) positive regulation of viral transcription | 1.20614 | 5 | 0.022577 |
| Gene Ontology | [visit](http://www.godatabase.org/cgi-bin/amigo/go.cgi?view=details&search_constraint=terms&depth=0&query=GO:0090003) regulation of establishment of protein localization in plasma membrane | 1.20614 | 5 | 0.022577 |
| Gene Ontology | [visit](http://www.godatabase.org/cgi-bin/amigo/go.cgi?view=details&search_constraint=terms&depth=0&query=GO:0043065) positive regulation of apoptosis | 80.122 | 104 | 0.0228256 |
| Gene Ontology | [visit](http://www.godatabase.org/cgi-bin/amigo/go.cgi?view=details&search_constraint=terms&depth=0&query=GO:0043491) protein kinase B signaling cascade | 7.06452 | 15 | 0.0229177 |
| Gene Ontology | [visit](http://www.godatabase.org/cgi-bin/amigo/go.cgi?view=details&search_constraint=terms&depth=0&query=GO:0007162) negative regulation of cell adhesion | 9.13218 | 18 | 0.0230342 |
| Gene Ontology | [visit](http://www.godatabase.org/cgi-bin/amigo/go.cgi?view=details&search_constraint=terms&depth=0&query=GO:0015662) ATPase activity, coupled to transmembrane movement of ions, phosphorylative mechanism | 9.13218 | 18 | 0.0230342 |
| Gene Ontology | [visit](http://www.godatabase.org/cgi-bin/amigo/go.cgi?view=details&search_constraint=terms&depth=0&query=GO:0022613) ribonucleoprotein complex biogenesis | 33.4272 | 19 | 0.0230342 |
| Gene Ontology | [visit](http://www.godatabase.org/cgi-bin/amigo/go.cgi?view=details&search_constraint=terms&depth=0&query=GO:0031256) leading edge membrane | 9.13218 | 18 | 0.0230342 |
| Gene Ontology | [visit](http://www.godatabase.org/cgi-bin/amigo/go.cgi?view=details&search_constraint=terms&depth=0&query=GO:0070997) neuron death | 17.2305 | 29 | 0.0230342 |
| Gene Ontology | [visit](http://www.godatabase.org/cgi-bin/amigo/go.cgi?view=details&search_constraint=terms&depth=0&query=GO:0033673) negative regulation of kinase activity | 18.7813 | 31 | 0.0232862 |
| Gene Ontology | [visit](http://www.godatabase.org/cgi-bin/amigo/go.cgi?view=details&search_constraint=terms&depth=0&query=GO:0001503) ossification | 31.5319 | 47 | 0.0233064 |
| Gene Ontology | [visit](http://www.godatabase.org/cgi-bin/amigo/go.cgi?view=details&search_constraint=terms&depth=0&query=GO:0005925) focal adhesion | 14.9906 | 26 | 0.0236749 |
| Gene Ontology | [visit](http://www.godatabase.org/cgi-bin/amigo/go.cgi?view=details&search_constraint=terms&depth=0&query=GO:0022612) gland morphogenesis | 14.9906 | 26 | 0.0236749 |
| Gene Ontology | [visit](http://www.godatabase.org/cgi-bin/amigo/go.cgi?view=details&search_constraint=terms&depth=0&query=GO:0050877) neurological system process | 219.172 | 183 | 0.0241605 |
| Gene Ontology | [visit](http://www.godatabase.org/cgi-bin/amigo/go.cgi?view=details&search_constraint=terms&depth=0&query=GO:0005624) membrane fraction | 137.672 | 168 | 0.024323 |
| Gene Ontology | [visit](http://www.godatabase.org/cgi-bin/amigo/go.cgi?view=details&search_constraint=terms&depth=0&query=GO:0043062) extracellular structure organization | 29.9811 | 45 | 0.0243858 |
| Gene Ontology | [visit](http://www.godatabase.org/cgi-bin/amigo/go.cgi?view=details&search_constraint=terms&depth=0&query=GO:0008022) protein C-terminus binding | 25.1566 | 39 | 0.0246081 |
| Gene Ontology | [visit](http://www.godatabase.org/cgi-bin/amigo/go.cgi?view=details&search_constraint=terms&depth=0&query=GO:0022892) substrate-specific transporter activity | 166.102 | 199 | 0.0249269 |
| Gene Ontology | [visit](http://www.godatabase.org/cgi-bin/amigo/go.cgi?view=details&search_constraint=terms&depth=0&query=GO:0030947) regulation of vascular endothelial growth factor receptor signaling pathway | 2.75688 | 8 | 0.0249269 |
| Gene Ontology | [visit](http://www.godatabase.org/cgi-bin/amigo/go.cgi?view=details&search_constraint=terms&depth=0&query=GO:0032330) regulation of chondrocyte differentiation | 2.75688 | 8 | 0.0249269 |
| Gene Ontology | [visit](http://www.godatabase.org/cgi-bin/amigo/go.cgi?view=details&search_constraint=terms&depth=0&query=GO:0043209) myelin sheath | 2.75688 | 8 | 0.0249269 |
| Gene Ontology | [visit](http://www.godatabase.org/cgi-bin/amigo/go.cgi?view=details&search_constraint=terms&depth=0&query=GO:0048806) genitalia development | 5.16916 | 12 | 0.0251403 |
| Gene Ontology | [visit](http://www.godatabase.org/cgi-bin/amigo/go.cgi?view=details&search_constraint=terms&depth=0&query=GO:0005924) cell-substrate adherens junction | 15.8521 | 27 | 0.0258659 |
| Gene Ontology | [visit](http://www.godatabase.org/cgi-bin/amigo/go.cgi?view=details&search_constraint=terms&depth=0&query=GO:0005083) small GTPase regulator activity | 50.1408 | 69 | 0.0259311 |
| Gene Ontology | [visit](http://www.godatabase.org/cgi-bin/amigo/go.cgi?view=details&search_constraint=terms&depth=0&query=GO:0048608) reproductive structure development | 29.2919 | 44 | 0.0261611 |
| Gene Ontology | [visit](http://www.godatabase.org/cgi-bin/amigo/go.cgi?view=details&search_constraint=terms&depth=0&query=GO:0033043) regulation of organelle organization | 42.5594 | 60 | 0.0263981 |
| Gene Ontology | [visit](http://www.godatabase.org/cgi-bin/amigo/go.cgi?view=details&search_constraint=terms&depth=0&query=GO:0004115) 3’,5’-cyclic-AMP phosphodiesterase activity | 1.72305 | 6 | 0.0267338 |
| Gene Ontology | [visit](http://www.godatabase.org/cgi-bin/amigo/go.cgi?view=details&search_constraint=terms&depth=0&query=GO:0021952) central nervous system projection neuron axonogenesis | 1.72305 | 6 | 0.0267338 |
| Gene Ontology | [visit](http://www.godatabase.org/cgi-bin/amigo/go.cgi?view=details&search_constraint=terms&depth=0&query=GO:0032154) cleavage furrow | 1.72305 | 6 | 0.0267338 |
| Gene Ontology | [visit](http://www.godatabase.org/cgi-bin/amigo/go.cgi?view=details&search_constraint=terms&depth=0&query=GO:0021955) central nervous system neuron axonogenesis | 2.23997 | 7 | 0.0267657 |
| Gene Ontology | [visit](http://www.godatabase.org/cgi-bin/amigo/go.cgi?view=details&search_constraint=terms&depth=0&query=GO:0048678) response to axon injury | 5.85838 | 13 | 0.0267657 |
| Gene Ontology | [visit](http://www.godatabase.org/cgi-bin/amigo/go.cgi?view=details&search_constraint=terms&depth=0&query=GO:0032583) regulation of gene-specific transcription | 38.4241 | 55 | 0.026952 |
| Gene Ontology | [visit](http://www.godatabase.org/cgi-bin/amigo/go.cgi?view=details&search_constraint=terms&depth=0&query=GO:0043193) positive regulation of genespecific transcription | 25.3289 | 39 | 0.0272593 |
| Gene Ontology | [visit](http://www.godatabase.org/cgi-bin/amigo/go.cgi?view=details&search_constraint=terms&depth=0&query=GO:0017038) protein import | 31.0149 | 46 | 0.027412 |
| Gene Ontology | [visit](http://www.godatabase.org/cgi-bin/amigo/go.cgi?view=details&search_constraint=terms&depth=0&query=GO:0005739) mitochondrion | 219.517 | 184 | 0.0275581 |
| Gene Ontology | [visit](http://www.godatabase.org/cgi-bin/amigo/go.cgi?view=details&search_constraint=terms&depth=0&query=GO:0009755) hormone-mediated signaling pathway | 9.30448 | 18 | 0.0276758 |
| Gene Ontology | [visit](http://www.godatabase.org/cgi-bin/amigo/go.cgi?view=details&search_constraint=terms&depth=0&query=GO:0051960) regulation of nervous system development | 41.0087 | 58 | 0.0278959 |
| Gene Ontology | [visit](http://www.godatabase.org/cgi-bin/amigo/go.cgi?view=details&search_constraint=terms&depth=0&query=GO:0044265) cellular macromolecule catabolic process | 82.5342 | 106 | 0.027915 |
| Gene Ontology | [visit](http://www.godatabase.org/cgi-bin/amigo/go.cgi?view=details&search_constraint=terms&depth=0&query=GO:0033267) axon part | 12.2337 | 22 | 0.028738 |
| Gene Ontology | [visit](http://www.godatabase.org/cgi-bin/amigo/go.cgi?view=details&search_constraint=terms&depth=0&query=GO:0042254) ribosome biogenesis | 22.3997 | 11 | 0.0291634 |
| Gene Ontology | [visit](http://www.godatabase.org/cgi-bin/amigo/go.cgi?view=details&search_constraint=terms&depth=0&query=GO:0022843) voltage-gated cation channel activity | 24.6397 | 38 | 0.0292674 |
| Gene Ontology | [visit](http://www.godatabase.org/cgi-bin/amigo/go.cgi?view=details&search_constraint=terms&depth=0&query=GO:0060429) epithelium development | 64.2699 | 85 | 0.0296732 |
| Gene Ontology | [visit](http://www.godatabase.org/cgi-bin/amigo/go.cgi?view=details&search_constraint=terms&depth=0&query=GO:0019838) growth factor binding | 19.1259 | 31 | 0.0298279 |
| Gene Ontology | [visit](http://www.godatabase.org/cgi-bin/amigo/go.cgi?view=details&search_constraint=terms&depth=0&query=GO:0004725) protein tyrosine phosphatase activity | 17.5751 | 29 | 0.0298543 |
| Gene Ontology | [visit](http://www.godatabase.org/cgi-bin/amigo/go.cgi?view=details&search_constraint=terms&depth=0&query=GO:0031346) positive regulation of cell projection organization | 11.5445 | 21 | 0.0299749 |
| Gene Ontology | [visit](http://www.godatabase.org/cgi-bin/amigo/go.cgi?view=details&search_constraint=terms&depth=0&query=GO:0044419) interspecies interaction between organisms | 59.1007 | 79 | 0.029984 |
| Gene Ontology | [visit](http://www.godatabase.org/cgi-bin/amigo/go.cgi?view=details&search_constraint=terms&depth=0&query=GO:0007041) lysosomal transport | 4.65224 | 11 | 0.0301872 |
| Gene Ontology | [visit](http://www.godatabase.org/cgi-bin/amigo/go.cgi?view=details&search_constraint=terms&depth=0&query=GO:0014812) muscle cell migration | 4.65224 | 11 | 0.0301872 |
| Gene Ontology | [visit](http://www.godatabase.org/cgi-bin/amigo/go.cgi?view=details&search_constraint=terms&depth=0&query=GO:0030027) lamellipodium | 13.7844 | 24 | 0.0301872 |
| Gene Ontology | [visit](http://www.godatabase.org/cgi-bin/amigo/go.cgi?view=details&search_constraint=terms&depth=0&query=GO:0042169) SH2 domain binding | 4.65224 | 11 | 0.0301872 |
| Gene Ontology | [visit](http://www.godatabase.org/cgi-bin/amigo/go.cgi?view=details&search_constraint=terms&depth=0&query=GO:0043066) negative regulation of apoptosis | 69.6113 | 91 | 0.0305863 |
| Gene Ontology | [visit](http://www.godatabase.org/cgi-bin/amigo/go.cgi?view=details&search_constraint=terms&depth=0&query=GO:0006364) rRNA processing | 16.7136 | 7 | 0.0309321 |
| Gene Ontology | [visit](http://www.godatabase.org/cgi-bin/amigo/go.cgi?view=details&search_constraint=terms&depth=0&query=GO:0016772) transferase activity, transferring phosphorus-containing groups | 154.213 | 185 | 0.0309321 |
| Gene Ontology | [visit](http://www.godatabase.org/cgi-bin/amigo/go.cgi?view=details&search_constraint=terms&depth=0&query=GO:0030426) growth cone | 10.8552 | 20 | 0.0309321 |
| Gene Ontology | [visit](http://www.godatabase.org/cgi-bin/amigo/go.cgi?view=details&search_constraint=terms&depth=0&query=GO:0035282) segmentation | 10.8552 | 20 | 0.0309321 |
| Gene Ontology | [visit](http://www.godatabase.org/cgi-bin/amigo/go.cgi?view=details&search_constraint=terms&depth=0&query=GO:0010720) positive regulation of cell development | 15.3352 | 26 | 0.0310333 |
| Gene Ontology | [visit](http://www.godatabase.org/cgi-bin/amigo/go.cgi?view=details&search_constraint=terms&depth=0&query=GO:0007062) sister chromatid cohesion | 3.44611 | 9 | 0.0312976 |
| Gene Ontology | [visit](http://www.godatabase.org/cgi-bin/amigo/go.cgi?view=details&search_constraint=terms&depth=0&query=GO:0010171) body morphogenesis | 3.44611 | 9 | 0.0312976 |
| Gene Ontology | [visit](http://www.godatabase.org/cgi-bin/amigo/go.cgi?view=details&search_constraint=terms&depth=0&query=GO:0042551) neuron maturation | 3.44611 | 9 | 0.0312976 |
| Gene Ontology | [visit](http://www.godatabase.org/cgi-bin/amigo/go.cgi?view=details&search_constraint=terms&depth=0&query=GO:0031966) mitochondrial membrane | 72.3682 | 52 | 0.031326 |
| Gene Ontology | [visit](http://www.godatabase.org/cgi-bin/amigo/go.cgi?view=details&search_constraint=terms&depth=0&query=GO:0001726) ruffle | 13.0952 | 23 | 0.0314415 |
| Gene Ontology | [visit](http://www.godatabase.org/cgi-bin/amigo/go.cgi?view=details&search_constraint=terms&depth=0&query=GO:0004674) protein serinethreonine kinase activity | 73.2297 | 95 | 0.0314415 |
| Gene Ontology | [visit](http://www.godatabase.org/cgi-bin/amigo/go.cgi?view=details&search_constraint=terms&depth=0&query=GO:0048469) cell maturation | 13.0952 | 23 | 0.0314415 |
| Gene Ontology | [visit](http://www.godatabase.org/cgi-bin/amigo/go.cgi?view=details&search_constraint=terms&depth=0&query=GO:0015171) amino acid transmembrane transporter activity | 10.166 | 19 | 0.0317198 |
| Gene Ontology | [visit](http://www.godatabase.org/cgi-bin/amigo/go.cgi?view=details&search_constraint=terms&depth=0&query=GO:0044087) regulation of cellular component biogenesis | 27.2242 | 41 | 0.0317198 |
| Gene Ontology | [visit](http://www.godatabase.org/cgi-bin/amigo/go.cgi?view=details&search_constraint=terms&depth=0&query=GO:0060548) negative regulation of cell death | 71.5067 | 93 | 0.0317198 |
| Gene Ontology | [visit](http://www.godatabase.org/cgi-bin/amigo/go.cgi?view=details&search_constraint=terms&depth=0&query=GO:0042379) chemokine receptor binding | 8.95987 | 2 | 0.0317846 |
| Gene Ontology | [visit](http://www.godatabase.org/cgi-bin/amigo/go.cgi?view=details&search_constraint=terms&depth=0&query=GO:0022803) passive transmembrane transporter activity | 70.6452 | 92 | 0.0317951 |
| Gene Ontology | [visit](http://www.godatabase.org/cgi-bin/amigo/go.cgi?view=details&search_constraint=terms&depth=0&query=GO:0005158) insulin receptor binding | 5.34146 | 12 | 0.0320166 |
| Gene Ontology | [visit](http://www.godatabase.org/cgi-bin/amigo/go.cgi?view=details&search_constraint=terms&depth=0&query=GO:0016301) kinase activity | 132.503 | 161 | 0.0320244 |
| Gene Ontology | [visit](http://www.godatabase.org/cgi-bin/amigo/go.cgi?view=details&search_constraint=terms&depth=0&query=GO:0004652) polynucleotide adenylyltransferase activity | 0.861526 | 4 | 0.0320447 |
| Gene Ontology | [visit](http://www.godatabase.org/cgi-bin/amigo/go.cgi?view=details&search_constraint=terms&depth=0&query=GO:0010761) fibroblast migration | 0.861526 | 4 | 0.0320447 |
| Gene Ontology | [visit](http://www.godatabase.org/cgi-bin/amigo/go.cgi?view=details&search_constraint=terms&depth=0&query=GO:0015106) bicarbonate transmembrane transporter activity | 0.861526 | 4 | 0.0320447 |
| Gene Ontology | [visit](http://www.godatabase.org/cgi-bin/amigo/go.cgi?view=details&search_constraint=terms&depth=0&query=GO:0022614) membrane to membrane docking | 0.861526 | 4 | 0.0320447 |
| Gene Ontology | [visit](http://www.godatabase.org/cgi-bin/amigo/go.cgi?view=details&search_constraint=terms&depth=0&query=GO:0030618) transforming growth factor beta receptor, pathway-specific cytoplasmic mediator activity | 0.861526 | 4 | 0.0320447 |
| Gene Ontology | [visit](http://www.godatabase.org/cgi-bin/amigo/go.cgi?view=details&search_constraint=terms&depth=0&query=GO:0031512) motile primary cilium | 0.861526 | 4 | 0.0320447 |
| Gene Ontology | [visit](http://www.godatabase.org/cgi-bin/amigo/go.cgi?view=details&search_constraint=terms&depth=0&query=GO:0032875) regulation of DNA en-  doreduplication | 0.861526 | 4 | 0.0320447 |
| Gene Ontology | [visit](http://www.godatabase.org/cgi-bin/amigo/go.cgi?view=details&search_constraint=terms&depth=0&query=GO:0032876) negative regulation of DNA endoreduplication | 0.861526 | 4 | 0.0320447 |
| Gene Ontology | [visit](http://www.godatabase.org/cgi-bin/amigo/go.cgi?view=details&search_constraint=terms&depth=0&query=GO:0042023) DNA endoreduplication | 0.861526 | 4 | 0.0320447 |
| Gene Ontology | [visit](http://www.godatabase.org/cgi-bin/amigo/go.cgi?view=details&search_constraint=terms&depth=0&query=GO:0043559) insulin binding | 0.861526 | 4 | 0.0320447 |
| Gene Ontology | [visit](http://www.godatabase.org/cgi-bin/amigo/go.cgi?view=details&search_constraint=terms&depth=0&query=GO:0048087) positive regulation of developmental pigmentation | 0.861526 | 4 | 0.0320447 |
| Gene Ontology | [visit](http://www.godatabase.org/cgi-bin/amigo/go.cgi?view=details&search_constraint=terms&depth=0&query=GO:0010810) regulation of cell-substrate adhesion | 9.47679 | 18 | 0.0320861 |
| Gene Ontology | [visit](http://www.godatabase.org/cgi-bin/amigo/go.cgi?view=details&search_constraint=terms&depth=0&query=GO:0006979) response to oxidative stress | 32.2211 | 47 | 0.0324824 |
| Gene Ontology | [visit](http://www.godatabase.org/cgi-bin/amigo/go.cgi?view=details&search_constraint=terms&depth=0&query=GO:0001952) regulation of cell-matrix adhesion | 6.03068 | 13 | 0.0328778 |
| Gene Ontology | [visit](http://www.godatabase.org/cgi-bin/amigo/go.cgi?view=details&search_constraint=terms&depth=0&query=GO:0010827) regulation of glucose transport | 6.03068 | 13 | 0.0328778 |
| Gene Ontology | [visit](http://www.godatabase.org/cgi-bin/amigo/go.cgi?view=details&search_constraint=terms&depth=0&query=GO:0003006) reproductive developmental process | 51.6916 | 70 | 0.0332626 |
| Gene Ontology | [visit](http://www.godatabase.org/cgi-bin/amigo/go.cgi?view=details&search_constraint=terms&depth=0&query=GO:0060627) regulation of vesiclemediated transport | 21.7105 | 34 | 0.0332963 |
| Gene Ontology | [visit](http://www.godatabase.org/cgi-bin/amigo/go.cgi?view=details&search_constraint=terms&depth=0&query=GO:0030521) androgen receptor signaling pathway | 8.09835 | 16 | 0.0334402 |
| Gene Ontology | [visit](http://www.godatabase.org/cgi-bin/amigo/go.cgi?view=details&search_constraint=terms&depth=0&query=GO:0021537) telencephalon development | 15.5075 | 26 | 0.0346971 |
| Gene Ontology | [visit](http://www.godatabase.org/cgi-bin/amigo/go.cgi?view=details&search_constraint=terms&depth=0&query=GO:0051146) striated muscle cell differentiation | 18.609 | 30 | 0.0346971 |
| Gene Ontology | [visit](http://www.godatabase.org/cgi-bin/amigo/go.cgi?view=details&search_constraint=terms&depth=0&query=GO:0030133) transport vesicle | 17.0582 | 28 | 0.034883 |
| Gene Ontology | [visit](http://www.godatabase.org/cgi-bin/amigo/go.cgi?view=details&search_constraint=terms&depth=0&query=GO:0014909) smooth muscle cell migration | 4.13533 | 10 | 0.0353177 |
| Gene Ontology | [visit](http://www.godatabase.org/cgi-bin/amigo/go.cgi?view=details&search_constraint=terms&depth=0&query=GO:0046326) positive regulation of glucose import | 4.13533 | 10 | 0.0353177 |
| Gene Ontology | [visit](http://www.godatabase.org/cgi-bin/amigo/go.cgi?view=details&search_constraint=terms&depth=0&query=GO:0005215) transporter activity | 202.286 | 236 | 0.0370759 |
| Gene Ontology | [visit](http://www.godatabase.org/cgi-bin/amigo/go.cgi?view=details&search_constraint=terms&depth=0&query=GO:0051301) cell division | 61.513 | 81 | 0.0376476 |
| Gene Ontology | [visit](http://www.godatabase.org/cgi-bin/amigo/go.cgi?view=details&search_constraint=terms&depth=0&query=GO:0015075) ion transmembrane transporter activity | 123.198 | 150 | 0.0380704 |
| Gene Ontology | [visit](http://www.godatabase.org/cgi-bin/amigo/go.cgi?view=details&search_constraint=terms&depth=0&query=GO:0021953) central nervous system neuron differentiation | 12.5783 | 22 | 0.0380704 |
| Gene Ontology | [visit](http://www.godatabase.org/cgi-bin/amigo/go.cgi?view=details&search_constraint=terms&depth=0&query=GO:0005815) microtubule organizing center | 47.7286 | 65 | 0.0384334 |
| Gene Ontology | [visit](http://www.godatabase.org/cgi-bin/amigo/go.cgi?view=details&search_constraint=terms&depth=0&query=GO:0030496) midbody | 4.82455 | 11 | 0.0388796 |
| Gene Ontology | [visit](http://www.godatabase.org/cgi-bin/amigo/go.cgi?view=details&search_constraint=terms&depth=0&query=GO:0048593) camera-type eye morphogenesis | 8.95987 | 17 | 0.0402223 |
| Gene Ontology | [visit](http://www.godatabase.org/cgi-bin/amigo/go.cgi?view=details&search_constraint=terms&depth=0&query=GO:0060090) molecular adaptor activity | 11.8891 | 21 | 0.0402402 |
| Gene Ontology | [visit](http://www.godatabase.org/cgi-bin/amigo/go.cgi?view=details&search_constraint=terms&depth=0&query=GO:0015267) channel activity | 70.4729 | 91 | 0.0403374 |
| Gene Ontology | [visit](http://www.godatabase.org/cgi-bin/amigo/go.cgi?view=details&search_constraint=terms&depth=0&query=GO:0043069) negative regulation of programmed cell death | 70.4729 | 91 | 0.0403374 |
| Gene Ontology | [visit](http://www.godatabase.org/cgi-bin/amigo/go.cgi?view=details&search_constraint=terms&depth=0&query=GO:0005159) insulin-like growth factor receptor binding | 2.41227 | 7 | 0.0404609 |
| Gene Ontology | [visit](http://www.godatabase.org/cgi-bin/amigo/go.cgi?view=details&search_constraint=terms&depth=0&query=GO:0016655) oxidoreductase activity, acting on NADH or NADPH, quinone or similar compound as acceptor | 8.61526 | 2 | 0.0406986 |
| Gene Ontology | [visit](http://www.godatabase.org/cgi-bin/amigo/go.cgi?view=details&search_constraint=terms&depth=0&query=GO:0002162) dystroglycan binding | 0.516916 | 3 | 0.0406992 |
| Gene Ontology | [visit](http://www.godatabase.org/cgi-bin/amigo/go.cgi?view=details&search_constraint=terms&depth=0&query=GO:0004971) alpha-amino-3-hydroxy-5methyl-4-isoxazole propionate selective glutamate receptor  activity | 0.516916 | 3 | 0.0406992 |
| Gene Ontology | [visit](http://www.godatabase.org/cgi-bin/amigo/go.cgi?view=details&search_constraint=terms&depth=0&query=GO:0007182) common-partner SMAD protein phosphorylation | 0.516916 | 3 | 0.0406992 |
| Gene Ontology | [visit](http://www.godatabase.org/cgi-bin/amigo/go.cgi?view=details&search_constraint=terms&depth=0&query=GO:0008510) sodium:bicarbonate symporter activity | 0.516916 | 3 | 0.0406992 |
| Gene Ontology | [visit](http://www.godatabase.org/cgi-bin/amigo/go.cgi?view=details&search_constraint=terms&depth=0&query=GO:0009991) response to extracellular stimulus | 46.1778 | 63 | 0.0406992 |
| Gene Ontology | [visit](http://www.godatabase.org/cgi-bin/amigo/go.cgi?view=details&search_constraint=terms&depth=0&query=GO:0015220) choline transmembrane transporter activity | 0.516916 | 3 | 0.0406992 |
| Gene Ontology | [visit](http://www.godatabase.org/cgi-bin/amigo/go.cgi?view=details&search_constraint=terms&depth=0&query=GO:0016942) insulin-like growth factor binding protein complex | 0.516916 | 3 | 0.0406992 |
| Gene Ontology | [visit](http://www.godatabase.org/cgi-bin/amigo/go.cgi?view=details&search_constraint=terms&depth=0&query=GO:0021553) olfactory nerve development | 0.516916 | 3 | 0.0406992 |
| Gene Ontology | [visit](http://www.godatabase.org/cgi-bin/amigo/go.cgi?view=details&search_constraint=terms&depth=0&query=GO:0022406) membrane docking | 5.51377 | 12 | 0.0406992 |
| Gene Ontology | [visit](http://www.godatabase.org/cgi-bin/amigo/go.cgi?view=details&search_constraint=terms&depth=0&query=GO:0042156) zinc-mediated transcriptional activator activity | 0.516916 | 3 | 0.0406992 |
| Gene Ontology | [visit](http://www.godatabase.org/cgi-bin/amigo/go.cgi?view=details&search_constraint=terms&depth=0&query=GO:0043184) vascular endothelial growth factor receptor 2  binding | 0.516916 | 3 | 0.0406992 |
| Gene Ontology | [visit](http://www.godatabase.org/cgi-bin/amigo/go.cgi?view=details&search_constraint=terms&depth=0&query=GO:0045715) negative regulation of low-density lipoprotein receptor biosynthetic process | 0.516916 | 3 | 0.0406992 |
| Gene Ontology | [visit](http://www.godatabase.org/cgi-bin/amigo/go.cgi?view=details&search_constraint=terms&depth=0&query=GO:0070601) centromeric sister chromatid cohesion | 0.516916 | 3 | 0.0406992 |
| Gene Ontology | [visit](http://www.godatabase.org/cgi-bin/amigo/go.cgi?view=details&search_constraint=terms&depth=0&query=GO:0070602) regulation of centromeric sister chromatid cohesion | 0.516916 | 3 | 0.0406992 |
| Gene Ontology | [visit](http://www.godatabase.org/cgi-bin/amigo/go.cgi?view=details&search_constraint=terms&depth=0&query=GO:0004714) transmembrane receptor protein tyrosine kinase activity | 11.1998 | 20 | 0.0412374 |
| Gene Ontology | [visit](http://www.godatabase.org/cgi-bin/amigo/go.cgi?view=details&search_constraint=terms&depth=0&query=GO:0010647) positive regulation of cell communication | 72.3682 | 93 | 0.0412374 |
| Gene Ontology | [visit](http://www.godatabase.org/cgi-bin/amigo/go.cgi?view=details&search_constraint=terms&depth=0&query=GO:0021536) diencephalon development | 6.89221 | 14 | 0.0412374 |
| Gene Ontology | [visit](http://www.godatabase.org/cgi-bin/amigo/go.cgi?view=details&search_constraint=terms&depth=0&query=GO:0042471) ear morphogenesis | 11.1998 | 20 | 0.0412374 |
| Gene Ontology | [visit](http://www.godatabase.org/cgi-bin/amigo/go.cgi?view=details&search_constraint=terms&depth=0&query=GO:0051054) positive regulation of DNA metabolic process | 11.1998 | 20 | 0.0412374 |
| Gene Ontology | [visit](http://www.godatabase.org/cgi-bin/amigo/go.cgi?view=details&search_constraint=terms&depth=0&query=GO:0003008) system process | 281.202 | 244 | 0.041348 |
| Gene Ontology | [visit](http://www.godatabase.org/cgi-bin/amigo/go.cgi?view=details&search_constraint=terms&depth=0&query=GO:0004726) non-membrane spanning protein tyrosine phosphatase  activity | 1.37844 | 5 | 0.0413698 |
| Gene Ontology | [visit](http://www.godatabase.org/cgi-bin/amigo/go.cgi?view=details&search_constraint=terms&depth=0&query=GO:0014910) regulation of smooth muscle cell migration | 3.61841 | 9 | 0.0413698 |
| Gene Ontology | [visit](http://www.godatabase.org/cgi-bin/amigo/go.cgi?view=details&search_constraint=terms&depth=0&query=GO:0015271) outward rectifier potassium channel activity | 1.37844 | 5 | 0.0413698 |
| Gene Ontology | [visit](http://www.godatabase.org/cgi-bin/amigo/go.cgi?view=details&search_constraint=terms&depth=0&query=GO:0030169) low-density lipoprotein binding | 3.61841 | 9 | 0.0413698 |
| Gene Ontology | [visit](http://www.godatabase.org/cgi-bin/amigo/go.cgi?view=details&search_constraint=terms&depth=0&query=GO:0033631) cell-cell adhesion mediated by integrin | 1.37844 | 5 | 0.0413698 |
| Gene Ontology | [visit](http://www.godatabase.org/cgi-bin/amigo/go.cgi?view=details&search_constraint=terms&depth=0&query=GO:0043984) histone H4-K16 acetylation | 1.37844 | 5 | 0.0413698 |
| Gene Ontology | [visit](http://www.godatabase.org/cgi-bin/amigo/go.cgi?view=details&search_constraint=terms&depth=0&query=GO:0044455) mitochondrial membrane part | 22.9166 | 12 | 0.0413698 |
| Gene Ontology | [visit](http://www.godatabase.org/cgi-bin/amigo/go.cgi?view=details&search_constraint=terms&depth=0&query=GO:0048617) embryonic foregut morphogenesis | 1.37844 | 5 | 0.0413698 |
| Gene Ontology | [visit](http://www.godatabase.org/cgi-bin/amigo/go.cgi?view=details&search_constraint=terms&depth=0&query=GO:0050684) regulation of mRNA pro-  cessing | 3.61841 | 9 | 0.0413698 |
| Gene Ontology | [visit](http://www.godatabase.org/cgi-bin/amigo/go.cgi?view=details&search_constraint=terms&depth=0&query=GO:0051489) regulation of filopodium assembly | 1.37844 | 5 | 0.0413698 |
| Gene Ontology | [visit](http://www.godatabase.org/cgi-bin/amigo/go.cgi?view=details&search_constraint=terms&depth=0&query=GO:0051491) positive regulation of filopodium assembly | 1.37844 | 5 | 0.0413698 |
| Gene Ontology | [visit](http://www.godatabase.org/cgi-bin/amigo/go.cgi?view=details&search_constraint=terms&depth=0&query=GO:0090002) establishment of protein localization in plasma membrane | 1.37844 | 5 | 0.0413698 |
| Gene Ontology | [visit](http://www.godatabase.org/cgi-bin/amigo/go.cgi?view=details&search_constraint=terms&depth=0&query=GO:0090150) establishment of protein localization in membrane | 1.37844 | 5 | 0.0413698 |
| Gene Ontology | [visit](http://www.godatabase.org/cgi-bin/amigo/go.cgi?view=details&search_constraint=terms&depth=0&query=GO:0007243) intracellular protein kinase cascade | 102.866 | 127 | 0.0418199 |
| Gene Ontology | [visit](http://www.godatabase.org/cgi-bin/amigo/go.cgi?view=details&search_constraint=terms&depth=0&query=GO:0014911) positive regulation of smooth muscle cell migration | 1.89536 | 6 | 0.0418199 |
| Gene Ontology | [visit](http://www.godatabase.org/cgi-bin/amigo/go.cgi?view=details&search_constraint=terms&depth=0&query=GO:0023014) signal transmission via phosphorylation event | 102.866 | 127 | 0.0418199 |
| Gene Ontology | [visit](http://www.godatabase.org/cgi-bin/amigo/go.cgi?view=details&search_constraint=terms&depth=0&query=GO:0030858) positive regulation of epithelial cell differentiation | 1.89536 | 6 | 0.0418199 |
| Gene Ontology | [visit](http://www.godatabase.org/cgi-bin/amigo/go.cgi?view=details&search_constraint=terms&depth=0&query=GO:0045022) early endosome to late endosome transport | 1.89536 | 6 | 0.0418199 |
| Gene Ontology | [visit](http://www.godatabase.org/cgi-bin/amigo/go.cgi?view=details&search_constraint=terms&depth=0&query=GO:0051721) protein phosphatase 2A binding | 1.89536 | 6 | 0.0418199 |
| Gene Ontology | [visit](http://www.godatabase.org/cgi-bin/amigo/go.cgi?view=details&search_constraint=terms&depth=0&query=GO:0060325) face morphogenesis | 1.89536 | 6 | 0.0418199 |
| Gene Ontology | [visit](http://www.godatabase.org/cgi-bin/amigo/go.cgi?view=details&search_constraint=terms&depth=0&query=GO:0007584) response to nutrient | 29.4642 | 43 | 0.0418439 |
| Gene Ontology | [visit](http://www.godatabase.org/cgi-bin/amigo/go.cgi?view=details&search_constraint=terms&depth=0&query=GO:0044421) extracellular region part | 172.478 | 143 | 0.0418439 |
| Gene Ontology | [visit](http://www.godatabase.org/cgi-bin/amigo/go.cgi?view=details&search_constraint=terms&depth=0&query=GO:0016791) phosphatase activity | 43.7655 | 60 | 0.0418662 |
| Gene Ontology | [visit](http://www.godatabase.org/cgi-bin/amigo/go.cgi?view=details&search_constraint=terms&depth=0&query=GO:0022836) gated channel activity | 53.2423 | 71 | 0.0420747 |
| Gene Ontology | [visit](http://www.godatabase.org/cgi-bin/amigo/go.cgi?view=details&search_constraint=terms&depth=0&query=GO:0030111) regulation of Wnt receptor signaling pathway | 15.8521 | 26 | 0.0435536 |
| Gene Ontology | [visit](http://www.godatabase.org/cgi-bin/amigo/go.cgi?view=details&search_constraint=terms&depth=0&query=GO:0045165) cell fate commitment | 24.6397 | 37 | 0.0443694 |
| Gene Ontology | [visit](http://www.godatabase.org/cgi-bin/amigo/go.cgi?view=details&search_constraint=terms&depth=0&query=GO:0051348) negative regulation of transferase activity | 19.8151 | 31 | 0.0445417 |
| Gene Ontology | [visit](http://www.godatabase.org/cgi-bin/amigo/go.cgi?view=details&search_constraint=terms&depth=0&query=GO:0043233) organelle lumen | 324.106 | 364 | 0.045116 |
| Gene Ontology | [visit](http://www.godatabase.org/cgi-bin/amigo/go.cgi?view=details&search_constraint=terms&depth=0&query=GO:0010828) positive regulation of glucose transport | 4.30763 | 10 | 0.045477 |
| Gene Ontology | [visit](http://www.godatabase.org/cgi-bin/amigo/go.cgi?view=details&search_constraint=terms&depth=0&query=GO:0048546) digestive tract morphogenesis | 4.30763 | 10 | 0.045477 |
| Gene Ontology | [visit](http://www.godatabase.org/cgi-bin/amigo/go.cgi?view=details&search_constraint=terms&depth=0&query=GO:0007163) establishment or maintenance of cell polarity | 9.13218 | 17 | 0.045839 |
| Gene Ontology | [visit](http://www.godatabase.org/cgi-bin/amigo/go.cgi?view=details&search_constraint=terms&depth=0&query=GO:0017015) regulation of transforming growth factor beta receptor signaling pathway | 9.13218 | 17 | 0.045839 |
| Gene Ontology | [visit](http://www.godatabase.org/cgi-bin/amigo/go.cgi?view=details&search_constraint=terms&depth=0&query=GO:0006818) hydrogen transport | 11.5445 | 4 | 0.0462546 |
| Gene Ontology | [visit](http://www.godatabase.org/cgi-bin/amigo/go.cgi?view=details&search_constraint=terms&depth=0&query=GO:0048754) branching morphogenesis of a tube | 16.7136 | 27 | 0.0462546 |
| Gene Ontology | [visit](http://www.godatabase.org/cgi-bin/amigo/go.cgi?view=details&search_constraint=terms&depth=0&query=GO:0016651) oxidoreductase activity, acting on NADH or NADPH | 14.4736 | 6 | 0.0463895 |
| Gene Ontology | [visit](http://www.godatabase.org/cgi-bin/amigo/go.cgi?view=details&search_constraint=terms&depth=0&query=GO:0031099) regeneration | 15.1629 | 25 | 0.0463895 |
| Gene Ontology | [visit](http://www.godatabase.org/cgi-bin/amigo/go.cgi?view=details&search_constraint=terms&depth=0&query=GO:0033365) protein localization in organelle | 36.3564 | 51 | 0.0464884 |
| Gene Ontology | [visit](http://www.godatabase.org/cgi-bin/amigo/go.cgi?view=details&search_constraint=terms&depth=0&query=GO:0030509) BMP signaling pathway | 11.3721 | 20 | 0.0470274 |
| Gene Ontology | [visit](http://www.godatabase.org/cgi-bin/amigo/go.cgi?view=details&search_constraint=terms&depth=0&query=GO:0061053) somite development | 8.44296 | 16 | 0.0472494 |
| Gene Ontology | [visit](http://www.godatabase.org/cgi-bin/amigo/go.cgi?view=details&search_constraint=terms&depth=0&query=GO:0005740) mitochondrial envelope | 76.5035 | 57 | 0.0472525 |
| Gene Ontology | [visit](http://www.godatabase.org/cgi-bin/amigo/go.cgi?view=details&search_constraint=terms&depth=0&query=GO:0015078) hydrogen ion transmembrane transporter activity | 15.8521 | 7 | 0.0472525 |
| Gene Ontology | [visit](http://www.godatabase.org/cgi-bin/amigo/go.cgi?view=details&search_constraint=terms&depth=0&query=GO:0032587) ruffle membrane | 4.99685 | 11 | 0.0478351 |
| Gene Ontology | [visit](http://www.godatabase.org/cgi-bin/amigo/go.cgi?view=details&search_constraint=terms&depth=0&query=GO:0044212) DNA regulatory region binding | 21.5382 | 33 | 0.0478351 |
| Gene Ontology | [visit](http://www.godatabase.org/cgi-bin/amigo/go.cgi?view=details&search_constraint=terms&depth=0&query=GO:0048813) dendrite morphogenesis | 4.99685 | 11 | 0.0478351 |
| Gene Ontology | [visit](http://www.godatabase.org/cgi-bin/amigo/go.cgi?view=details&search_constraint=terms&depth=0&query=GO:0004722) protein serinethreonine phosphatase activity | 7.75374 | 15 | 0.0478998 |
| Gene Ontology | [visit](http://www.godatabase.org/cgi-bin/amigo/go.cgi?view=details&search_constraint=terms&depth=0&query=GO:0007090) regulation of S phase of mitotic cell cycle | 3.10149 | 8 | 0.0478998 |
| Gene Ontology | [visit](http://www.godatabase.org/cgi-bin/amigo/go.cgi?view=details&search_constraint=terms&depth=0&query=GO:0016010) dystrophin-associated glycoprotein complex | 3.10149 | 8 | 0.0478998 |
| Gene Ontology | [visit](http://www.godatabase.org/cgi-bin/amigo/go.cgi?view=details&search_constraint=terms&depth=0&query=GO:0031974) membrane-enclosed lumen | 330.309 | 370 | 0.0478998 |
| Gene Ontology | [visit](http://www.godatabase.org/cgi-bin/amigo/go.cgi?view=details&search_constraint=terms&depth=0&query=GO:0035116) embryonic hindlimb morphogenesis | 3.10149 | 8 | 0.0478998 |
| Gene Ontology | [visit](http://www.godatabase.org/cgi-bin/amigo/go.cgi?view=details&search_constraint=terms&depth=0&query=GO:0042596) fear response | 3.10149 | 8 | 0.0478998 |
| Gene Ontology | [visit](http://www.godatabase.org/cgi-bin/amigo/go.cgi?view=details&search_constraint=terms&depth=0&query=GO:0046906) tetrapyrrole binding | 22.572 | 12 | 0.0478998 |
| Gene Ontology | [visit](http://www.godatabase.org/cgi-bin/amigo/go.cgi?view=details&search_constraint=terms&depth=0&query=GO:0048660) regulation of smooth muscle cell proliferation | 7.75374 | 15 | 0.0478998 |
| Gene Ontology | [visit](http://www.godatabase.org/cgi-bin/amigo/go.cgi?view=details&search_constraint=terms&depth=0&query=GO:0071695) anatomical structure maturation | 3.10149 | 8 | 0.0478998 |
| Gene Ontology | [visit](http://www.godatabase.org/cgi-bin/amigo/go.cgi?view=details&search_constraint=terms&depth=0&query=GO:0008324) cation transmembrane  transporter activity | 94.4233 | 117 | 0.048075 |
| Gene Ontology | [visit](http://www.godatabase.org/cgi-bin/amigo/go.cgi?view=details&search_constraint=terms&depth=0&query=GO:0045767) regulation of anti-apoptosis | 7.06452 | 14 | 0.0487173 |
| Gene Ontology | [visit](http://www.godatabase.org/cgi-bin/amigo/go.cgi?view=details&search_constraint=terms&depth=0&query=GO:0051219) phosphoprotein binding | 5.68607 | 12 | 0.0487173 |
| Gene Ontology | [visit](http://www.godatabase.org/cgi-bin/amigo/go.cgi?view=details&search_constraint=terms&depth=0&query=GO:0020037) heme binding | 21.1935 | 11 | 0.0489141 |
| Gene Ontology | [visit](http://www.godatabase.org/cgi-bin/amigo/go.cgi?view=details&search_constraint=terms&depth=0&query=GO:0005913) cell-cell adherens junction | 6.3753 | 13 | 0.0489385 |
| Gene Ontology | [visit](http://www.godatabase.org/cgi-bin/amigo/go.cgi?view=details&search_constraint=terms&depth=0&query=GO:0015082) di-, tri-valent inorganic cation transmembrane transporter activity | 6.3753 | 13 | 0.0489385 |
| Gene Ontology | [visit](http://www.godatabase.org/cgi-bin/amigo/go.cgi?view=details&search_constraint=terms&depth=0&query=GO:0001666) response to hypoxia | 25.6735 | 38 | 0.0490651 |
| Gene Ontology | [visit](http://www.godatabase.org/cgi-bin/amigo/go.cgi?view=details&search_constraint=terms&depth=0&query=GO:0017111) nucleoside-triphosphatase activity | 126.3 | 152 | 0.0490651 |
| Gene Ontology | [visit](http://www.godatabase.org/cgi-bin/amigo/go.cgi?view=details&search_constraint=terms&depth=0&query=GO:0016818) hydrolase activity, acting on acid anhydrides, in phosphorus-containing anhydrides | 131.814 | 158 | 0.0492847 |
